# Japanese clinical practice guidelines for vascular anomalies 2017

**DOI:** 10.1111/ped.14077

**Published:** 2020-03-22

**Authors:** Hidefumi Mimura, Sadanori Akita, Akihiro Fujino, Masatoshi Jinnin, Mine Ozaki, Keigo Osuga, Hiroki Nakaoka, Eiichi Morii, Akira Kuramochi, Yoko Aoki, Yasunori Arai, Noriko Aramaki, Masanori Inoue, Yuki Iwashina, Tadashi Iwanaka, Shigeru Ueno, Akihiro Umezawa, Michio Ozeki, Junko Ochi, Yoshiaki Kinoshita, Masakazu Kurita, Shien Seike, Nobuyuki Takakura, Masataka Takahashi, Takao Tachibana, Kumiko Chuman, Shuji Nagata, Mitsunaga Narushima, Yasunari Niimi, Shunsuke Nosaka, Taiki Nozaki, Kazuki Hashimoto, Ayato Hayashi, Satoshi Hirakawa, Atsuko Fujikawa, Yumiko Hori, Kentaro Matsuoka, Hideki Mori, Yuki Yamamoto, Shunsuke Yuzuriha, Naoaki Rikihisa, Shoji Watanabe, Shinichi Watanabe, Tatsuo Kuroda, Shunsuke Sugawara, Kosuke Ishikawa, Satoru Sasaki

**Affiliations:** ^1^ Department of Radiology St Marianna University School of Medicine Kawasaki Japan; ^2^ Department of Plastic Surgery, Wound Repair and Regeneration School of Medicine Fukuoka University Fukuoka Japan; ^3^ Division of Surgery National Center for Child Health and Development Tokyo Japan; ^4^ Department of Reproductive Biology Center for Regenerative Medicine National Center for Child Health and Development Tokyo Japan; ^5^ Division of Radiology National Center for Child Health and Development Tokyo Japan; ^6^ Department of Plastic and Reconstructive Surgery Keio University School of Medicine Tokyo Japan; ^7^ Department of Radiology Keio University School of Medicine Tokyo Japan; ^8^ Department of Pediatric Surgery Keio University School of Medicine Tokyo Japan; ^9^ Department of Diagnostic Radiology National Cancer Center Hospital Tokyo Japan; ^10^ Department of Pediatric Surgery The University of Tokyo Hospital Tokyo Japan; ^11^ Department of Plastic and Reconstructive Surgery The University of Tokyo Hospital Tokyo Japan; ^12^ Department of Dermatology Kanto Central Hospital Tokyo Japan; ^13^ Department of Neuroendovascular Therapy St Luke's International Hospital Tokyo Japan; ^14^ Department of Radiology St Luke's International Hospital Tokyo Japan; ^15^ Department of Dermatology Teikyo University School of Medicine Tokyo Japan; ^16^ Department of Dermatology Wakayama Medical University Wakayama Japan; ^17^ Department of Plastic and Reconstructive, Aesthetic Surgery Kyorin University School of Medicine Mitaka Japan; ^18^ Department of Diagnostic and Interventional Radiology Osaka University Graduate School of Medicine Suita Japan; ^19^ Department of Pathology Osaka University Graduate School of Medicine Suita Japan; ^20^ Department of Plastic Surgery Osaka University Graduate School of Medicine Suita Japan; ^21^ Department of Signal Transduction Research Institute for Microbial Diseases Osaka University Suita Japan; ^22^ Department of Plastic Surgery Ehime University Hospital Toon Japan; ^23^ Department of Dermatology Saitama Medical University Irumagun Japan; ^24^ Department of Medical Genetics Tohoku University School of Medicine Sendai Japan; ^25^ Department of Diagnostic Radiology Tohoku University Graduate School of Medicine Sendai Japan; ^26^ Department of Pediatric Surgery Tokai University School of Medicine Isehara Japan; ^27^ Department of Pediatrics Gifu University Graduate School of Medicine Gifu Japan; ^28^ Department of Department of Pediatric Surgery Niigata University Graduate School of Medical and Dental Sciences Niigata Japan; ^29^ Department of Dermatology Osaka Red Cross Hospital Osaka Japan; ^30^ Department of Radiology Kurume University School of Medicine Kurume Japan; ^31^ Department of Plastic and Reconstructive Surgery Graduate School of Medicine Mie University Tsu Japan; ^32^ Department of Plastic and Reconstructive Surgery Juntendo University Urayasu Hospital Urayasu Japan; ^33^ Department of Dermatology Hamamatsu University School of Medicine Hamamatsu Japan; ^34^ Department of Pathology Dokkyo Medical University Saitama Medical Center Koshigaya Japan; ^35^ Department of Plastic and Reconstructive Surgery Shinshu University School of Medicine Matsumoto Japan; ^36^ Department of Plastic and Reconstructive Surgery Oyumino Central Hospital Chiba Japan; ^37^ Department of Plastic and Reconstructive Surgery Saitama Children's Medical Center Saitama Japan; ^38^ Department of Plastic and Reconstructive Surgery Faculty of Medicine and Graduate School of Medicine Hokkaido University Sapporo Japan; ^39^ Department of Plastic and Reconstructive Surgery Center for Vascular Anomalies Tonan Hospital Sapporo Japan

**Keywords:** clinical practice, guidelines, hemangioma, vascular anomalies, vascular malformation

## Abstract

The objective was to prepare guidelines to perform the current optimum treatment by organizing effective and efficient treatments of hemangiomas and vascular malformations, confirming the safety, and systematizing treatment, employing evidence‐based medicine (EBM) techniques and aimed at improvement of the outcomes. Clinical questions (CQs) were decided based on the important clinical issues. For document retrieval, key words for literature searches were set for each CQ and literature published from 1980 to the end of September 2014 was searched in Pubmed, Cochrane Library, and Japana Centra Revuo Medicina (JCRM). The strengths of evidence and recommendations acquired by systematic reviews were determined following the Medical Information Network Distribution System (MINDS) technique. A total of 33 CQs were used to compile recommendations and the subjects included efficacy of resection, sclerotherapy/embolization, drug therapy, laser therapy, radiotherapy, and other conservative treatment, differences in appropriate treatment due to the location of lesions and among symptoms, appropriate timing of treatment and tests, and pathological diagnosis deciding the diagnosis. Thus, the Japanese Clinical Practice Guidelines for Vascular Anomalies 2017 have been prepared as the evidence‐based guidelines for the management of vascular anomalies.

The etiology of vascular anomalies on the body surface and in soft tissue are mostly unclear and no fundamental treatment methods have been established. Many patients visit many medical institutions seeking an expert, being a disadvantage in treatment. Hemangiomas and vascular malformations are frequently termed ‘hemangioma’, but these are different diseases in the ISSVA classification proposed by the International Society for Study of Vascular Anomalies (ISSVA),^1^, ^2^ and this classification has been internationally standardized.

“Clinical Practice Guidelines for Vascular Anomalies 2013” (1st edition)^3^ target general practitioners and the general public, and were prepared aiming at organizing effective and efficient treatments for hemangiomas/vascular malformations, confirming the safety, and systematizing treatment using evidence‐based medicine (EBM) techniques. The organization responsible for preparation was the Health, Labour and Welfare Sciences Research Grants (Research on Measures for Intractable Diseases), Research Committee for “Intractable Vascular Anomalies,” and the main committee members were selected from academic societies of plastic surgery and radiology mainly treating hemangiomas and vascular malformations: the Japanese Society of Plastic and Reconstructive Surgery and Japanese Society of Interventional Radiology, and the guidelines were prepared by them.

‘‘Clinical Practice Guidelines for Vascular Anomalies 2017” were prepared as a revised edition of the “Clinical Practice Guidelines for Vascular Anomalies 2013.” The organization responsible for preparation was the Health, Labour and Welfare Sciences Research Grants (Research on Policy Planning and Evaluation for Rare and Intractable Diseases), Research Committee for Intractable Vascular Anomalies, and the differences from the previous guidelines are setting the objective at summarizing opinions from related academic societies by inviting many committee members from dermatologists, pediatric surgeons, pediatricians, radiologists (diagnostic radiology), and basic researchers including the pathology, molecular biology, and epidemiology fields, in addition to plastic surgeons and radiologists (interventional radiology). Since the guidelines were prepared following the reformed “Minds Handbook for Clinical Practice Guideline Development 2014”^4^ and “Minds Manual for Clinical Practice Guideline Development Ver.1.0‐2.0,”^5^, ^6^ it was fully revised.

The original text of the guidelines (Japanese version) is comprised of Reviews and Clinical Questions (CQs), but only CQs are presented in this report.

## Purpose of the guidelines

The objective was to prepare guidelines to perform the current optimum treatment by organizing effective and efficient treatments of hemangiomas and vascular malformations, confirming the safety, and systematizing treatment, employing the EBM techniques and aimed at improvement of the following outcomes: Pain, swelling, esthetic impairment, and functional disorder.

## Materials and methods

### Organization

For the Guidelines Executive Committee members, representatives of the plastic surgery, dermatology, radiology, pediatric surgery, and basic science fields were selected. The guidelines preparation group and systematic review team for preparation of CQs and recommendations were comprised of 4 groups: groups in charge of arteriovenous malformation (AVM), venous malformation (VM), combined type, and syndrome, in charge of capillary malformation (CM) and infantile hemangioma, in charge of the lymphatic malformation (lymphangioma) (LM), and in charge of the basic field. To the group in charge of AVM, VM, combined type, and syndrome, plastic surgeons and radiologists were mainly assigned. To the group in charge of CM and infantile hemangioma, plastic surgeons and dermatologists were mainly assigned. To the group in charge of the lymphatic system, pediatric surgeons, plastic surgeons, and pediatricians were mainly assigned. The Reviews of the guidelines were also prepared by those selected from each group. Pathologists and molecular biologists were in charge of the Reviews of the basic fields.

### Preparation process

The guidelines were revised following the “Minds Handbook for Clinical Practice Guideline Development 2014” and “Minds Manual for Clinical Practice Guideline Development Ver.1.0‐2.0.”

CQs were decided based on the following important clinical issues: (i) Efficacy of resection, (ii) efficacy of sclerotherapy/embolization, (iii) efficacy of drug therapy, laser therapy, radiotherapy, and other conservative treatment, (iv) differences in appropriate treatment due to the location of lesions, (v) differences in appropriate treatment among symptoms, (vi) appropriate timing of treatment and tests, and (vii) pathological diagnosis deciding the diagnosis.

For document retrieval, key words for literature searches were set for each CQ and literature published from 1980 to the end of September 2014 was searched in Pubmed, Cochrane Library, and Japana Centra Revuo Medicina (JCRM). Literature search was requested to the Japan Medical Library Association. For decisions on CQs and recommendations lacking evidence or having weak evidence, discussion and agreement in the preparation group were reflected.

The strengths of evidence and recommendations acquired by systematic reviews were determined following the Minds technique as described below and this follows the GRADE guidelines preparation method.^7^, ^8^

### Determination of the strength of evidence of the body of evidence

The Strength of Evidence of the Body of Evidence was determined according to ‘Minds Handbook for Clinical Practice Guideline Development 2014’ (Table 1).

In the case of RCTs, the score “A (strong)” is given at the start of evaluation, and the final score might be downgraded to B, C, or D, according to the results of evaluation of five items, including risk of bias, inconsistency in results, indirectness of evidence, data imprecision, and high possibility of publication bias. In the case of observational studies, the score “C (weak)” is given at the start of evaluation, and five items lowering the strength are evaluated similarly as for RCTs. In addition, three items, including large effect with no confounding factors, dose–response gradient, and possible confounding factors, are weaker than actual effects increasing the strength are evaluated as well.

**Table 1:** Recommendation Grade and Definition of the Strength of Body of Evidence in Evaluation of Systematic Review

Recommendation grade
1 strongly recommended
2 weakly recommended (suggested)
Definition of the Strength of Body of Evidence in Evaluation of Systematic Review
A (strong): strongly confident of the estimate of effect
B (moderate): moderately confident of the estimate of effect
C (weak): limited confidence of the estimate of effect
D (very weak): very little confident of the estimate of effect

### Presentation of the strength of recommendations

The strength of recommendation was also determined according to “Minds Handbook for Clinical Practice Guideline Development 2014” (Table 1).

The strength of recommendations is usually presented in two ways: “1”: strongly recommended, and “2”: weakly recommended (suggested). If the strength of recommendations cannot be determined by any means, it is occasionally presented as “no definite recommendation can be made.” Recommendations will be entered as follows by indicating the strength of evidence (A, B, C, D) with the strength of recommendations “1”: strong or “2”: weak.

### Finalization

Preparation of the draft guidelines was completed in December 2016 and review was requested to the Japanese Society of Plastic and Reconstructive Surgery, Japanese Dermatological Association, Japan Radiology Society, Japanese Society of Interventional Radiology, Japanese Society of Pediatric Surgeons, and Japanese Society of Pathology between December 2016 and January 2017, and corrections were made based on the results of the reviews. In addition, from December 2016 to January 2017, the guidelines were disclosed on the home page of the Research Committee for ‘Intractable Vascular Anomalies’ and public comments were collected. The draft guidelines were presented to two related patient organizations, ‘the Patients Association of Vascular Anomalies’ and ‘the Patients Association of Combined Vascular Malformations’ and comments were received. Based on these, the draft guidelines were revised and CQs, recommendations, and explanations were completed. It was finalized in March 2017.

## Results

### CQs and recommendations

#### CQ1: What are the guidelines for the time to begin treatment for AVM?

*Recommendation*: It is necessary to judge the time to begin endovascular or surgical treatment for AVM individually by evaluating the stage of symptoms and lesion extent and in consideration of the risk of complications.

*Strength of recommendation*: 2 (weak)

*Evidence*: D (very weak)

*Comments*: As a result of primary screening, 92, 3, and 27 papers were extracted from PubMed, JCRM, and Cochrane Library, respectively, and, as a result of secondary screening, 37 and 3 papers were extracted from PubMed and JCRM, respectively. However, as all these references were observational or case series studies, the strength of evidence is rated as “D (very weak).”

There has been no report in which the time to begin treatment for AVM in itself was the endpoint, and only some reports described the view on the time to begin treatment in the discussion. Therefore, as it is difficult to objectively evaluate the validity of the time to begin treatment, we determined whether guidelines can be derived from the patient age, lesion site, symptoms, clinical stage, effectiveness of treatment, frequency of complications in each report.

In the reports^9^, ^10^ on the treatment for AVM, symptomatic AVM is primarily addressed, and treatment can be suspended (follow‐up) while the lesion remains asymptomatic. However, as AVM often progresses when left untreated, it is considered important to begin the treatment at an appropriate time depending on the stage of symptoms. In addition, as there is the tendency that the response rate decreases, and the complication rate increases, with progression of symptoms, some reports from particular pediatric institutions where patients are concentrated recommend early therapeutic intervention in relatively “early” or “mild” stages without waiting for progression of the disease.

Localized lesions may be radically treated by early intervention.^11^ Among endovascular procedures, the response (cure) rate tends to be high by ethanol embolization, but as the complication rate is also high.^12^, ^13^ By surgery, localized lesions are unlikely to recur if they are completely resected, although adverse events, such as postoperative cicatrization/deformation or functional impairment, have been seldom discussed.^10^ On the other hand, in diffuse lesions, limitations of effectiveness, such as recurrence and persistence and the risk of treatment, are higher by both endovascular treatment and surgery, with the risks outweighing benefits.^13^ It should also be considered that children, in particular, are not mentally ready to accept such invasive treatments.^14^

As discussed above, it is difficult to give guidelines for the time to begin treatment for AVM at present, and individual judgment is necessary depending on the symptom stage and lesion extent in consideration of the complication risk.

#### CQ2: Is recurrence (regrowth) after resection of AVM more frequent by wound closure with a skin graft than by reconstruction using a flap?

*Recommendation*: Whether recurrence (regrowth) is more frequent by wound closure with a skin graft compared with reconstruction using a flap is unclear.

*Strength of recommendation*: 2 (weak)

*Evidence*: D (very weak)

*Comments*: The number of references retrieved by searches using keywords was 40 from JCRM, 75 from PubMed, and 0 from Cochrane, and 39 were extracted by secondary screening. For AVM of a certain size, reconstruction is necessary after resection, and wound closure by skin grafting or reconstruction using a flap is performed according to common reconstruction methods for tissue defects. The reports discussing reconstruction after resection of AVM that we could retrieve were all descriptive studies (case reports or case series studies). Therefore, the evidence level of all these references is D (very weak).

The concept of regulating flap,^15^, ^16^ that reconstruction using a free flap controls the recurrence or regrowth after resection of AVM, has been proposed. However, no report has evaluated whether free flaps^16^, ^17^, ^18^, ^19^, ^20^, ^21^, ^22^, ^23^, ^24^, ^25^, ^26^, ^27^, ^28^, ^29^, ^30^, ^31^, ^32^, ^33^, ^34^, ^35^, ^36^, ^37^ and other flap types^17^, ^19^, ^25^, ^28^, ^38^, ^39^, ^40^, ^41^, ^42^, ^43^, ^44^, ^45^, ^46^, ^47^, ^48^, ^49^ clearly prevent the recurrence or regrowth compared with skin grafts.^21^, ^23^, ^50^, ^51^, ^52^

According to the present knowledge about the recurrence or regrowth after resection of AVM,^15^, ^16^, ^39^, ^53^ whether AVM can be completely resected is important, and, concerning cases in which complete resection is difficult, it has been reported that the hemodynamics in the residual lesions contributes to the recurrence and regrowth, and that it can be controlled by a flap with rich blood flow.

#### CQ3: Is proximal ligation/coil embolization of the feeding artery of AVM effective?

*Recommendation*: The therapeutic effect of ligation/coil embolization of the feeding artery on the proximal side may be poor, and the possibility of recurrence may be high. In addition, in the event of recurrence, treatment may become difficult due to the development of collateral vessels. Therefore, these procedures are recommended to be avoided, in principle.

*Strength of recommendation*: 2 (weak)

*Evidence*: D (very weak)

*Comments*: As a result of secondary screening, 1 and 14 papers were extracted from PubMed and JCRM, respectively, and were reviewed. As a result, all these papers were case reports. In addition, 6 papers retrieved by manual searches were also reviewed, but they were all case series studies at the maximum. Therefore, the strength of evidence as a collection of literature concerning this CQ is D (very weak).

To summarize the evaluation of this collection of papers, AVM was treated by proximal ligation/coil embolization of the feeding artery, but as there have been reports of recurrence following the development of collateral channels (reports of cases of the occurrence of unfavorable situations), the treatment is recommended to be avoided, but the evidence level of this recommendation is low as mentioned above.

The objective of embolization for AVM is obliteration of the nidus, and embolization at or near the nidus is necessary as much as possible. If ligation/coil embolization of the feeding artery is performed on the proximal/central side, the nidus is not obliterated, and the development of several collateral channels is promoted. In many cases, the collateral channels are thin, complicated, and markedly tortuous, and trans‐catheter treatment is often difficult.

Wu *et al*.^14^ performed proximal ligation in 9 of 29 patients treated for AVM of the auricle but that the condition was exacerbated in all patients with 8 requiring auricular resection and 1 requiring additional treatment. They excluded proximal ligation from treatment options for AVM, because it makes subsequent trans‐catheter treatments difficult.

Slaba *et al*.^54^ evaluated 25 patients with AVM of the tongue and reported that 3 of the 12 symptomatic patients who underwent ipsilateral external carotid artery ligation at another facility showed marked development of collateral channels.

Other reports include those of a case in which a large number of collateral channels developed as a result of ligation of the feeding artery for AVM of the shoulder with serious complications including high‐output heart failure,^55^ 3 cases in which proximal ligation/embolization was performed for AVM of the limbs and pelvis, collateral vessels developed, but the condition was controlled by multidisciplinary treatment consisting of trans‐catheter treatment and direct puncture sclerotherapy,^56^ and several cases in which external carotid artery ligation was performed for AVM of the head and neck, but the subsequent treatment was difficult.

Suyama *et al*.^35^ reported a case in which AVM of the auricle, treated by proximal embolization using coils and gelatin sponge, recurred and was treated again by ligation at a proximal part of the artery, but the lesion recurred again. Also, Aikawa *et al*.^57^ reported a case of intrapelvic AVM that underwent coil embolization of the left ovarian artery and left internal iliac artery but showed little change in the area of the nidus or the state of arterial or venous dilatation. In addition, Yamamoto *et al*.^58^ reported a case in which TAE was performed for AVM of the mandible via the maxillary artery, facial artery, lingual artery, and ocular artery but was not effective due to the development of collateral channels from the internal carotid artery and vertebral artery.

As observed above, it is recommended not to select proximal/central ligation/coil embolization as a treatment for AVM. However, AV fistulas with direct connection of a large artery and a vessel may be treated by coil embolization if the shunt area is directly accessible with a catheter. Proximal coil embolization may also be accepted as preoperative embolization, but careful evaluation of its indications is necessary, and embolization at a site near the shunt is necessary to leave the room for catheter insertion in the event of future recurrence.

#### CQ4: What is the appropriate timing for embolization before resection of AVM?

*Recommendation*: It is recommended to perform resection within 3 days (72 h) after embolization. If the interval prolongs, the risk of massive intraoperative hemorrhage may increase due to recanalization of the embolized vessel and development of collateral channels. In addition, surgery has been reported to be made difficult by enlargement of the lesion after embolization.

*Strength of recommendation*: 2 (weak)

*Evidence*: D (very weak)

*Comments*: While it is difficult to generalize the therapeutic approach as it varies with the affected area and extent of the lesion, there were a few reports that preoperative embolization was useful for the treatment of AVM of the head and neck region.

As a result of secondary screening, 10 and 3 papers from PubMed and JCRM, respectively, were reviewed. All the papers selected by this screening procedure were case reports or case series, and the strength of evidence is “D (very weak).” Mentions about the timing of preoperative embolization and volume of hemorrhage also varied among the papers. Although it is difficult to draw a conclusion, among the papers that mentioned specific timing of preoperative embolization and volume of hemorrhage, Deng *et al*.^59^ performed embolization within 48–72 h before surgery in 16 patients with maxillofacial AVM and reported that the volume of hemorrhage was ≤200 mL in all patients and that there were no complications. Erdmann *et al*.^60^ performed embolization within 24 h before surgery in 4 patients with head and neck AVM, and the lesion was resected with hemorrhage of ≤100 mL in 3. It is recommended to perform resection within 72 h to prevent increases in difficulty of resection due to inflammation after embolization.

There have also been reports that embolization was performed intraoperatively or within a few days before surgery, resulting in decreases in the volume of hemorrhage or favorable long‐term outcomes. Most papers reported no or only mild complications, but as for relatively severe complications, Goldberg *et al*.^61^ reported temporary visual impairment in 2 of the 3 patients with orbital AVM.

Factors that affect the appropriate timing of preoperative embolization include recanalization of the target vessel, development of collateral channels, and swelling and reactive changes after embolization, which make surgery difficult. To avoid the effects of these phenomena, many papers supported relatively early resection, i.e., within 72 h after embolization. Clinically, also, there is no benefit in taking a long interval, and it is considered valid to recommend resection within 72 h after embolization.

In conclusion, adequate control of hemorrhage may be achieved with fewer complications by performing vascular embolization within a few days before surgery, but no sufficient evidence that support this view has been provided.

#### CQ5: What treatments are appropriate for the maxillo‐mandibular AVM?

*Recommendation*: Although surgery alone is not recommended, a combination of surgery with endovascular embolization (including sclerotherapy) can be recommended depending on the case.

Radiotherapy is not recommended.

Endovascular embolization (including sclerotherapy) alone or as a preoperative treatment can be recommended.

*Strength of recommendation*: 2 (weak)

*Evidence*: D (very weak)

*Comments*: AVM of the maxilla and mandible is a rare disorder. Most of the literature is reports of a small number of cases except a few case series reported from some special institutions. Only 5 reports^62^, ^63^, ^64^, ^65^, ^66^ of a series of 10 or more cases were retrieved by the search of PubMed. Because there is no cohort study or randomized trial comparing various treatments, strong evidence regarding the best treatment is lacking.

Maxillo‐mandibular AVM may involve the maxilla, mandible, or both, and it often presents with massive oral hemorrhage around the age of 10 years when milk teeth are lost, but may also be detected due to swelling of the soft tissue.

According to Persky *et al*.,^62^ embolization alone resulted in cure in 42%, improvement in 16%, and stabilization of symptoms in 23% of the 26 patients with a maxillo‐mandibular AVM. Liu *et al*.^63^ treated 25 patients by transarterial or transvenous embolization alone or in combination with curettage and reported anatomical cure in 14 and clinical cure in 21. Chen *et al*.^65^ treated 15 patients by bone wax packing (BWP) alone in 4, transarterial embolization (TAE) + BWP in 3, TAE + resection in 4, and TAE + radiotherapy + resection in 4 and reported clinical cure in 14.

The following are considered as treatment options for AVM of the maxilla and mandible.

A: Surgical treatment

A‐1: Resection and reconstruction
A‐2: Curettage
A‐3: Bone wax packing

B: Endovascular embolization (including sclerotherapy)

B‐1: Transarterial embolization
B‐2: Transvenous embolization
B‐3: Embolization by direct puncture

C: Combination of A and B

D: Radiotherapy

The literature is mostly about B, i.e., endovascular embolization (including sclerotherapy) alone, or surgical treatment after B. There was no report of case series of surgical treatment alone, but there was only 1 report^65^ of case series of surgery + radiotherapy. Surgery alone and radiotherapy are generally not recommended. Endovascular embolization is performed by various approaches including transarterial and transvenous routes and direct puncture, sometimes, in combination. Concerning embolic agents, PVA and Gelfoam® (Pfizer, New York, NY, USA) are used for embolization as an adjuvant therapy immediately before surgery as they allow recanalization. Cyanoacrylate liquid embolic agents are considered effective for embolization performed preoperatively or alone in expectation of a long‐term occlusive effect.^64^, ^67^, ^68^ Coils are often used for transvenous embolization. Recently, there have been reports^69^, ^70^ that favorable outcomes were obtained by TAE using Onyx® (Medtronic, Irvine, CA, USA) a non‐adhesive liquid embolic agent. Concerning sclerotherapy, there is a case series study of ethanol sclerotherapy alone, reporting relatively favorable outcomes.^71^ Infection and bone necrosis are frequent complications of embolization, and they may occur, when an embolic agent, a foreign body, is injected into lesions that were in contact with the external environment due to direct puncture or hemorrhage. Surgical treatments as listed above are performed primarily after endovascular embolization. Invasive radical resection and reconstruction should be avoided at least as the initial treatment, because many lesions can now be controlled by endovascular embolization alone with the advancement of endovascular technique.

As mentioned above, endovascular embolization is performed using various approaches and embolic agents selected depending on the facility and patient. There are also a wide variety of surgical treatment options. Since the treatment may be performed by combining these options, AVM of the maxilla and mandible should be treated at the institutions where multidisciplinary treatment can be performed by experienced physicians.

#### CQ6: What treatments are appropriate for AVM of the fingers?

*Recommendation*: Although embolization or sclerotherapy is effective as it alleviates symptoms, such as pain, sufficient evaluation is necessary because of the risk of finger necrosis and nerve damage. In surgical resection, total resection is recommended, because partial resection is likely to result in enlargement of the lesion. Occasionally, the disorder results in finger amputation.

*Strength of recommendation*: 2 (weak)

*Evidence*: D (very weak)

*Comments*: As a result of primary screening, 38, 16, and 35 papers were retrieved from PubMed, Cochrane Library, and JCRM, respectively. However, during secondary screening, many reports concerned AV shunts in dialysis patients and AVM at sites other than the fingers. Eventually, only 10 papers consisting of 3 case series and 7 case reports remained as references, and the evidence level is extremely low (D: very weak).

AVM of the fingers is often difficult to treat, and treatments are likely to be ineffective, particularly, when the lesion extends from the fingers to the palm. In addition, when AVM is localized in the fingers, complications are likely to occur after treatment.^72^ It is recommended to conduct treatment by a team from several departments including the plastic surgery, vascular surgery, and radiology departments.^73^ 3D‐CTA is useful for preoperative examination.^74^ Since complete cure is difficult to obtain by embolization therapy, it is recommended to be performed for alleviation of symptoms such as pain only in symptomatic areas.^75^ In addition, as there is the possibility of re‐enlargement after embolization, it is recommended to periodically follow‐up the condition and repeatedly perform embolization each time symptoms appear.^73^ Surgical resection is necessary for permanent cure, and total resection is recommended as there is the possibility of re‐enlargement after partial resection.^76^, ^77^, ^78^ Reconstruction is occasionally necessary, but treatment may end in finger amputation. In this event, preoperative embolization or sclerotherapy is effective.^79^ The present review has fallen short of clarifying situations in which preoperative embolization is useful in fingers to which a tourniquet can be applied.

#### CQ7: What treatments are effective for painful VM?

*Recommendation*: Sclerotherapy and surgical resection, as well as conservative treatments, such as compression, oral aspirin, and low‐molecular‐weight heparin, are reported to be effective depending on the site and size of the lesion and symptoms. Endovascular laser treatment, percutaneous cryotherapy, and photodynamic therapy have also been suggested to be effective.

*Strength of recommendation*: 2 (weak)

*Evidence*: D (very weak)

*Comments*: As a result of literature search, 54 reports in English and 4 in Japanese were retrieved by primary screening. Of these reports, 39 in English and 4 in Japanese were extracted by secondary screening. Many options were reported as treatments for pain associated with VM, but all these documents were case series or case reports without comparison of treatments. Therefore, the evidence level was rated as “very weak,” and the recommendation level as “weak.”

Pain is one of the major symptoms of VM. It may respond to conservative treatments that are relatively easy to manage, such as compression and oral aspirin, depending on the site and size of the lesion and symptoms. Particularly, when pain is localized, surgery should also be considered. Relatively novel treatments, such as endovascular laser therapy, percutaneous cryotherapy, and photodynamic therapy, have been reported to be effective for controlling local VM, and they have also been reported to be effective for the control of pain. Limb lesions accompanied by localized intravascular coagulopathy may be indications for low‐molecular‐weight heparin. Reports on various treatments are mentioned below.

1. Compression

Although there has been no report of comparative evaluation, compression is reportedly effective according to reviews by specialized medical facilities.^80^, ^81^, ^82^

- Oral aspirin

The literature is also limited, but the treatment has been mentioned in reviews.^80^, ^81^, ^82^ Nguyen *et al*.^83^ reported that pain was alleviated in 17 (77%) of 22 patients in whom oral aspirin therapy was initiated for pain.

- Sclerotherapy

Sclerotherapy has often been performed using ethanol or polidocanol. The literature concerning other sclerosing agents is scarce, and their effectiveness remains largely unclear.

Each sclerosing agent is commented below.

(i) Ethanol

Shireman *et al*.^84^ reported remission in 6 of 12 patients, and Rimon *et al*.^85^ reported alleviation or remission in 14 patients with painful VM (including 8 with lower limb lesions) except in 4 of those with lower limb lesions. Marrocco‐Trischitta *et al*.^86^ reported that pain was resolved in both (100%) of 2 women with external genital lesions.

Concerning the use of ethanol, Suh *et al*.^87^ reported alleviation to 50% or less of the pre‐treatment state according to a VAS in 12 (71%) of 17 patients who underwent sclerotherapy using its mixture with lipiodol, and Dompmartin *et al*.^88^ reported 37 patients who underwent sclerotherapy using its mixture with ethylcellulose. According to Schumacher *et al*.,^89^ also, 77 patients underwent sclerotherapy using ethylcellulose‐ethanol in a multicenter study, and significant improvement compared with the pre‐treatment state was observed in all patients.

(ii) Polidocanol (including foam sclerotherapy)

Mimura *et al*.^90^ reported remission in 6, alleviation in 4, and no change in 1 of 11 patients with painful VM, and remission in 12 (41%), alleviation in 14 (48%), no change in 2 (7%), and exacerbation in 1 (3%) of 29 patients in another study.^91^ Cabrera *et al*.^92^ treated 50 patients (including 15 with Klippel‐Trenaunay syndrome) using a foamed sclerosing agent and reported remission in 25 (50%) and alleviation in 14 (28%). Marrocco‐Trischitta *et al*.^86^ reported resolution of pain in all 3 women with external genital lesions.

(iii) Ethanolamine oleate

Ozaki *et al*.^93^ reported remission in 2 (20%) and alleviation in 8 (80%) of 10 patients.

(iv) Sodium tetradecyl sulfate

Krokidis *et al*.^94^ reported alleviation of pain in 4 of 5 women with external genital lesions.

- Surgical resection

Enjolras *et al*.^95^ performed surgical resection in 7 of 13 patients with VM involving a wide area including the knee joint and reported alleviation of pain in 5 (71%). Steiner *et al*.^96^ reported alleviation to 50% or less of the pre‐treatment state by a VAS in 24 (89%) of 27 patients with background pain and 12 (92%) of 13 patients with acute episodic pain. In addition, Noel *et al*.^97^ performed surgical resection and compression therapy for VM of the lower extremities in 20 patients with Klippel‐Trenaunay syndrome and reported disappearance of pain in 18 (90%) (mean follow‐up period: 63 months).

- Endovascular laser therapy

Sidhu *et al*.^98^ and Lu *et al*.^99^ reported alleviation of pain in all 8 and 51 lesions in 6 and 33 patients, respectively. Liu *et al*.^100^ also reported marked responses in 46 (35%), responses in 84 (63%), and no change in 3 (2%) of 133 patients.

- Low‐molecular‐weight heparin

According to Mazoyer *et al*.,^101^ only low‐molecular‐weight heparin was effective when VM are complicated by localized intravascular coagulation, resulting in disappearance of pain.

- Percutaneous cryotherapy

Cornelis *et al*.^102^, ^103^ reported remission of pain in a report of 1 case (observation period: 2 months) and a report of 4 cases (observation period: 6 months).

- Photodynamic therapy

Betz *et al*.^104^ reported remission in 2 and alleviation in 1 of 3 patients.

#### CQ8: Is laser therapy effective for VM?

*Recommendation*: With appropriate selection of the type of laser according to the site, size, and symptoms of the lesion, laser therapy can be effective for the treatment of VM. It is recommended to evaluate whether the net benefit by laser therapy is worth the cost and resources by comparison with other treatments such as sclerotherapy and surgical resection.

*Strength of recommendation*: 2 (weak)

*Evidence*: C (weak)

*Comments*: VM is a lesion that has been called cavernous hemangioma, and it causes pain, functional impairment, and cosmetic defect depending on the affected site. In addition to conventional resection of the lesion, sclerotherapy has been widely performed in recent years. While reports of laser therapy for VM have increased, there have not been prospective studies comparing the results of laser therapy with those of surgery or sclerotherapy, among types of laser equipment different in wavelength, or using the same equipment type but changing the irradiation method or parameter setting. We analyzed 134 papers extracted by primary screening and 98 papers extracted by secondary screening. Concerning more than 30 cases, the answer to the CQ was based primarily on 7 reports summarizing the methods and sites of treatment and benefits and harms derived from treatment (decrease in size of the lesion, alleviation of symptoms, complications).

In the facial skin, pigmentation and scar formation after irradiation can be serious treatment‐related complications compared with unexposed areas. In the airway and digestive tract, the mass effect of the lesion and chronic bleeding from the lesion can be causes of serious symptoms. Thus, as the goal to achieve varies with the anatomical site of the lesion, we reviewed the literature by the anatomical site (the neurosurgery field was excluded). For this reason, we also extracted benefits and harms of treatment from the text of the reports of less than 30 cases. The relevant departments surveyed included ENT, dental and oral surgery, gastrointestinal surgery, ophthalmology, plastic surgery, and dermatology, and secondary screening overviewed laser therapy for VM and vasodilatory lesions.

**Figure 1:**
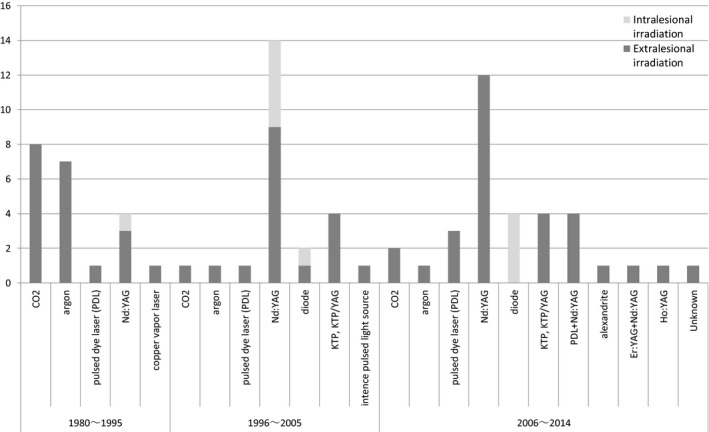
Reports of laser therapy for hemangiomas/vascular malformations (primarily venous) in various periods and types of laser used in the reports (CQ8). *Notes*: CO2; 10 of 11 reports recommend the use for surgical resection. Argon; There are 4 reports of mixed treatments for CM and VM. Nd:YAG; There are 12 reports of its use in combination therapy with surgery, sclerotherapy, or other lasers. Alexandrite; There is only 1 report summarizing cases treated with alexandrite in combination therapy with other lasers.

When a new laser instrument is developed and put into use, reports of therapeutic results using the equipment are presented. The types of laser used for treatment varied widely. The types of laser that have been reported are summarized chronologically in a graph (Fig. 1). While the type of laser with more reports is not necessarily more effective, the graph is considered to reflect tendencies of laser types that are established and gain favorable appraisal or no longer in use.

Since dye laser used for the treatment of port wine stain (wavelength: 595 nm) uses hemoglobin as the observer/heater, photothermal conversion occurs efficiently in the blood vessel, and the thermal energy reaches endothelial cells.^105^ However, its optical penetration depth is shallow, being about 1 mm in both the skin and mucosa.^105^ However, in Nd:YAG laser with a longer wavelength (1,064 nm), the optical penetration depth is about 3 mm in the skin and about 6 mm in the mucosa.^105^ Although Nd:YAG laser is advantageous compared with dye laser for the treatment of deep lesions, heat is generated also in perivascular tissues, because light is converted to heat as it is absorbed by water contained in the skin and mucosa.

The target of laser treatment for VM is the endothelium of morbidly dilated blood vessels. There is no light that is specifically absorbed by endothelial cells and emits heat. Satisfactory therapeutic results cannot be expected unless treatment is performed by selecting the laser type and modifying the irradiation method based on the understanding of such principles and limitations of phototherapy.

Concerning small VM of the mucosa, tongue, lips, and glans penis, in which scar formation after treatment poses no serious problem, there are a number of reports^106^, ^107^, ^108^ that lesions were resolved by treatment using Nd:YAG laser. There have also been cases in which favorable results were obtained by treatment of anemia due to gastrointestinal bleeding^109^ and of symptoms, such as airway obstruction, due to the mass effect of the lesion.^110^ While transient purpura and swelling after treatment are unavoidable, they often resolve rapidly.^99^ Modifications of the irradiation setting and method are necessary to obtain satisfactory results and avoid serious complications, such as peroneal neuropathy^100^ and pigmentation and scar formation of the facial skin,^106^, ^108^ and we must learn from the experience of experts.

Nd:YAG laser irradiation by insertion into the lesion under ultrasound guidance has begun to be performed as a treatment to avoid damage of important organs and nerves,^98^, ^99^, ^100^ therapeutic experience using this technique has been accumulated, and detailed records and reports have been presented. At present, the results have been satisfactory in terms of safety and efficacy, and standardization of the procedure is anticipated.

#### CQ9: Is sclerotherapy effective for VM?

*Recommendation*: Sclerotherapy for VM is effective for alleviating symptoms and reducing the size of the lesion and is recommended.

*Strength of recommendation*: 2 (weak)

*Evidence*: D (very weak)

*Comments*: VM is lesion that used to be called cavernous hemangioma or intramuscular hemangioma and differ from infantile hemangioma. VM poses problems, such as pain, swelling, and functional impairment, and has been treated conventionally by surgical resection. In Western countries, percutaneous sclerotherapy has a long history. In 1989, Yakes *et al*.^111^ reported ethanol sclerotherapy for VM, and the treatment has since been performed worldwide. Recently, sclerotherapy, which is mildly invasive, permits functional and morphological preservation, and can be performed repeatedly, is widely used. However, as of 2016, sclerotherapy is not covered by medical insurance in Japan. In addition, there has been no randomized controlled trial (RCT) on the usefulness of sclerotherapy for VM compared with surgery or placebo.

As a result of secondary screening, 76, 3, and 3 papers were extracted from PubMed, Cochrane, and JCRM, respectively. They include 3 semi‐RCTs, but randomization and blinding were insufficient, and their quality as an RCT was low. Also, the theme evaluated by all these RCTs was “comparison of sclerosing agents in sclerotherapy,” and none compared sclerotherapy with other treatments. Therefore, control groups related to this CQ were not established, and their contribution as a whole is weak. The other literature was all case reports or case series, and the evidence level is D (very weak). As mentioned above, while the evidence level is low, most of the studies reported alleviation of symptoms and regression of lesions in a high percentage (70–90%) of the patients, suggesting the usefulness of sclerotherapy.

The sclerosing agents used included absolute ethanol, polidocanol, ethanolamine oleate, sodium tetradecyl sulfate (STS), and bleomycin. Polidocanol is approved as a sclerosing agent for lower limb varices and esophageal varices, and ethanolamine oleate as a sclerosing agent for esophageal varices. STS is not marketed in Japan. Each sclerosing agent has characteristic complications. Recently, injection of polidocanol, STS, as a foam by mixing with CO_2_ or air has been increasingly performed. Sclerotherapy using ethanol is often performed under general anesthesia, but sclerotherapy using polidocanol or ethanolamine oleate can be performed under local anesthesia.

Three RCTs have been reported as studies that evaluated differences in therapeutic effect according to the sclerosing agent. However, randomization and blinding are insufficient, and their quality as an RCT is low. In addition, the theme evaluated in these RCTs was “comparison of sclerosing agents in sclerotherapy” rather than comparison with other treatments.

Although the evidence level is low, there have been a few case series that reported the usefulness of sclerotherapy, and a wide variety of sclerosing agents including ethanol, polidocanol, ethanolamine oleate, STS, and bleomycin were used. Among studies with a relatively large number of patients, there is a report^112^ that sclerotherapy using ethanol in 87 patients with craniofacial VM resulted in a ≥75% decrease in size in 23 (32%) and a 25–75% decrease in size in 37 (52%). The results of sclerotherapy using polidocanol in 50 patients with VM were excellent in 19, good in 16, moderate improvement in 13, and unchanged or worse in 2.^92^ The results of sclerotherapy using ethanolamine oleate performed in 83 patients, who were mostly children, were complete remission of symptoms in 79 lesions and significant alleviation in 6 lesions.^113^ Sclerotherapy using STS resulted in subjective improvements in 174 (85.3%) of 204 patients.^114^ The results of sclerotherapy using bleomycin were complete cure in 185 of 260 patients, marked improvement in 44, and some improvement or no change in 31.^115^ In addition, regarding the size of the lesion, a very satisfactory decrease was achieved in 104, and a satisfactory decrease was achieved in 10, of 120 patients.^116^

Papers that evaluated the types of VM that are likely to respond to sclerotherapy include those by Goyal *et al*.,^117^ Yun *et al*.,^118^ Mimura *et al*.,^91^ Rautio *et al*.,^119^ Lee *et al*.,^112^ Yamaki *et al*.,^120^ and Nagao *et al*.^121^ Types of lesions that were likely to be sclerosed were reported to be well‐defined small (≤5 cm) lesions by Goyal *et al*.,^117^ females, lesions showing no or delayed delineation of the draining vein, and lesions well‐defined on MRI by Yun *et al*.,^118^ small lesions, well‐defined lesions, and lesions that show prolonged drug retention by Mimura *et al*.,^91^ localized lesions by Lee *et al*.^112^ and Yamaki *et al*.,^120^ and slow flow type lesions by Nagao *et al*.^121^ Nomura *et al*.^122^ evaluated the therapeutic effect according to the degrees of functional and gross improvements and reported that the therapeutic effect was greater in head and neck and trunk lesions than in the upper or lower limb lesions. Moreover, Rautio *et al*.^119^ reported that the treatment‐related improvement in the quality of life was higher when the lesion did not involve the muscle or was ≤5 cm in size.

Various complications ranging from mild complications, such as transient neuropathy and local inflammation, to serious ones, such as myopathy, skin necrosis, and deep venous thrombosis/pulmonary embolism, have been reported. In sclerotherapy using ethanol or polidocanol, particularly serious life‐threatening complications have been reported. Qiu *et al*.^123^ reviewed the literature concerning sclerotherapy for VM and reported that shock and pulmonary embolism occurred in 0.19% each of 522 patients who underwent sclerotherapy using ethanol and that ethanol was used at 1 mL/kg in those who developed shock. They also reported that a decrease in blood pressure/bradycardia was noted in 0.61% of 163 patients who underwent sclerotherapy using polidocanol but that its differentiation from vagus nerve reflex was clinically difficult. Wong *et al*.^124^ reported a case of shock after sclerotherapy using 0.86 g/kg ethanol but could be saved. Tachibana *et al*.^125^ reported that 2 patients (1.1%) developed pulmonary embolism and that the amounts of ethanol used were 0.71 and 0.16 mL/kg. Concerning sclerotherapy using polidocanol, also, cardiac arrest in children has been reported by authors including Marrocco‐Trischitta *et al*.^126^ and Shimo *et al*.,^127^ who used 4 mL of 1% polidocanol (body weight: 20 kg) and 10 mL of 3% polidocanol (15.6 kg), respectively.

In conclusion, sclerotherapy is generally considered effective for VM, but its problems are that the evidence level is low and that the procedure has not been standardized. In addition, serious complications that are rare but life‐threatening have been reported, and caution is needed in deciding the dose of the sclerosing agent.

#### CQ10: Are clotting abnormalities due to VM an indication for radiotherapy?

*Recommendation*: Radiotherapy should not be performed without careful evaluation because malignant neoplasm, growth disorders, and functional impairment have been reported as late complications.

Many reports included both VM and vascular tumors in the subjects, which make it difficult to assess the therapeutic effects of radiotherapy.

*Strength of recommendation*: 2 (weak)

*Evidence*: D (very weak)

*Comments*: As a result of primary screening, 6 and 2 documents were retrieved from PubMed and JCRM. However, as a result of secondary screening, liver hemangioma was excluded, and 10 papers including the references from the previous guideline were reviewed. The reviewed papers were case series or case reports, and the evidence level of the literature as a whole is D “very weak.”

While there have been reports that radiotherapy was performed for the treatment of vascular tumors and vascular malformations, it is difficult to judge whether the treatment was performed by determining the disorders.

According to many reports,^128^, ^129^, ^130^, ^131^, ^132^ radiotherapy has been performed to treat Kasabach‐Merritt phenomenon. However, while there is no mention about Kasabach‐Merritt phenomenon, there is a report^133^ of 5 cases in which giant hemangiomas accompanied by clotting disorders, thrombocytopenia, heart failure, and bleeding were controlled by multidisciplinary treatment including radiotherapy.

Vascular tumors that cause Kasabach‐Merritt phenomenon are considered to be kaposiform hemangioendothelioma or tufted angioma rather than infantile hemangioma.^134^ Because VM and infantile hemangiomas are included in other vascular tumors in the lesions described in these reports, they are not considered to be indications of radiotherapy for VM or infantile hemangiomas.

Schild *et al*.^128^ reported 13 cases of symptomatic hemangioma (11 of which were pathologically diagnosed as cavernous hemangioma, but as the report is old, vascular tumors and vascular malformations were not distinguished and were probably included). Radiotherapy at 6.25–40 Gy was carried out in these 13 cases. The lesions were located in the limbs in 5, face in 2, vertebral bodies in 3, pituitary fossa in 1, sacrum in 1, and bladder in 1. Note that organs that should be excluded in this CQ were included.

Of these patients, 2 (1 each with a limb and facial lesion) exhibited Kasabach‐Merritt phenomenon and showed normalization of clotting disorder (evaluated according to the platelet count and fibrinogen level) after treatment. However, they were aged 3 years and 5 months, and the lesions may not have been VM.

When the subjects were limited to patients with limb or facial lesions, CR was observed in 2, PR in 4, and no response in 1 in terms of decrease in the lesion size, and CR was observed in 4, PR in 1, and no response in 2 in terms of the control of symptoms.

A serious treatment‐related complication, which was unilateral visual impairment, was noted in 1 (14 Gy/8 fr).^128^ These problems have been recognized as late complications of radiotherapy for vascular tumors or vascular malformations; malignant neoplasms, such as breast cancer,^135^ thyroid cancer,^136^ and vascular sarcoma,^137^ visual impairment mentioned above,^128^ shortening of the lower limb, and restriction of the joint motion range.^131^

According to Coldwell *et al*.,^137^ late complications of radiotherapy for hemangiomas in infancy include bruise and Stewart‐Treves syndrome after the patients reach adulthood. Angiosarcoma is also observed. They reported that the median survival period was 24 months, and the 5‐year survival rate was about 10%, in those who developed angiosarcoma.

As observed above, the diagnosis was not confirmed in the reports that have suggested the effectiveness of radiotherapy, and its indications have not been specified. In addition, there have been a considerable number of reports of late complications due to radiotherapy. Thus radiotherapy should not be performed without careful evaluation.

#### CQ11: Is there difference in the effectiveness of dye laser treatment for CM according to the site of the body?

*Recommendation*: Dye laser treatment for CM is likely more effective in the face and neck region compared with other sites, and it is more likely to cause complications such as pigmentation in the limbs.

*Strength of recommendation*: 2 (weak)

*Evidence*: C (weak)

*Comments*: As a result of literature searches, 176 papers consisting of 139 from PubMed and 37 from JCRM were extracted. They included a few reports that were allegedly RCTs but were not actual RCTs. Therefore, a total of 26 papers consisting of 15 from PubMed and 11 from JCRM including case series with a large number (≥100) of relevant cases were selected by secondary screening. In addition, a total of 17 papers were adopted as references for the comments in the guidelines by adding 3 papers in English extracted by manual search to 6 from PubMed and 8 from JCRM considered to be relevant or closely related to the CQ among those selected by secondary screening. Since there was no RCT, the evidence as a whole was rated as C (weak).

Concerning the effect of dye laser treatment for CM, most of the reports were about the effects for hemangioma simplex or port‐wine hemangioma in Japan and port‐wine stain abroad.

There have been a few papers^138^, ^139^, ^140^, ^141^, ^142^, ^143^, ^144^, ^145^, ^146^, ^147^, ^148^, ^149^, ^150^, ^151^, ^152^ that evaluated the therapeutic results of dye laser treatment according to the site in a small to relatively large number of patients. The laser equipment used varies from early dye laser to pulsed dye laser with adjustable pulse duration with a cooling system, and reports limited to variable‐pulse pulsed‐dye laser with a cooling system, which is widely used today, are extremely few.

According to many reports,^138^, ^139^, ^140^, ^141^, ^142^, ^143^, ^144^, ^145^, ^146^, ^147^, ^148^, ^149^ the response rate is higher in the face and neck region than in the trunk and limbs. In the face, it has been reported that the response rate is higher in the palpebral, forehead and temporal, and lateral buccal regions but is significantly lower in the territory of the 2nd division of the trigeminal nerve (dermatome V2), and that the number of irradiations tends to increase in the midline region, frequently resulting in persistence of redness.^150^ There is a report^151^ that the response rate did not differ significantly among regions in the lower limb. While the number of patients was small, it has been reported that treatment of the foot involves stronger pain but was less effective than in the face but that the degree of patient satisfaction was relatively high.^152^

The incidence of complications of dye laser (bleb formation, depigmentation, pigmentation, scar formation) is reported to be low, being 1.7% in adults, 0.6% in children, and about 1.4% in all patients even when all sites of the body are included, and no significant difference has been reported in the age at the beginning of treatment, Fitzpatrick skin type,^153^ site, number of treatments, or irradiation energy between those who developed complications and those who did not, but complications may occur more frequently in the lower limbs.^154^ Moreover, there is a report^152^ that complications, such as pigmentation, depigmentation, and atrophic scar, were observed more frequently in the lower limbs.

#### CQ12: Do CM recur after dye laser treatment?

*Recommendation*: Although the effectiveness of dye laser treatment for CM is established, the recurrence rate may increase with time after treatment.

*Strength of recommendation*: 2 (weak)

*Evidence*: C (weak)

*Comments*: As a result of literature searches, a total of 211 papers consisting of 149 from PubMed, 53 from Cochrane, and 9 from JCRM were retrieved. They did not include RCTs, and a total of 30 papers consisting of 23 from PubMed and 7 from Cochrane, which were mostly case reports and case series studies, were extracted by secondary screening. In addition, a total of 10 papers that were relevant and closely related to the CQ (including 8 case series) consisting of 7 from PubMed, 2 from Cochrane, and 1 in English retrieved by manual search were adopted as references for the guidelines. Since there was no RCT, the strength of evidence of the group of literature concerning this CQ is C “weak”.

Concerning papers that referred to “whether CM recur after dye laser treatment,” there are 4 retrospective studies^155^, ^156^, ^157^, ^158^ after treatment by pulsed dye laser (wavelength: 585 nm) with a cooling system, and the recurrence rate was 15.9–35%. Also, there is a report^155^ that the recurrence rate increased with time after treatment and was 3.1% after 1 year, 20.8% after 2 years, 40% after 3 years, and 50% after 4 years. Therefore, it is necessary to treat CM with the recurrence after dye laser treatment in mind.

It is difficult to strictly distinguish whether the recurrence is generation of new dilated vessels after laser therapy or it is regeneration of blood vessels damaged due to treatment or re‐proliferation of remaining vessels. However, there have been reports that, in an experiment using mice, angiogenesis occurred in the process of wound healing at the site of irradiation in early recurrence^159^ and that, in an experiment using hamsters, complete treatment was difficult, and morbid vessels persisted, because coagulation was difficult to induce by dye laser irradiation in vessels ≤2–16 μm in diameter.^160^ While there is a report^161^ that genes affected by dye laser therapy early after treatment were identified, further evaluation is necessary to clarify their relationships with the recurrence.

Concerning the prevention of recurrence, there is a report^162^ that the recurrence‐free period was long in the patients treated with a variable‐pulse pulsed‐dye laser with a cooling system (wavelength: 595 nm), which is widely used today, and they were treated within 6 months after birth. In addition, there have been reports of animal experiments using Rapamycin, which inhibits angiogenesis after laser irradiation,^159^, ^163^ and of prospective RCT using imiquimod,^164^, ^165^ and these treatments were considered effective for the prevention of recurrence. However, careful evaluation by large‐scale investigations, including the assessment of the safety concerning drugs, is considered necessary.

#### CQ13: Is dye laser irradiation for CM more effective as it is initiated at a younger age?

*Recommendation*: Laser therapy before the age of 1 year may be effective, and the earliest possible initiation of treatment is recommended as an option.

*Strength of recommendation*: 2 (weak)

*Evidence*: D (very weak)

*Comments*: Concerning the timing of treatment for CM, there is the opinion that early initiation of treatment is recommended, because, in young children, the skin is thinner, so the depth of penetration is larger, the vascular wall is also immature, cure after laser irradiation is better, pigmentation is less, and the irradiation area is small, so the treatment efficiency is higher. However, there is still controversy. As a result of secondary screening of past reports, 6 and 1 were extracted from PubMed and JCRM, respectively. While the papers selected by these screening procedures include 2 papers on prospective studies as described below, their conclusions differed, and the evidence level is considered to decline when these references are reviewed together.

Oguri *et al*.^138^ performed a non‐randomized controlled trial by dividing children into those aged 0–12, 13–24, and 25–36 months and observed significant differences in the response rate combining “markedly effective” and “effective” among the groups. They also compared the response rate according to the age in months at the beginning of treatment in the 0‐year‐old group and reported that the response rate was higher as the treatment was initiated earlier. Furthermore, Nguyen *et al*.^166^ divided their patients into those aged less than 1 year, those aged 1–6 years, and those aged 6 or more years and investigated the correlation between treatment response and age. They reported that those aged less than 1 year and lesions with a size of <20 cm^2^ located in the center of the face showed the best treatment response.

Among reports suggesting no difference in the therapeutic effect according to the age at the beginning of treatment, van der Horst *et al*.^167^ studied 100 patients with untreated CM of the head and neck region prospectively and concluded from the results of colorimetry and clinical evaluation that there was no significant difference in the therapeutic effect of pulsed dye laser among the 4 groups in which the treatment was started at the age of 0–5, 6–11, 12–17, and 18–31 years. In the retrospective study of Katugampola *et al*.,^140^ also, comparison of 4 groups in which treatment was started at the age of 0–5, 6–12, 13–50, and 50+ years showed no significant difference in the therapeutic effect.

Among the above reports, those did not affirm the usefulness of early laser treatment were relatively old. Also, reports of Oguri *et al*.^138^ and Nguyen *et al*.^166^ indicated laser therapy may be more effective in those aged <1 year. In addition, the effectiveness of laser clearly declines when the lesion is elevated or thickens with time. In consideration of “benefits” of early laser treatment and “harms,” which include the occasional necessity of general anesthesia for laser treatment around the eye in small children, the recommendation level was rated as 2D based on the consensus of this guidelines drafting committee.

#### CQ14: Is propranolol safe and effective for infantile hemangiomas?

*Recommendation*: If administered under careful monitoring, oral propranolol therapy may be the first choice for the treatment of infantile hemangioma.

*Strength of recommendation*: 1 (strong)

*Evidence*: A (strong)

*Comments*:

(1) Effectiveness: There was the serendipity that regression of hemangioma was induced in a child under steroid therapy with a giant infantile hemangioma by propranolol administered for obstructive hypertrophic cardiomyopathy in 2008.^168^ Based on this report, oral propranolol therapy began to be used for the treatment of infantile hemangioma, and its high efficacy against alarming hemangioma/life‐threatening hemangioma in the proliferating phase and in patients with cosmetic problems, such as giant lesions in the face, those with ulcerated and hemorrhagic lesions, and those who may develop functional impairment, has been demonstrated, resulting in its use (Hemangiol®, Pierre Fabre Dermatologie, Boulogne, France) as the first choice in Western countries. In addition, its effectiveness for the treatment of hemangiomas after the proliferating phase was also described. Moreover, a group of physicians used propranolol earlier due to cosmetic significance and at the request of the family even in cases of small or localized lesions, and it is also effective in such cases.

A total of 131 papers consisting of 25 from JCRM, 106 from PubMed, and 0 from Cochrane Library were extracted as related to the CQ, “Is propranolol safe and effective for infantile hemangioma?,” and they were subjected to primary and secondary screenings with reduction of hemangioma (effectiveness of propranolol) and treatment‐related complications (adverse effects) as outcomes. Twenty‐six papers,^169^, ^170^, ^171^, ^172^, ^173^, ^174^, ^175^, ^176^, ^177^, ^178^, ^179^, ^180^, ^181^, ^182^, ^183^, ^184^, ^185^, ^186^, ^187^, ^188^, ^189^, ^190^, ^191^, ^192^, ^193^, ^194^ most of which were RCTs or observational studies, were adopted.

For example, Hogeling *et al*.^180^ administered placebo or propranolol at 2 mg/kg/day for 6 months with randomization to 40 patients aged 9 weeks to 5 years with infantile hemangiomas in the face or sites with the potential for disfigurement. They reported significant improvements in size, redness, and elevation in the propranolol group. Elevated lesions disappeared in 4 of the 19 patients in the propranolol group but none of the 18 patients in the placebo group. As for adverse events, the trial was interrupted in 1 patient due to upper respiratory tract infection, and conditions including bronchiolitis, gastroenteritis, streptococcal infection, cool extremities, dental caries, and sleep disturbance were observed.

Zaher *et al*.^181^ observed 45 patients by randomly dividing them into 15 each treated by oral administration, topical application, and intralesional injection of propranolol. Responses were observed in 60% in the oral group, 20% in the topical ointment group, and 13.3% in the injection group. No major adverse events were noted, and the trial was discontinued in 1 in the oral group and 3 in the injection group due to inconvenience or pain of the treatment.

Malik *et al*.^182^ randomly allotted 30 patients aged 1 week to 8 months to propranolol alone, prednisolone alone, or both propranolol and prednisolone. They found that the mean initial response times were lower in the propranolol group than in the prednisolone group but that there was no clear difference between the propranolol + prednisolone group and propranolol alone group. All 10 patients in the propranolol group and 9 patients in the corticosteroid group responded to the 3‐month treatment. However, adverse events were observed in 2 of the 10 patients in the propranolol group (asymptomatic hypoglycemia, insomnia) and 9 of the 10 patients in the steroid group (cushingoid appearance, gastrointestinal upset), and were more frequently in the latter group.

Bauman *et al*.^183^ performed a phase 2, investigator‐blinded, multi‐center RCT in 44 patients aged 2 weeks to 6 months. Propranolol or prednisolone (2 mg/kg/day) was administered orally until halted due to toxic effects or clinical response. During 4‐months treatment, no significant difference was observed between the two groups, for example, with regression of 5 of the 6 tumors in the corticosteroid group and 9 of the 10 tumors in the propranolol group. For long‐term analyses, the effect of prednisolone appeared earlier. While the incidence of adverse events as a whole did not differ between the two groups, severe adverse events were observed in 1 of the 11 patients in the propranolol group but 5 of the 7 patients in the prednisolone group, being significantly more frequently in the latter group.

Léauté‐Labrèze *et al*.^184^ carried out an RCT in patients aged less than 4 months by comparing 7 administered and 7 not administered propranolol. Since color changes and softening were observed within 24 h, and the thickness and size of the lesions decreased within 4 weeks in the propranolol group, the treatment was considered useful for the prevention of scarring. No serious adverse effect was observed except asymptomatic mild decreases in heart rate and diastolic blood pressure.

There have also been comparisons between atenolol and propranolol and between laser and laser plus topical propranolol.^185^, ^186^ In 2015, the largest RCT was published in the New England Journal of Medicine, also reporting that propranolol was significantly effective for hemangioma compared with placebo.^187^ Hemangioma showed complete or nearly complete resolution after 6‐month treatment in 2 (4%) of 55 patients in the placebo group and 61 (60%) of 101 patients in the 3 mg/kg/day propranolol group.

Furthermore, there have also been a few systematic reviews and meta‐analyses primarily of observational studies. Menezes *et al*.^188^ reviewed 49 English papers published between June 2008 and September 2010, and summarized 6 studies with 10 or more patients administered propranolol (totally 154 patients). Propranolol was administered to infants with a mean age of 4.5 months at a dose of 2 mg/kg/day in 65% and 3 mg/kg/day in 25.3%. Two‐thirds of the patients were treated with propranolol alone. Recurrence was observed in 21% after treatment for a mean of 4.3 months, and adverse events including hypotension, somnolence, wheezing, insomnia, agitation, nightmare, cool hands, night sweat, gastroesophageal reflux disease, and psoriasiform rash appeared in 18.1%.

Marqueling *et al*.^189^ reviewed the therapeutic results in 1,264 patients (including 806 girls) in 41 reports published from 2008 to 2012 retrieved from Medline and Cochrane database. The treatment was initiated at a mean age of 6.6 months at 2.1 mg/kg/day and continued for a mean of 6.4 months. The overall response rate was 98%, and the treatment was also effective in clinically problematic areas such as the face (100%), airway (100%), periorbital (98%), head and neck region (97%), and parotid gland (82%). However, recurrence was observed in 17% after treatment. Adverse effects were noted in 371 of 1,189 patients. Changes in sleep (136 patients) and acrocyanosis (61) were the most frequent among them, and hypotension was observed in 44, bradycardia in 9, and hypoglycemia in 4 as serious complications. In conclusion, the grade of recommendation was 1, quality of evidence is A, and propranolol was recommended as the first‐line drug for complicated infantile hemangiomas. Regarding adverse effects, the grade of recommendation was 1, quality of evidence was A or B. while serious adverse effects may be observed, their frequency is low, and they can be usually avoided by proper monitoring at initiation of treatment.

Xu *et al*.,^190^ on the other hand, evaluated volume changes, improvement in overall appearance, visual function, and adverse effects using 15 online databases. The data of 419 cases were analyzed, but meta‐analysis was not performed because of the wide differences among studies. Some studies showed superiority of propranolol compared with corticosteroid in reducing volume and improving the overall appearance. No marked difference was noted in adverse effects or visual function.

In addition, in meta‐analysis of 16 studies (2,629 cases) and 25 studies (795) published in 1965–2012, 69% of the patients responded to 12‐month corticosteroid therapy, but the response rate to propranolol was 97% with a significant difference.^191^

In periorbital hemangiomas, the response rate to propranolol was found to be significantly higher than that to corticosteroid by meta‐analysis of papers published before 2013,^192^ and propranolol showed the strongest effect against airway hemangiomas compared with steroid, CO_2_ laser, and vincristine on meta‐analysis.^193^, ^194^

From these observations, we concluded that propranolol was significantly more effective than placebo and to be similarly effective compared with corticosteroid. Concerning the safety, propranolol is considered to have significantly fewer adverse effects than corticosteroid. Since there have been several RCTs and systematic reviews or meta‐analyses directly related to this CQ, the evidence level is considered to be extremely high.

(2) Meta‐analysis: Regarding the effectiveness and adverse effects of propranolol, a large number of systematic reviews and meta‐analyses based on observational studies are already present in the above 26 papers. We, therefore, used only 4 reports^180^, ^182^, ^183^, ^187^ on interventional studies for meta‐analysis.

As a result of meta‐analysis, regarding “tumor reduction,” it was found that propranolol had significantly stronger reducing effects than placebo and that it had a stronger reducing effect, which, however, was not significant, compared with corticosteroid. Concerning “complications,” propranolol was compared with steroid and was shown by 2 RCTs to have significantly fewer adverse events than corticosteroid. Since this meta‐analysis found significantly stronger reducing effect of propranolol compared with placebo and in fewer complications compared with steroid, and since our results were similar to those of systematic reviews of many existing observational studies considered to have high‐quality evidence, we considered that there was a major tendency in this CQ and judged the evidence level as A.

(3) Estimated action mechanism: Beta‐blockers have a wide range of actions on the blood vessels and vascular endothelium, and have diverse actions on cell proliferation and vascular remodeling. Thus, the action mechanism of propranolol on infantile hemangiomas is still unclear. In vascular endothelial cells, propranolol is considered to induce vascular contraction by suppressing NO production, inhibit renin production, control angiogenesis by regulating the expression of VEGF•bFGF•MMP2/MMP9, and induce apoptosis, but it may also affect pericytes and hemangioma stem cells.^195^, ^196^, ^197^

(4) Adverse events associated with propranolol in children

In conducting propranolol therapy, it is necessary to have knowledge about possible adverse effects, their symptoms, and their management. In addition, as there are also preventive measures for, and points of attention about, adverse effects and the timing for discontinuation of propranolol, sufficient explanation to the patients and their families is essential.

Adverse events that have been reported include sleep disorders, peripheral cyanosis, hypotension (symptomatic, asymptomatic), bradycardia (symptomatic, asymptomatic), hypoglycemia, respiratory disorders, gastrointestinal disorders, and mental disorders. Severe cases that require interruption of treatment are few, but particular caution is needed regarding the following points.^189^, ^196^, ^197^, ^198^, ^199^, ^200^

(a) Since there is the risk of hypoglycemia, the patient should be fed before and after propranolol administration. If the patient cannot be fed, or is vomiting, for some reason, the administration should be suspended.

(b) Since propranolol has cardiovascular adverse effects, such as hypotension and bradycardia, interviewing for the past history and familial history, examination, and electrocardiography are recommended before treatment. Even if no abnormality is noted on these examinations, hypotension, and bradycardia may occur during treatment. In such cases, interruption of the administration is necessary.

(c) Propranolol is contraindicated for bronchial asthma as it causes bronchial contraction due to its β2‐blocking action. Caution is also necessary in patients who have been suspected to have bronchial asthma.

#### CQ15: What treatments are effective for ulcer formation in infantile hemangioma?

1. Propranolol

*Recommendation*: The administration of propranolol is recommended for ulcer formation.

*Strength of recommendation*: 2 (weak)

*Evidence*: C (weak)

- Topical administration of antibiotics

*Recommendation*: Topical and systemic administration of antibiotics is recommended for ulcer formation.

*Strength of recommendation*: 2 (weak)

*Evidence*: D (very weak)

- Dressings

*Recommendation*: The use of dressings is recommended for ulcer formation.

*Strength of recommendation*: 2 (weak)

*Evidence*: D (very weak)

- Laser therapy

*Recommendation*: Although laser therapy may be effective in some patients with ulcer formation, the evidence is not considered sufficient at present.

*Strength of recommendation*: 2 (weak)

*Evidence*: D (very weak)

- Systemic administration of steroid

*Recommendation*: Systemic administration of steroid is recommended not to be performed for ulcer formation.

*Strength of recommendation*: 2 (weak)

*Evidence*: D (very weak)

- Platelet‐derived growth factor preparations

*Recommendation*: The accumulation of cases is insufficient for the judgment of the recommendability of the use of platelet‐derived growth factor preparations for ulcer formation.

*Strength of recommendation*: No recommendation

*Evidence*: D (very weak)

*Comments*: Concerning this CQ, 42 papers in Japanese and 156 in English were retrieved. As a result of their primary screening, 47 papers were submitted to secondary screening for this CQ. None of them were about studies with a high level of evidence, such as RCT, and they were all retrospective studies, case series, or case reports.

As a result, 15 papers in English were adopted, and the evidence level was C for propranolol alone, because of the presence of a prospective controlled trial, but D for other treatments, because the related papers were case reports or case series.

According to cross‐sectional analysis in a multicenter prospective cohort study in 1,096 cases of infantile hemangioma by Chamlin *et al*.,^201^ it was complicated by ulcer, which was or was not bleeding, in 173 (15.8%), the median age of the patients was 4.0 months (SD = 8.5, mean = 6.6 months), and the age at the first examination was significantly lower in patients with ulcerated hemangioma (median = 3.5 months, mean = 3.98 months) than in those with non‐ulcerated hemangioma.

By the site, ulcer formation was observed in 21 (30%) of 71 patients in the lower lip, 25 (25%) of 100 patients in the neck, and 46 (50%) of 93 patients in the perianal/perigenital area, and the frequency was statistically lowest in the upper eyelid (*P* = 0.0140).

Ulcer formation was observed more frequently in mixed or segmental hemangiomas. Bleeding was noted in 78 lesions (41%) and was mild in 56 (29%), moderate in 11 (6%), and severe in 4 (2%). Severe bleeding occurred in 3 lesions in the limbs and 1 lesion in the face, and bleeding occurred in 2 cases at home. Two cases required blood transfusion by hospitalization, because they showed symptoms due to serious bleeding. Of the ulcerated hemangiomas, 67 (35%) were in the proliferating phase.

Ulcerated hemangiomas required treatment (odds ratio [OR] = 6.86, 95% CI = 3.70–12.71, *P* < 0.0001), and non‐ulcerated hemangiomas were observed (OR = 19.01, 95% CI = 11.23–28.88, *P* < 0.0001). Ulcerated hemangiomas tended to be treated by conventional wound care and pulsed dye lase (OR = 2.03, 95% CI = 1.19–3.46, *P* < 0.0091), and non‐ulcerated hemangiomas were treated by topical glucocorticoid administration (OR = 2.57, 95% CI = 1.49–4.43, *P* < 0.0007) and surgical resection (OR = 2.04, 95% CI = 1.08–3.86, *P* < 0.0286).

However, propranolol has recently been suggested to be effective regardless of the presence or absence of ulcer formation, and as it has few adverse effects, it is expected to become the first choice treatment in the future.

[Treatments]

1. Oral propranolol

Hermans *et al*.^172^ treated 20 previously treated patients with ulcerated infantile hemangioma using propranolol and compared them with 36 patients treated without propranolol. The administration was initiated by hospitalization, and the dose was increased from 0.7–1.0 to 2.0–2.5 mg/kg/day in 3 divided doses at an interval of at least 3 days. The blood pressure, heart rate, and blood sugar level were monitored during the initial administration period, and the administration was continued on an outpatient basis until the age of 1 year. The mean age at the beginning of propranolol administration was 3.5 months, and the mean duration of administration was 9.1 months. Not only the color and elevation of the lesion but also pain was reduced from early after the beginning of administration. The administration was concluded before the age of 1 year in 19 patients, and no recurrence of ulcer was noted in any of these patients except that some reactivation (enlargement) of hemangioma was observed after the discontinuation in 4 of these patients.

The mean time until complete cure of ulcer was 8.7 weeks, and those in whom the administration was initiated later (>3.5 months) tended to require a longer time until cure than those in whom the administration was initiated earlier (*P* = 0.025). Also, analysis using the *t*‐test showed a significant difference in the time until disappearance of the tumor, which was 8.7 and 22.4 weeks (*t* = 2.6, d.f. = 38, *P* = 0.012, 95% CI = 3.2–24.2) in the treated and control groups, respectively. Temporary sleepiness/malaise was observed in 6 patients, irritable before falling asleep in 2 patients, coldness of the limbs in 6 patients, anorexia in 2 patients, and gastrointestinal disorders (diarrhea, vomiting) in 1 patient, but no adverse event was noted in 9 patients.

Vercellino *et al*.,^202^ who started the administration at 1 mg/kg/day and increased to 2 mg/kg/day, and Sadykov *et al*.,^203^ who started the administration at 2 mg/kg/day, also reported that propranolol was effective.

- Topical and/or systemic administration of antibiotics

Kim *et al*.^204^ topically administered antibiotics in 40 patients with ulcerated hemangioma and reported that the results were better in 37 patients (92.5%), worse in none, and no change in 3 patients (7.5%). They also systemically administered antibiotics in 26 patients and reported that the results were better in 24 patients (92.3%), worse in 2 patients (7.7%), and no change in 0 patient.

Wananukul *et al*.^205^ topically and/or systemically administered antibiotics in 41 patients with ulcerated hemangioma and reported improvement in 19 patients (46%).

Pandey *et al*.^206^ treated 608 patients showing ulcer formation with an ointment containing an antibiotic (mupirocin, sodium fusidate, sisomicin, or metronidazole) combined with systemic administration of an antibiotic (amoxiclav at 20–40 mg/kg/day) in those with ulcers with an area of >10 cm^2^ and examined the effectiveness of treatment according to the time until cure. The time until cure was 32.63 ± 13.06 days in superficial lesions, 42.89 ± 19.89 days in mixed lesions, and 57.03 ± 16.12 days in extensive lesions, with a mean of 40.09 ± 19.41 days in all lesions combined, showing significant differences among the 3 groups (*P* < 0.05). They also reported that the time until cure was significantly longer in larger (>10 cm^2^) than smaller ulcers (*P* < 0.05).

- Dressings

Kim *et al*.^204^ treated 25 patients using dressings and reported that the results were better in 23 patients (92%), and no change in 2 patients (8%). Oranje *et al*.^207^ applied polyurethane film and reported rapid relief of pain and cure of ulcer in 1–2 months. In addition, Bauland *et al*.^208^ treated 41 patients using a non‐adhering dressing containing an antibiotic and reported that the results were good in 26 patients (63.4%), moderate in 5 patients (12.2%), and little change in 10 patients (24.4%).

- Laser therapy

In the 1980s–1990s, there were reports^209^, ^210^ of argon, NdYAG, and KTP, but recent reports^211^, ^212^ are primarily about treatment using dye laser. Morelli *et al*.^209^ treated 37 patients with ulcerated hemangioma by dye laser irradiation (SPTL1b® (Candela Corporation, Wyland, MA, USA), wavelength: 585 nm, spot size: 5–7 mm, irradiation power: 5–6.8 J/cm^2^, pulse width: 0.45 ms) and reported that the number of irradiations until cure was 1 in 26 patients (68%) and 2 in 8 patients (21%) and that the mean period from the first treatment until cure of ulcer was 2.84 ± 0.22 weeks. Lacour *et al*.^210^ irradiated 8 patients with ulcerated hemangioma that resisted conventional treatments using the same equipment and reported acceleration of cure. David *et al*.^211^ performed dye laser irradiation (PhotoGenica V® (Cynosure, Westford, MA, USA), wavelength: 585 nm, spot size: 5–7 mm, irradiation power: 5–6.8 J/cm^2^, pulse width: 0.3–0.5 ms) in 78 patients and reported the effectiveness of laser therapy alone in 72 (92.3%). Also, Michel^212^ performed 1 or 2 irradiations using Dermobeam 2000^®^(Deka MELA, Calenzano, Italy) with a cooling system 595 nm (2 pulsed irradiations with a 10% overlap, spot size: 7 mm, irradiation power: 4–8 J/cm^2^) and reported resolution of pain in 10 of the 12 patients. Moreover, Di Maio *et al*.^213^ performed laser treatment in 65 patients with hemangioma with ulcer and reported that the effect was excellent and that no clear adverse events were observed, because scarring, which was noted in a few patients, did not differ markedly compared with scarring that occurs after conventional treatments.

However, Kim *et al*.^204^ treated 22 patients with pulsed dye laser and reported that the results were better in 11 patients (50%), worse in 1 patient (4.5%), and no change in 4 patients (18.2%), but warned that 5 patients in the proliferating phase showed ulcer formation after irradiation.

As observed above, although there have been several reports of the effectiveness of laser therapy against ulcer as factors of “benefit,” many reports are relatively old and lack controls, and the evidence is not considered sufficient. Further accumulation of cases is necessary. Laser may be effective in limited patients, but as there is the risk of ulcer formation as an adverse effect of laser irradiation of infantile non‐ulcerated hemangioma, greater caution is needed in treating already ulcerated lesions.

- Steroids

There have been few reports on steroid therapy focusing on ulcer. Kim *et al*.^204^ treated 7 patients by local steroid injections and reported that the results were better in 4 patients (57.1%), worse in 1 patient (14.3%), and no change in 1 patient (14.3%). They also systemically administered steroid to 22 patients and reported that the results were better in 16 patients (72.7%), worse in 1 patient (4.5%), and no change in 5 patients (22.7%). Based on these results, they considered that the treatment was effective for reducing the lesion size, and there are few other reports suggesting the effectiveness of steroid. Considering that the patients are infants and that there are other treatment options, steroid cannot be recommended at present.

- Topical preparations of recombinant human platelet‐derived growth factor

0.01% becaplermin (Regranex® (Ortho‐McNeil Pharmaceutical, Raritan, NJ, USA)) is a preparation for diabetic foot ulcer approved by the FDA in 1997. Sugarman *et al*.^214^ and Metz *et al*.^215^ reported its effectiveness for the treatment of ulcerated hemangioma in 1 and 8 patients, respectively, but its effectiveness cannot be appraised at present because of the small number of cases.

#### CQ16: Is intralesional corticosteroid injection more effective than systemic administration for infantile hemangioma?

*Recommendation*: Treatment using corticosteroid is effective for inducing early regression of hemangioma. While no significant difference is observed in the effectiveness between intralesional injection and systemic administration, attention to complications including those at the administration site, such as the periocular region, on local injection and those, such as hypertension and growth retardation, on systemic administration is necessary.

*Strength of recommendation*: 2 (wean)

*Evidence*: B (moderate)

*Comments*: As a result of primary screening, 99, 9, and 35 papers were extracted from PubMed, Cochrane, and JCRM, respectively, and 4 papers in English were subjected to secondary screening for this CQ. There was 1 report of an RCT, but the other reports were about case series while they evaluated a large number of cases. In addition, 2 papers on complications considered important in relation to intralesional corticosteroid injection for periocular lesions were added by manual search. Since there is a report of an RCT, and since other case series studies with a large number of subjects presented the results that there was no significant difference in the effectiveness of corticosteroid depending on the administration method, the strength of evidence was rated as “B.”

There was 1 report^216^ of an RCT focusing on “Is intralesional corticosteroid injection more effective than systemic administration for infantile hemangioma?.” In this trial, the subjects were divided into control, oral administration (prednisolone at 2 mg/kg/day every other day for 6 weeks), and intralesional injection (triamcinolone at 1–5 mg/kg with a maximum of 30 mg once a month for 6 months) groups, and the lesion size was significantly reduced in the treated groups compared with the control group. While no significant difference was noted between the oral administration and the intralesional injection groups, the reduction rate tended to be larger in the local injection group, and local injection was concluded to be slightly superior.^216^

There were reports^217^, ^218^ of case series with more than 1,000 subjects, but the findings were not statistically analyzed. Although both intralesional injection and oral administration were effective, there was also a mixed group of intralesional injection and oral administration, the condition of patients varied among the 3 groups (intralesional injection, oral administration, mixed), and the effectiveness according to the administration method was not shown. Regarding complications, systemic symptoms, such as hypertension, retarded body weight gains, and cushingoid appearance, were reported to be more frequent on oral administration than intralesional injection.^217^, ^218^ Moreover, concerning complications, in one report,^219^ periocular lesions were excluded from the targets of intralesional injection to avoid its effect on visual function. Indeed, there have also been case reports^220^, ^221^ that visual impairment was caused by occlusion of the retinal artery after intralesional corticosteroid injection for periocular hemangiomas. Currently, in Japan, intralesional corticosteroid injection is a treatment unapproved by the national health insurance system.

#### CQ17: Is topical therapy effective for infantile hemangioma?

*Recommendation*: Although it must be noted that there are no reports of comparison with placebo and that the degree of improvement is smaller compared with systemically administered drugs, topical medication can be an option for the treatment of infantile hemangioma with no risk of complications if drugs with milder adverse effects are selected.

*Strength of recommendation*: 2 (weak)

*Evidence*: C (weak)

*Comments*: As a result of literature searches, a total of 111 papers consisting of 70, 7, and 34 papers from PubMed, Cochrane, and JCRM, respectively, were extracted. They included 1 RCT study. Including this RCT, 48 papers were extracted by secondary screening. In addition to the papers selected by secondary screening as closely related to the CQ, a total of 47 papers obtained by manual search were adopted as reference for the preparation of guidelines. There was 1 RCT, and comparative studies of therapeutic results by topical therapy and case series studies with a relatively large number of subjects were adopted as papers of relatively high quality, the strength of evidence was rated as C “weak.”

In the reports related to the CQ:

1. Drug type: The drugs were classified into imiquimod, timolol, propranolol, corticosteroid, and others.^215^, ^222^, ^223^, ^224^, ^225^
2. Drug concentration and dosage form: Imiquimod was used as a 5% cream,^222^, ^226^, ^227^, ^228^, ^229^, ^230^, ^231^, ^232^, ^233^ timolol as 0.5% ophthalmic solution or gel,^222^, ^223^, ^232^, ^234^, ^235^, ^236^, ^237^, ^238^, ^239^, ^240^, ^241^, ^242^, ^243^ propranolol as 1% ointment,^181^, ^234^, ^241^, ^244^, ^245^ and corticosteroid were often used as ointments of agents ranked as relatively strong such as clobetasol propionate, halobetasol propionate, and betamethasone dipropionate.^246^, ^247^
3. Methods for topical application: Frequent administration methods were once a day every other day for imiquimod, 2 times a day every day at 1–2 drops each time for timolol, 2 times a day every day for propranolol, and 2 times a day every day for corticosteroid.
4. Methods for efficacy evaluation: Comparison of gross findings and photographs were adopted in all papers. The area was compared using photographs in one report.^227^ There was also a report^228^ of half‐side test for a control.
5. Adverse effects: No systemic adverse effect was reported, and most adverse effects were local. Imiquimod caused pain, flare, and erosion relatively frequently.^231^, ^233^ Few local adverse effects were reported for timolol and propranolol.^232^, ^234^, ^241^, ^242^, ^245^ No local adverse effects were reported also by corticosteroid.^246^, ^247^
6. Relative advantages of drugs Imiquimod has been reported to have usefulness comparable to that of topical beta blockers, but it is not considered superior in terms of adverse effects.^226^, ^228^, ^230^, ^232^

Corticosteroid was not shown to be superior in efficacy compared with beta blockers.

There was also one RCT study^181^ concerning the CQ, which is related to topical propranolol concerning drugs. In this RCT, 15 each of a total of 45 subjects were allotted to oral (propranolol at 2 mg/kg/day, 2 times a day), topical (1% propranolol water soluble ointment, applied 2 times a day), and local injection (1 mg/1 mL, 0.2 mL/1 cm in diameter, 1 mL/injection at the maximum, 1 time/week) groups. Ten patients (66.7%) in the topical group responded, but they were fewer than 13 (86.7%) in the oral group. The time until the appearance of the effect and time until complete cure were also longer in the topical group than in the oral group. Concerning complications, none was observed in the topical group, but 1 patient in the oral group showed unexplained syncope as an adverse effect and was excluded. While decreases in the heart rate and blood pressure were observed in 3 in the oral group, they did not necessitate interruption of the study. In the local injection group, 8 (53.3%) responded, but 3 were lost due to pain. From these results, the study concluded that topical therapy is an option to be evaluated for patients with a risk of adverse reactions to oral medication. While there were no reports of comparison under the same conditions, comparative studies of therapeutic results by topical therapy and case series studies with a relatively large number were adopted as relatively high‐quality papers. In all these reports, topical therapy of beta blockers (propranolol, timolol) was effective to an extent with no serious complications.

Thus, topical therapy, particularly, of beta blockers is considered generally useful, but there has not been a report of its comparison with placebo, and further accumulation of cases is necessary.

Research by comparison between dye laser treatment and topical beta blocker therapy is considered to be necessary.

#### CQ18: Is compression therapy effective for infantile hemangioma?

*Recommendation*: Although appropriate compression method must be performed for individual patients, compression therapy may be regarded as an option on condition that the therapy is carried out by a skilled physician. Sufficient attention to skin abnormalities and local/neighboring growth disturbance due to the compression are needed.

*Strength of recommendation*: 2 (weak)

*Evidence*: D (very weak)

*Comments*: While 23, 1, and 14 papers were extracted from PubMed, Cochrane, and JCRM, respectively, only 3 case reports remained to be reviewed as a result of primary and secondary screening. Thus, the evidence level is very low at D (very weak).

According to a case report of ulcerated infantile hemangiomas of the limbs by Kaplan *et al*.,^248^ the ulcers of most patients showed rapid improvements and cured within 2 weeks by compression therapy using the self‐adherent wrap Coban® (3M, St. Paul, MN, USA) combined with topical treatment with an antibiotic ointment (or early systemic antibiotic administration when secondary infection was apparent). They concluded that, compared with antibiotic ointment alone, its combination with compression therapy was more effective, and is a safe and easy treatment that promotes regression of hemangiomas.

Ochi *et al*.^249^ reported 12 cases of infantile hemangioma (9 girls and 3 boys with a mean age of 8.4 months; sites of the lesion: limbs in 6, head and neck in 5, and trunk in 1). By treatment using elastic bandages (5 patients), Presnet® (ALCARE, Tokyo, Japan) (4), supporter (1), or Elatex® (ALCARE, Tokyo, Japan) and cryotherapy (2), the hemangiomas disappeared or decreased in size in 11 of the 12 patients, with only 1 (head and neck) showing no improvement. The time until the disappearance of the lesion in the 11 responders was 2 months to 3 years (mean: 19.5 months), no complications associated with compression therapy were noted, and the authors recommended early initiation of compression therapy if the site of the lesion can be compressed.

Totsuka *et al*.^250^ treated 3 girls with parotid gland hemangiomas (mean age: 4.3 months) by splinting using a resin plate and compression using a handmade cap. The mean duration of treatment was 13 months (8–16 months), and the patients were followed up until a mean age of 4.6 years (2–7 years), resulting in clinical and echographic disappearance of hemangioma in all 3. Since infantile hemangiomas often regress spontaneously, it is impossible to conclude that they regressed due to compression therapy, but they reported the therapy to be safe and effective.

Thus, concerning factors related to “benefits” of compression therapy, there are reports that suggest the effectiveness of compression methods appropriate for sites (elastic bandages, Presnet, splinting with a resin plate). However, it must be noted that they are all old reports. Concerning factors related to “harm,” while compression is a relatively safe and simple method without reports of serious complications, the occurrence of dermatitis and growth disturbance at the site of compression or surrounding areas is considered possible. The recommendation level was set at 2D with consensus of the present guidelines preparation committee on condition that the treatment is performed carefully by a skilled physician in consideration of these points. The present guidelines do not exclude compression therapy, but it is necessary to consider oral propranolol, oral administration or local injection of steroid, and laser therapy first for infantile hemangiomas that need treatment.

#### CQ19: Is glucose transporter 1 (GLUT‐1) immunostaining useful for the diagnosis of infantile hemangioma?

*Recommendation*: Immunostaining for GLUT‐1 is positive in the proliferating, involuting, and involuted phases, shows high sensitivity and specificity, and is useful for the diagnosis of infantile hemangiomas if the clinical diagnosis is difficult.

*Strength of recommendation*: 2 (weak)

*Evidence*: C (weak)

*Comments*: To evaluate whether GLUT‐1 immunostaining is useful for the diagnosis of infantile hemangiomas, the literature was searched first for the following key words.

Infantile OR juvenile AND hemangioma AND marker AND immunohistochemistry

The search of JCRM resulted in 26 hits, but none of them performed analysis of GLUT‐1 or evaluated its usefulness by comparing infantile hemangioma with other hemangiomas/vascular malformations even if GLUT‐1 was analyzed. The search of PubMed resulted in 182 hits. From these papers, those that deserved detailed analysis were selected according to the following criteria.

1. Those in which GLUT‐1 immunostaining was performed for infantile hemangioma or other hemangiomas/vascular malformations.
2. Those that were retrospective epidemiological studies rather than reports of one case.

Fifteen research papers selected by these criteria were analyzed in detail.

In 7 of these reports,^251^, ^252^, ^253^, ^254^, ^255^, ^256^, ^257^ infantile hemangiomas were stained using GLUT‐1 simultaneously with other hemangiomas/vascular malformations, and differences in positive/negative results were evaluated. Of all cases reported in the 7 papers, GLUT‐1 was positive in 268 of the 273 cases of infantile hemangioma and negative in 244 of the 247 cases of lesions other than infantile hemangioma. There were also 4 papers^258^, ^259^, ^260^, ^261^ in which GLUT‐1 staining was performed for clinically typical infantile hemangiomas and hemangiomas that need to be differentiated from infantile hemangioma although they were not simultaneously stained in the same paper. When the 4 papers were combined, GLUT‐1 was positive in all 8 cases of infantile hemangioma and negative in all 49 cases of non‐infantile hemangioma. When the above cases are totaled, GLUT‐1 was positive in 276 of the 281 cases of infantile hemangioma and negative in 293 of the 296 cases of non‐infantile hemangioma, and the sensitivity and specificity of GLUT‐1 positivity for infantile hemangioma were 98.2% and 99.0%, respectively.

The usefulness of GLUT‐1 staining has also been confirmed by re‐evaluation of cases that were initially examined by hematoxylin‐eosin staining alone.^262^, ^263^, ^264^, ^265^ There have been 4 papers in which cases were re‐evaluated using GLUT‐1 staining, and 1 paper reported that the diagnosis was impossible by HE staining alone in 18% of the cases.^262^

#### CQ20: What gastrointestinal examinations are useful for children suspected to have blue rubber bleb nevus syndrome? When should the examinations be started?

*Recommendation*: It is recommended to start screening by examinations including blood tests and fecal occult blood test as early as possible. In children suspected to have gastrointestinal bleeding, the usefulness of endoscopic examination, red blood cell scintigraphy (^99m^Tc‐labeled red blood cells), and single photon emission computed tomography‐CT (SPECT‐CT) has been reported for the identification of the source of bleeding. If no abnormality is detected by screening, and search for gastrointestinal lesions needs to be performed to diagnose this disease or evaluate the future risk of bleeding, there is no standard for its timing. Among the examinations that led to the detection of gastrointestinal lesions in past reports, CT and MRI can be performed with relatively mild invasion and from an early stage.

*Strength of recommendation*: 2 (weak)

*Evidence*: D (very weak)

*Comments*: Gastrointestinal lesions of blue rubber bleb nevus syndrome (bean syndrome) are observed in the entire digestive tract, but they frequently appear, particularly, in the small intestine. Since it is an extremely rare disease, the literature is primarily case reports and reviews, and there have been no reports of clinical studies of many cases that are relevant for the CQ. Therefore, we investigated examinations that were useful for the detection of gastrointestinal lesions in reports, primarily, of child cases. Lesions in the small intestine are difficult to observe by conventional endoscopy, but techniques such as double‐balloon endoscopy, capsule endoscopy, CT enterography, CT, and MRI as well as upper and lower gastrointestinal endoscopy have been reported to be useful.^266^, ^267^, ^268^, ^269^, ^270^, ^271^, ^272^, ^273^, ^274^, ^275^, ^276^

As a result of database searching, 11 papers in English were adopted through primary and secondary screening. All papers selected by these screening processes were case reports or case series, and the strength of evidence is “D (very weak).”

There is no clear standard as to when the examinations should be initiated. However, neonates who developed gastrointestinal bleeding shortly after birth have been reported,^270^ and the earliest possible examinations are necessary if this disease is suspected. Invasive examinations are difficult to perform in small children, but blood tests (presence or absence of anemia or consumption coagulopathy) and fecal occult blood tests can be performed. If gastrointestinal bleeding is suspected, procedures such as endoscopy, particularly, double‐balloon endoscopy and capsule endoscopy, ^99m^Tc‐labeled red blood cell scintigraphy, and ^99m^Tc‐labeled red blood cell SPECT‐CT have been reported to be useful for the determination of the source of bleeding.^266^, ^268^, ^271^, ^275^

If no abnormality has been detected by screening tests, and if search for gastrointestinal lesions needs to be performed non‐emergently to diagnose this disease or evaluate the future risk of bleeding, there is no standard for the timing, which may vary among facilities. Among the above examinations, CT and MRI can be performed earlier and with relatively milder invasion, and are worth attempting if this disease is suspected. The necessity of the other examinations for the gastrointestinal lesions mentioned above should be considered when the patient reaches the age that tolerates the examinations.

#### CQ21: How are limb overgrowths to be managed in vascular malformations and syndromes?

*Recommendation*: If leg‐length inequality is insignificant, shoe lift is recommended. As significant inequality causes gait disturbance complicated with scoliosis, surgical treatment aimed to arrest epiphyseal growth is performed in the growth period. Shortening of the femur or tibia may be performed as an additional treatment. Bone elongation of the intact side is considered effective for the correction of leg‐length inequality.

*Strength of recommendation*: 2 (weak)

*Evidence*: D (very weak)

*Comments*: As a result of literature searches, 40 papers in English and 4 papers in Japanese were retrieved by primary screening. Of these papers, 17 in English and 4 in Japanese were extracted by secondary screening. As for the control of overgrowth of limbs, measures against leg‐length inequality and soft tissue hypertrophy are separately discussed and regarded as effective, but these papers were all classified either as case reports or as general discussions. Therefore, the evidence level is rated as “very weak,” and the recommendation level as “weak.”

In vascular malformations, typical disorders with hypertrophy of the affected limbs are Klippel‐Trenaunay syndrome and Parkes Weber syndrome and most of the papers refer to the management of limb overgrowth due to vascular malformations were written about these disorders. The literature regarding lesions at different sites is commented on below.

*Lower limbs*: In most reports, treatment for the overgrowth of the lower limbs were aimed to prevent physical disorders caused by leg‐length inequality. Some reports particularly mentioned treatment for foot lesions.

1. Correction of leg‐length inequality

If the leg‐length inequality is ≤2 cm, the management of leg length difference and accompanying scoliosis is considered possible by the use of shoe lift.^277^, ^278^, ^279^, ^280^, ^281^ If the leg‐length inequality is ≥2 cm, significant gait disturbance, postural abnormalities, and compensatory changes in the contralateral limb are likely to develop, and before consequent unphysiological gait leads to irreversible impairment, surgical treatment to correct the leg‐length inequality should be considered.^277^, ^278^, ^279^, ^280^, ^281^ Long‐leg radiography is useful for determining the best time for surgery,^282^ and the measurement of the leg length by long‐leg radiography or CT is reported to be effective.^278^ Surgical treatment reported in the papers are as follows.

*Treatment for overgrown limbs affected by vascular malformations*: Jacob *et al*.^277^ performed epiphysiodesis in 41 patients with a leg‐length inequality of ≥2 cm among 252 patients with Klippel‐Trenaunay syndrome and reported improvement in more than 90% of the patients. The effectiveness of this surgery is also confirmed by other review articles.^277^, ^278^, ^279^, ^280^, ^281^ The effectiveness of shortening of the femur and tibia was reported in the review by Capraro *et al*.^278^ The fixation period is considered to be shortened as a whole by simultaneously performing femoral or tibial shortening in addition to epiphysiodesis. Redondo *et al*.^280^ recommended endoscopic growth control of the epiphyseal plate in the distal end of the femur for patients with a leg‐length inequality of ≥2 cm. Capraro *et al*.^278^ did not recommend growth control with the epiphyseal stapling because of the unpredictability of the results and high frequency of complications. The appropriate time for surgical intervention on affected limbs is reported to be around the age of 11 years.^280^

*Elongation of the intact leg*: Tanaka *et al*.^283^ performed bone elongation of the intact limbs using an external fixator in adult patients with mild structural scoliosis and reported that the procedure was effective for correcting the leg‐length inequality and scoliosis. Jacob *et al*.^277^ also recommended bone elongation of the intact limb using Ilizarov external fixation apparatus in their review.

*Popliteal vein ligation*: Servelle^284^ hypothesized that elongation of the affected limbs was due to a high venous pressure and performed ligation of the popliteal vein of the intact limb in 48 children, and they reported significant improvement in leg‐length inequality. However, there are also negative views, saying its effectiveness is uncertain.^278^

- Foot lesions

Redondo *et al*.^280^ recommended resection of the toes (ray resection) and debulking for wearing shoes and cosmetic improvement. Gates *et al*.^285^ notably reported that compared with ray resection, wound healing of the stumps was poor after major resection.

*Upper limbs*: Asymmetry due to hypertrophy of the upper limb less frequently causes impairment of ADL than that of the lower limb. In one article, resection in patients with functional impairment due to marked finger deformities is reported,^282^ but articles reporting treatment for upper limb overgrowth are very few. While debulking has been reported to be advantageous from the cosmetic viewpoint,^279^ it has also been reported to induce exacerbate of edema of the affected limb,^284^ causing complications including cicatricial contracture, recurrence of the lesion, and refractory ulcer,^278^ and sufficient caution is necessary.

#### CQ22: Is surgical resection effective for soft tissue/superficial LM?

*Recommendation*: Although surgical resection is an effective treatment, it should be performed after comprehensive evaluation of cosmetic aspects, prognosis, functional prognosis, resectability, and possibility of recurrence/complications.

*Strength of recommendation*: 2 (weak)

*Evidence*: D (very weak)

Comments

*[Process of preparation of recommendation]*

Surgical resection is one of the major treatment options performed for LM. Although LM can be cured by total resection, the objective of treatment is not necessarily total resection, because the disease is not malignant, and surgical resection is often carried out for cosmetic, functional, and symptomatic improvements. Cosmetic problems are considered to be particularly serious if the lesion is located in superficial areas such as the body surface and soft tissue. However, surgical resection has been known to cause complications including hemorrhage, infection, deformation, and nerve paralysis.

In evaluating whether resection is effective, the balance between its positive aspects and negative aspects, such as complications, is important. For soft tissue/superficial LM, in which cosmetic improvement is important, problems including in what situations resection can be performed, whether there are criteria for the selection of resection, and, as there are differences in the incidence of complications, cure rate, and recurrence rate depending on the circumstances, whether its indications should be evaluated under different conditions are unclear. Therefore, the CQ, “Is surgical resection effective for soft tissue/superficial LM effective?,” was formulated, and the current knowledge was summarized.

*<Literature search and screening>*

As a result of literature search, 105 papers in Japanese and 348 papers in English were subjected to primary screening. Of these papers, 5 in Japanese and 42 in English were subjected to secondary screening concerning this CQ. They did not include papers with a high level of evidence, such as a systematic review and RCT, and all of them were case series or case reports. As a result, in the evaluation of this CQ, the results and discussion in each case series were integrated.

*<Review of observational studies (case series)>*

The effectiveness of resection of LM was evaluated from the following 5 viewpoints: (1) Effectiveness regarding the prognosis (mortality), (2) resection rate of the lesions (resectability), (3) functional outcome after resection (function), (4) recurrence rate (recurrence), and (5) complications.

*Results of review*

Generally, the rate of successful surgical resection is high, and ≥90% resection is reported to be possible in 60% or more of the patients.^286^, ^287^, ^288^ This also applies to the head and neck region, which is the frequent site of the lesion.^286^ However, the percentage of resectable lesions decreases from the cystic to mixed and to cavernous type.^286^ Since many LM are distributed diffusely in the skin and subcutaneous adipose tissue and around structures including muscles, blood vessels, and nerves, resection of the lesion involves resection of normal tissues in varying degrees. In lesions that show complicated distribution in the head and neck region, complications after surgical resection are observed relatively frequently. Serious complications including nerve paralysis, hematoma, local necrosis, sepsis, deformation, salivary fistula, hoarseness, airway obstruction, and malocclusion have been reported,^286^, ^287^, ^289^, ^290^, ^291^, ^292^, ^293^, ^294^, ^295^, ^296^, ^297^ and facial nerve paralysis is likely to result from resection, particularly, of LM infiltrating the parotid region.^289^ By the site, the incidence of complications increases as the area of involvement widens from unilateral to bilateral, from below to above the lingual bone, both sides, and both above and below the lingual bone.^293^, ^296^ Postoperative death may occur in patients with a severe neck lesion, but the extent of the effect of surgical resection is unclear.^287^, ^288^, ^298^ Postoperative recurrence is closely related to the resectability of the lesion depending on its distribution, and lesions that are difficult to resect due to a wide area of involvement and a strong tendency of infiltration have been reported to be associated with recurrence.^296^

*Limitations*

Indications for surgical resection vary among papers, and differences in the patient background must be considered in the evaluation of the effectiveness of resection. While there were many reports that surgical resection was performed in combination with sclerotherapy, and resection is considered to have been performed when more favorable results were expected from resection rather than sclerotherapy, criteria for their selection are unclear. Therefore, there is certainly the large bias of individual variation in the circumstances, and it was clearly impossible to conclude that resection is uniformly effective.

## <Summary>

While the effectiveness of surgical resection for soft tissue/superficial LM was evaluated, there was no literature with a high level of evidence. One of the major reasons is the diversity of the lesion type (cystic or cavernous), area of involvement, and history of other treatments. Because of this diversity, the condition of patients is considered to show an extremely wide variation, and their generalization is impossible. However, if conditions, such as the type of the lesion (cystic or cavernous), site of origin, and relationship with other treatments are restricted, tendencies were observed in functional prognosis, recurrence rate, and contents and incidence of complications.

While the resection rate of lesions by surgical treatment was suggested to be generally high, selection criteria for resection were unclear. Therefore, it is speculated that resection was performed for patients clinically judged to be treated more effectively by surgery. However, since there were some serious complications of surgical resection that persist as sequelae, their possibility should be evaluated carefully in performing surgical resection. The risk of resection has been suggested to vary with conditions of the lesion. The functional outcome is poor, and the recurrence rate and incidence of complications after resection are high, in those that occupy a wide area and those that are accompanied by symptoms such as airway obstruction.

From these observations, we propose at present, “While surgical resection is often effective, it must be selected in consideration of cosmetic aspects, prognosis, functional prognosis, resectability, and possibility of recurrence and complications,” despite limited scientific grounds. If complete resection of the lesion is possible, surgical resection may be performed as the first line treatment, but the possibility of other treatments including sclerotherapy, in particular, should be evaluated according to the diverse conditions of individual patients, and surgical resection should be performed when other treatments are ineffective or when surgical resection is considered clearly superior.

### CQ23: What is the optimal timing of surgery for soft tissue/superficial LM?

*Recommendation*: It is impossible to recommend optimal timing of surgery, and judgments according to the condition of each case are necessary.

*Strength of recommendation*: 2 (weak)

*Evidence*: D (very weak)

Comments

*[Process of preparation of recommendation]*

Soft tissue/superficial LM are not malignant lesions. Emergent treatment may be necessitated by life‐threatening symptoms, such as airway obstruction, but the initiation of treatment immediately after the diagnosis is generally considered unnecessary. The natural course of the disease differs considerably among individuals, particularly, in infancy, and the lesions may show a tendency of spontaneous regression but may also cause various functional problems due to rapid enlargement. Moreover, there are cosmetic problems characteristic of this disease in addition to functional problems, and early therapeutic effects are necessary to make social life comfortable. For these reasons, the selection of optimal timing of treatment, surgery in particular, is a major issue.

For the selection of the timing of surgical resection, conditions to obtain the best results as well as indications for resection must be evaluated, and sufficient consideration of the balance between merits and demerits depending on the timing of resection is necessary. Therefore, in this CQ, we attempted to summarize the presently available knowledge about “What is the optimal timing of surgery for soft tissue/superficial lesions?”.

*<Literature search and screening>*

As a result of literature search, 67 papers in Japanese and 231 papers in English were subjected to primary screening. Of these papers 5 in Japanese and 42 in English were subjected to secondary screening for this CQ. They included none with a high level of evidence, such as a systematic review and RCT, and all papers were case series or case reports. Therefore, the results and discussion in each case series were integrated in the evaluation of this CQ.

*<Review of observational studies (case series)>*

Defining “the optimal timing of surgery” mentioned in the CQ as “the timing of surgery at which good results can be obtained,” we aimed to evaluate the timing of surgery at which resection is effective, problems, such as complications are few, and, i.e., “the best results” can be obtained as a whole. Conditions must be evaluated on the basis of the timing in addition to the effectiveness of surgery, but objective judgments were considered difficult in this evaluation. However, as it was considered possible to obtain information about the age and time of surgery from the literature reviewed in the previous CQs concerning the effectiveness, papers that evaluated the age at surgery were searched.

*Results of review*

Despite a careful review of the literature by secondary screening, there was no paper that analyzed cases from the viewpoint of optimal timing of surgery. There was information concerning the age at surgery, but its appropriateness was not evaluated. Papers that mentioned the timing of surgery are shown below.

Concerning the timing (age) of surgery, unless the size of the lesion is small or there are symptoms that require urgent treatment, such as respiratory disturbance, it is recommended to delay surgery until the age of 3 years by expecting spontaneous regression or for the ease of identification of surrounding structures during surgery, ease of control of bleeding, and less problems with postoperative management.^294^ There was also a paper^295^ that suggested the necessity of the determination of the time of surgery in consideration of problems that change with age including the priority of securing the airway and appropriate nutritional management in neonates with head and neck and giant lesions, control of hemorrhage and infection and measures to prevent dysarthria and dental problems in infants, and skeletal and cosmetic problems in school‐age children, although it did not mention the optimal timing of surgery.

However, there was no paper that positively recommended resection without considering the time after the diagnosis or grounds for such a recommendation.

## <Summary>

As a result of literature search for evaluating the CQ, “What is the optimal timing of surgery for soft tissue/superficial LM,” there were papers that mentioned the timing of surgery, but none of them objectively evaluated its appropriateness. Therefore, no suggestion about the appropriate timing of surgery was obtained from the literature available at present, but there were a few papers suggesting that the decision to perform surgery should be made carefully.

Similar to the previous CQ, soft tissue/superficial LM of which the background vary in individual cases, and it is difficult to uniformly evaluate the effectiveness of resection. In clinical practice, in addition to medical reasons, social reasons including school attendance are considered to largely influence the decision of the time of resection. The results of RCTs are necessary to obtain objective data, but it is practically very difficult to arrange an RCT fulfilling the above conditions.

While this CQ is a very important issue for patients and families as well as clinicians, there has not been objective evaluation of the optimal timing of surgery in the past. Presently, as quick decisions to perform surgery should be avoided, this guideline proposes, “The optimal timing of surgery cannot be decided in general, and judgments according to the condition of each case are necessary.”

### CQ24: Is sclerotherapy effective for facial microcystic LM?

*Recommendation*: A wide range of drugs are used for sclerotherapy. Although comparison among drugs has not been made, and consensus regarding the methods or frequency of their administration has not been reached, improvements are observed after sclerotherapy in various symptomatic, functional, and cosmetic (esthetic) aspects. However, complications including functional impairment have also been reported.

*Strength of recommendation*: 2 (weak)

*Evidence*: D (very weak)

Comments

*[Process of preparation of recommendation]*

*[Literature search and screening]*

Concerning this CQ, 35 papers in Japanese and 92 papers in English (60 from PubMed, 32 from Cochrane) were retrieved. After their primary screening, 6 in Japanese and 18 in English were subjected to secondary screening concerning this CQ. Although they included 3 RCTs, many of the other papers were case series or case reports. Therefore, in the evaluation of the draft recommendation concerning this CQ, the results and discussion in each RCT and case series were integrated. While the evidence is scarce, the papers judged to be useful for the preparation of the draft recommendation are presented as review data.

*[Review of case series]*

As a result of literature screening, it was found that the effectiveness of sclerotherapy for facial microcystic LM has been evaluated from the following viewpoints.

1. Treatment responses
  - Size
  - Symptoms
  - Functions
  - Cosmetics
2. Complications

The contents of the accounts concerning the effectiveness of sclerotherapy are summarized according to these viewpoints.

However, there were few reports that exclusively analyzed facial (and microcystic) LM, and lesions of the neck and other regions as well as the face were evaluated or different types of LM, such as cystic and mixed types, were reported together. In addition, the definition of the cavernous type and standard procedure of sclerotherapy (method and number of administrations) varied among reports, and these differences in the background should be considered in the evaluation of the effectiveness of sclerotherapy.

The sclerosing agents used for the literature search ranged widely from OK‐432, bleomycin, ethanol, doxycycline, and sodium tetradecyl sulfate (STS). However, as none of the papers reviewed for the preparation of these guidelines evaluated differences in the effectiveness for facial microcystic lesions among drugs or the method or number of administrations of each drug, these evaluation items were excluded in discussing this CQ.

1. Responses

*A. Size*

Many of the papers that referred to the regression rate of the lesion classified the responses into (1) excellent or complete (regression rate ≥90%), (2) good or substantial (regression rate ≥50% and <90%), (3) fair or intermediate (regression rate ≥20% and <50%), and (4) poor or none (regression rate <20%).

Although there was no paper that collected cases of facial lesions alone, Yang *et al*.^299^ reported that the regression rate after sclerotherapy was ≥90% in 19 (63%) of the 30 patients with head and neck lesions and ≥50% in 10 (33%). In addition, the regression rate was reported to be ≥50% in 18 (85.7%) of the 21 patients with head and neck lesions by Alomari *et al*.^300^ and in 30 of the 31 patients to be ≥50%, who included those with mixed type lesions, by Chaudry *et al*.^301^

Smith *et al*.^302^ reported that none showed a response (complete or substantial) in 17 patients who underwent sclerotherapy, some of whom had mediastinal lesions. Giguere *et al*.^303^ also reported that all 5 patients with head and neck lesions showed no response (poor) to the therapy. While these studies were RCTs evaluating the time of sclerotherapy, the results suggest that sclerotherapy is not effective for microcystic lesions regardless of the time of treatment.

There was no paper that compared sclerotherapy and resection for facial microcystic LM.

*B. Symptoms*

There is no literature that evaluated this item based on objective data, and few reports referred to symptoms themselves. The information was limited to the report by Chaudry *et al*.^301^ that symptoms disappeared after sclerotherapy using bleomycin in 75% of the patients who complained of pain and a few case reports^304^, ^305^ that symptoms, such as hemorrhage and respiratory impairment, were relieved after sclerotherapy.

*C. Functions*

Ravindranathan *et al*.^306^ performed sclerotherapy in 3 patients with diffuse microcystic lesions extending from the face to the tongue and pharynx and reported that respiratory impairment and swallowing disorder due to airway obstruction observed before treatment were mitigated. Poonyathalang *et al*.^307^ administered STS to a patient with orbital lesions primarily complaining of visual defect and reduced visual acuity due to retrobulbar hemorrhage and reported alleviation of the symptoms, but relevant literature was scarce similar to that concerning symptoms.

*D. Cosmetic aspects*

Cosmetic improvements are difficult to evaluate objectively. Poonyathalang *et al*.^307^ administered STS to 3 patients with orbital lesions with exophthalmos as the primary symptom and reported improvement by measuring the degree of protrusion before and after the treatment. There have also been reports of objective assessment based on the degree of satisfaction in the patients’ families. According to Chaudry *et al*.,^301^ all patients with head and neck lesions (9 with microcystic lesions, 22 with mixed lesions) and their families reported improvements in the size and appearance of the lesions. In addition, Alomari *et al*.^300^ treated 32 patients with mostly microcystic but including some cystic LM of the head and neck region by sclerotherapy and reported improvements compared with the condition before treatment by the families of 26 patients (81.3%).

- Complications

As complications in the facial region, there are a large number of reports^299^, ^301^, ^307^, ^308^, ^309^, ^310^, ^311^, ^312^ of transient complications associated with sclerotherapy, such as fever, local swelling and pain, intracystic hemorrhage, and infection, although the lesions were poorly characterized in some reports. In addition, complications considered to have been caused by the effect of treatment, such as ulcer of the oral mucosa and tongue, facial nerve paralysis, leakage of saliva, and respiratory insufficiency due to airway obstruction, have been occasionally reported.^303^, ^306^, ^307^ There have also been reports^307^, ^313^, ^314^ of an elevation of the intraorbital pressure, exophthalmos, intraorbital hemorrhage, corneal damage, and external ocular muscle paralysis due to enlargement of the mass after sclerotherapy for ocular LM. There was also no literature showing the incidence of complications in facial microcystic LM.

As complications caused by sclerosing agents, skin ulcer and necrosis and nerve damage due to ethanol leakage, hypotension during anhydrous ethanol injection, and epidermal detachment due to doxycycline have been reported.^300^, ^315^ However, there was no report of serious complications due to OK‐432. Pulmonary fibrosis is widely known to be a complication of bleomycin, but, according to Chaudry *et al*.^301^ and Yang *et al*.,^299^ impairment of respiratory function does not occur at a dose routinely employed for sclerotherapy.

## [Summary]

In evaluating the CQ, “Is sclerotherapy effective for facial microcystic LM?,” analysis was performed from the viewpoints of responses to the treatment in terms of symptoms, functions, and cosmetic (esthetic) aspects and complications, but few papers with a high level of evidence were found. While the degree of regression of the lesions by sclerotherapy varied widely, the size‐reducing effect of the therapy was consistently small unlike that in cystic lesions. Some papers referred to symptoms, functional outcome, and cosmetic improvement, but they were insufficient for general discussion of sclerotherapy for facial microcystic LM. As complications characteristic of sclerotherapy, serious impairment may be caused by leakage of the sclerosing agent (ethanol, in particular), and this point needs attention. Based on the above observations, it is difficult at present to evaluate indications for sclerotherapy against microcystic LM by formulating criteria. Therefore, for the future, it is considered necessary to evaluate the usefulness of sclerotherapy addressed by this CQ by designs such as RCT.

### CQ25: Is sclerotherapy effective for intra‐abdominal LM?

*Recommendation*: Although there are many reports that sclerotherapy is useful, there is the risk of complications, and careful judgments about matters including the resectability of the lesion and selection of the sclerosing agent are necessary.

*Strength of recommendation*: 2 (weak)

*Evidence*: D (very weak)

Comments

*[Process of preparation of recommendation]*

LM is the most frequent lymphatic vessel disorder of the abdomen. Intra‐abdominal lesions are estimated to account for 10–20% of all LM, and the selection of treatment is difficult depending on the site of the lesion. While surgical resection is expected to be effective, less invasive treatments are considered necessary in view of stress to the patient and the possibility of severe complications such as lymphatic fluid leakage and bowel obstruction. Sclerotherapy, which is a major treatment for LM, is considered to be less invasive than surgery. Although positive therapeutic effects are expected, sclerotherapy is known to induce marked inflammation. Whether it can be performed safely without negative effects including complications and its long‐term effects are major clinical concerns. In addition, what therapeutic effects are expected or what complications should be anticipated after sclerotherapy for the intra‐abdominal lesion is also unclear. Therefore, the CQ, “Is sclerotherapy effective for intra‐abdominal LM?”, was formulated, and knowledge available at present was compiled.

*<Literature search and screening>*

As a result of literature search, 19 papers in Japanese and 38 papers in English (32 from PubMed, 6 from Cochrane) were subjected to primary screening. Of these papers, 2 in Japanese and 9 in English were subjected to secondary screening concerning this CQ. They included no papers with a high level of evidence, such as systematic reviews and RCTs, and all were case series or case reports. Consequently, the results and discussion in each case series were integrated in the evaluation of this CQ.

*<Review of observational studies (case series)>*

The literature concerning the effectiveness of sclerotherapy for intra‐abdominal LM was reviewed from the viewpoints of (1) therapeutic effects (decrease in lesion size, symptoms) and (2) complications.

The drugs used for sclerotherapy ranged widely from OK‐432 to bleomycin, ethanol, doxycycline, STS, acetic acid, steroid/tetracycline, and 50% glucose solution. According to our review, there was no paper that evaluated the differences in effectiveness of sclerotherapy in the abdomen according to the drug type or administration method or number of administrations of each drug.

*Results of review*

1. Therapeutic effects

*A. Regression rate of the lesion*

Regression of lesions of intra‐abdominal LM by sclerotherapy was mentioned in 5 papers.^288^, ^316^, ^317^, ^318^, ^319^ According to the report by Chaudry *et al*.,^316^ the reduction rate was ≥90% in 7 and ≥20% in 1 of the 10 patients with LM of the mesentery and retroperitoneum treated with doxycycline, and evaluation using imaging examination was not performed in 2 cases. The patient who showed a low regression rate had a mixed type of cystic and cavernous lymphangiomas, and the other patients had cystic lesions. Oliveira *et al*.^317^ reported that the lesion regressed by 70% in 1 of the 2 patients with cystic lymphangiomas treated with OK‐432. Won *et al*.^318^ reported 1 patient who showed complete disappearance of cystic retroperitoneal lesions after sclerotherapy using acetic acid. Shiels *et al*.^319^ reported that cystic lesions responded to sclerotherapy using STS and ethanol in 2 patients, but there was no mention about the reduction rate. However, according to Alqahtani *et al*.,^288^ no effect was observed in 10 patients who underwent sclerotherapy using steroid/tetracycline or 50% glucose solution.

*B. Symptoms*

There were 3 papers that referred to symptoms of patients treated by sclerotherapy for intra‐abdominal LM.^316^, ^317^

According to Chaudry *et al*.,^316^ of the 10 patients who underwent sclerotherapy, 3 had chronic abdominal pain, 3 had acute abdominal pain, 1 had fever/chill, 1 had anemia, and 2 had palpable masses, but the symptoms were alleviated by treatment in all patients, and no recurrence was noted.

Oliveira *et al*.^317^ reported that sclerotherapy was performed in a patient with a palpable mass and in 1 with a palpable mass, abdominal compartment syndrome, and a poor general condition. While the condition was alleviated in the patient who only showed a palpable mass after 2 courses of OK‐432 sclerotherapy, but the treatment was changed to surgery in the patient who had abdominal compartment syndrome because of enlargement of the mass due to intracystic hemorrhage.

- Complications

Three papers specifically mentioned complications of sclerotherapy for intra‐abdominal LM. There was no report of deaths due to treatment‐related complications. Oliveira *et al*.^317^ treated 3 patients by sclerotherapy using OK‐432 and reported that one of them developed subbowel obstruction after the treatment and another required emergency surgery due to exacerbation of abdominal compartment syndrome induced by intracystic hemorrhage. Chaudry *et al*.^316^ reported that doxycycline used for sclerotherapy leaked into the retroperitoneal space in 1 of the 10 patients but that the lesion regressed without any particular problem. Won *et al*.^318^ performed sclerotherapy using acetic acid in 1 patient with retroperitoneal cystic lymphangioma. Although pain and hematuria were observed, they concluded that the relationship of hematuria with the therapy was unclear, because it was observed during menstruation.

*Limitations*

Sclerotherapy was often performed before, after, or during surgical resection, and papers that reported the results of sclerotherapy alone were few. There was no paper that directly compared observation without treatment, sclerotherapy, and surgical resection. Few papers analyzed intra‐abdominal lesions alone, and many papers included lesions in other areas or evaluated lesions in different intra‐abdominal regions including the mesentery, retroperitoneum, and viscera collectively.

Moreover, differences in properties of LM, such as cystic, cavernous, and mixed types, their definitions, criteria for the selection of sclerotherapy (combination with surgery, types of sclerosing agents and methods of their use, number of administrations) varied among papers, and few papers evaluated these matters separately.

Such differences in the patient background and contents of treatment must be considered in evaluating the effectiveness of sclerotherapy. In evaluating this CQ, particularly, differences in morphology of LM and sclerosing agents were excluded.

## <Summary>

The CQ, “Is sclerotherapy effective for intra‐abdominal LM?” was evaluated from the viewpoints of therapeutic effect, symptoms/functions, and complications, but no paper with a high level of evidence was found. While sufficient regression of the lesion and alleviation of symptoms were achieved by sclerotherapy in some patients, the response rate varied among reports, and information was insufficient for general discussion of sclerotherapy. Concerning treatment‐related complications, there have been reports of bowel obstruction associated with sclerotherapy, and attention to this condition as well as intracystic hemorrhage is considered necessary. However, there was no report of chylorrhea, which was reportedly caused by surgery.

Based on the above observations, it is presently difficult to determine indications for sclerotherapy in intra‐abdominal LM by setting up criteria, but as there was no literature that strongly ruled out intra‐abdominal LM as indications of sclerotherapy, these guidelines propose, “Although there are many reports that sclerotherapy is useful, there is the risk of complications, and careful judgments about matters including the resectability of the lesion and selection of the sclerosing agent are necessary.” For the future evaluation of this CQ, validation by a design with a high level of evidence, such as RCT, is considered necessary.

### CQ26: Are patients with scarcely symptomatic intra‐abdominal LM candidates for treatment?

*Recommendation*: Since there is risk of treatment‐related complications, it is proposed to consider therapeutic intervention when the lesion tends to enlarge or has become symptomatic.

*Strength of recommendation*: 2 (weak)

*Evidence*: D (very weak)

Comments

*[Process of preparation of recommendation]*

Intra‐abdominal LM occasionally presents with severe symptoms, such as abdominal pain, giant mass, and bowel obstruction, but may also be asymptomatic and detected incidentally. Lesions may gradually enlarge and cause serious symptoms due to infection and intraluminal hemorrhage.

Under such circumstances, whether patients with nearly asymptomatic intra‐abdominal LM should be aggressively treated, or when intervention should be optimally performed during their long follow‐up period are major problems that pose clinical dilemma. Therefore, the CQ, “Are patients with scarcely symptomatic intra‐abdominal LM candidates for treatment?,” was formulated, and knowledge available at present was summarized.

*<Literature search and screening>*

As a result of literature search, 206 papers in Japanese and 237 papers in English (230 from PubMed, 7 from Cochrane) were subjected to primary screening. Of these papers, 6 in Japanese and 9 in English were subjected to secondary screening concerning CQ 26. They included no papers with a high level of evidence, such as a systematic review or RCT, and many of them were case series or case reports. Since 7 papers among them described asymptomatic LM, their results and discussions were integrated to answer the CQ.

*<Review of observational studies (case series)>*

Seven papers^316^, ^317^, ^320^, ^321^, ^322^, ^323^, ^324^ among reviewed literature described asymptomatic LM. Fifteen cases reported in these papers were considered to have actually presented few symptom (including asymptomatic patients who were incidentally detected by imaging studies to have intra‐abdominal masses at the sites as greater omentum, mesentery and retroperitoneum).

The literature was screened, and papers addressing issues concerning therapeutic intervention for scarcely symptomatic intra‐abdominal LM including “What symptoms they may present with if they are left untreated?,” “By what studies and how often should they be examined?,” and “What other treatments are available and how serious are complications or risk of each treatment?” were reviewed.

*Results of review*

From the literature reviewed, symptoms of intra‐abdominal LM (abdominal pain, bowel obstruction, torsion, infection, hemorrhage, vomiting/sucking difficulty, frequent urination and abdominal mass^320^, ^321^, ^322^, ^323^, ^324^, ^325^, ^326^) are considered to be dependent on factors such as site, size and age. It is necessary to determine risk factors by stratification of these factors in the future.^320^, ^322^, ^325^

Reported complications in treated cases include recurrence that required re‐treatment,^321^ bowel obstruction,^317^, ^323^, ^324^ chylous ascites,^324^, ^326^ embolism,^317^ hemorrhage^317^ and wound infection. Embolism of the inferior vena cava after surgery^317^ and abdominal compartment syndrome after adhesion therapy^317^ were reported as severe complications. It deserves special attention that, if surgical resection is performed for mesenteric LM, the intestine may have to be resected with the lesion.^326^

While there have been reports^320^, ^322^ that intra‐abdominal LM with few clinical symptoms regressed during follow‐up, they may become symptomatic later (as observed in many case reports). For that reason, the opinion that intervention should not been chosen during the follow‐up until the lesion enlarges or new symptoms appear was frequently described.

*Limitations*

It should be noted that many asymptomatic cases may be left unreported and some asymptomatic lesions that had been detected were treated. There is no study with a high level of evidence indicating explicit criteria concerning the age, site or situation about whether intervention should be made for asymptomatic intra‐abdominal LM.

## <Summary>

The necessity of treatment of a patient with intra‐abdominal LM with few symptoms should be determined after evaluating the balance between the risk of treatment and non‐treatment considering its site and size as well as patient age. However, since research on indications for treatments has been insufficient so far and serious complications after treatment have been reported, deliberate evaluation for each patient is mandatory. When observation is selected, periodic imaging studies are recommended to optimize therapeutic intervention by detecting enlargement of the lesion. Also if any symptom has developed during follow‐up, intervention should be considered. For these reasons, the recommendation, “Since there is risk of treatment‐related complications, it is proposed to consider therapeutic intervention when the lesion tends to enlarge or has become symptomatic.” was adopted.

### CQ27: What treatments are effective for refractory chylous ascites?

*Recommendation*: Conservative treatments, such as fasting, high‐calorie infusion, and medium chain triglyceride (MCT), should be performed first, but, if they are ineffective, drug treatment, sclerotherapy, and surgery may also be considered.

*Strength of recommendation*: 2 (weak)

*Evidence*: D (very weak)

Comments

*[Process of preparation of recommendation]*

Refractory chylous ascites causes loss of large amounts of protein and lymphocytes, decreases in the blood lipid levels, and abdominal pain, unpleasantness, and dyspnea due to abdominal distention and markedly reduces the quality of life. The cause of ascites often remains unknown. Treatment of chylous ascites may require drainage to avoid abdominal distention. It is a very important point for clinicians to make proper judgments by understanding treatments and their effects and demerits. Therefore, it is considered beneficial to collect information about chylous ascites over a long period and compile guidelines. For this purpose, the presently available knowledge was collected by formulating the CQ, “What treatments are effective for refractory chylous ascites?”

*<Literature search and screening>*

As a result of search, 161 papers in Japanese and 728 papers in English (564 from PubMed, 164 from Cochrane) were subjected to primary screening. Of these papers, 15 in Japanese and 12 in English were subjected to secondary screening for CQ 27. They included none with a high level of evidence, such as systematic reviews and RCTs, and consisted of 1 multicenter and 2 single‐center case series and case reports. Consequently, we used the results and discussion of 27 papers judged for the preparation of the draft recommendation were integrated although evidence was insufficient for the evaluation of this CQ.

*<Review of observational studies (case series)>*

As for causes of chylous ascites, congenital chylous ascites,^327^, ^328^, ^329^, ^330^, ^331^, ^332^, ^333^, ^334^, ^335^, ^336^, ^337^, ^338^, ^339^, ^340^, ^341^, ^342^ idiopathic chylous ascites,^328^ chylous ascites after laparotomy,^343^, ^344^, ^345^, ^346^ protein‐losing enteropathy,^345^ LM,^347^, ^348^ lymphangiectasis,^349^, ^350^ lymphangiomatosis,^351^, ^352^ and lymphatic dysplasia^353^ were reported. None of the papers evaluated treatments according to the cause.

When treatments are categorized, conservative treatments (fasting, high‐calorie infusion, MCT), drug treatments, sclerotherapy, and surgical treatment were performed.

*Results of review*

The results of review are presented below according to the treatment.

1. Conservative treatments

Whether the amount of ascites changes by fasting should be checked first.

High‐calorie infusion is often used with fasting, and since there was no report that ascites increased under the effect of high‐calorie infusion according to our review, it is recommended for nutritional support during fasting. In the multicenter case series reported by Bellini *et al*.,^327^ high‐calorie infusion/total parenteral nutrition was performed in 15 patients without adverse effects.

MCT was used before, after, and during treatment.^327^, ^328^, ^330^, ^331^, ^332^, ^333^, ^334^, ^335^, ^337^, ^339^, ^340^, ^341^, ^343^, ^345^, ^346^, ^348^, ^349^, ^350^, ^351^, ^352^ In the multicenter case series by Bellini *et al*.,^327^ MCT was reportedly performed in 14 patients without adverse effects.

- Drug treatments

In drug therapy for chylous ascites, primarily octreotide (a long‐acting somatostatin analogue) was used, and no report that discussed the effectiveness of other drug therapies was found by the present literature search.

In the multicenter case series by Bellini *et al*.,^327^ octreotide was administered to 6 of the 16 patients with chylous ascites for 8–38 days, and a decrease in chylous ascites was reported in all of them. In the single‐center case series by Huang *et al*.,^344^ 2 of the 4 patients with chylous ascites treated by high‐calorie infusion and octreotide administration were reported to have shown a decrease in ascites within 10 days. However, there has been a report^330^ that no effect was observed despite the administration of octreotide for 3 weeks. Concerning the dose of octreotide, it was administered at 1 μg/kg/h,^327^ at 3 μg/kg/h,^332^ began to be administered at 0.5 μg/kg/h and increased to 10 μg/kg/h by 1 μg/kg/h,^329^ administered by continuous intravenous infusion at 0.5–2.0 μg/kg/h,^333^ and began to be administered by subcutaneous injection at 2.5 μg/kg 2 times/day and increased every 2 days to 8 μg/kg 2 times/day.^330^ Regarding the time of the beginning of administration, the administration was started as no improvement was observed in chylous ascites after conservative treatments for 2 weeks,^330^, ^334^ and as chylous ascites was alleviated by conservative treatments but was exacerbated again.^333^ No adverse effects of octreotide administration were noted in the present review of the literature. Thus, no control study that evaluated the effect of octreotide on chylous ascites was found by the present literature search, and the level of evidence concerning the efficacy is low, but as there are case series and many case reports that chylous ascites was reduced by octreotide administration, it appears reasonable to consider drug treatment using octreotide for chylous ascites that does not respond to conservative treatments.

- Sclerotherapy

Sclerotherapy was performed in 6 patients in 5 case reports.^339^, ^347^, ^349^, ^351^, ^352^ The sclerosing agent was OK‐432 in 5 of the 6 patients and was Beta‐isodona® (MUNDIPHARMA GmbH, Cambridge, UK)‐solution in 1.^349^ OK‐432 was locally injected into the lesion in 4,^347^, ^351^, ^352^ administered intraperitoneally in 1,^352^ and administered via the drain in 2.^347^, ^352^ Concerning sclerotherapy, the number of reported cases that were reviewed was limited, and further accumulation of cases is considered necessary to establish its usefulness.

- Abdominal drainage, abdominal puncture, and surgical treatment

Abdominal drainage and abdominal puncture are performed when organ compression symptoms (compartment syndrome and respiratory insufficiency) due to abdominal distention are present or possible or when the drain is inserted postoperatively. However, drainage itself cannot improve chylous ascites, and treatments, such as infusion, blood preparations, and blood transfusion, are necessary to supplement the ascites lost due to drainage.^327^, ^330^, ^331^, ^332^, ^333^, ^337^, ^338^, ^339^, ^340^, ^343^, ^345^, ^346^, ^347^, ^349^, ^351^, ^352^

Surgical treatment is reported to be frequently performed after conservative or drug treatments. According to the single‐center case series by Zeidan *et al*.,^343^ surgical treatment was performed in patients who responded poorly to conservative treatments for over a mean of 25.3 days. In other reports, surgical treatment was performed after conservative treatments for 1–3 months^328^, ^329^ and in patients with congenital chylous ascites 1–4 months after birth.^330^, ^334^, ^350^ Since it is often impossible to identify the leakage site of chylous ascites,^330^ attempts to identify the leakage site by orally administering a lipophilic dye (Sudan black, Sudan III) before operation.^328^, ^329^, ^336^, ^343^ When the leakage site can be identified, ligation, suturing, clipping, and cauterization have been performed.^328^, ^334^, ^336^, ^343^, ^350^ In addition to reports of the usefulness of techniques to stop leakage, such as applying or sprinkling fibrin glue at the leakage site of chylous ascites or over the surrounding retroperitoneum^329^, ^331^, ^343^, ^350^ and applying a patch of oxidized cellulose/resorbable local hemostatic agent,^331^, ^343^ there have also been reports of peritoneovenous shunting^349^, ^353^ and peritoneoamniotic shunting for fetal cases.^338^

There was no large clinical study in the past literature. Therefore, although the level of evidence is low, we consider that surgical treatment is recommendable for chylous ascites that does not respond to conservative or drug treatments, because it has been performed in case series and case reports for chylous ascites that did not respond to conservative or drug treatments for over about 1 month. Although techniques to enhance the response rate of surgical treatment, such as identifying the leakage site by using a lipophilic dye and applying fibrin glue or a patch of oxidized cellulose/resorbable local hemostatic agent, have been attempted, there are only case series and case reports, and none of the papers retrieved by the present literature search evaluated their usefulness.

*Limitations*

There was no literature that defined refractory chylous ascites based on the duration of illness or treatment responses. Therefore, we extracted and summarized factors that were considered to contribute to clinical refractoriness, such as the duration of illness and treatment responses, in each paper related to the treatment for chylous ascites. Also, as the cause of chylous ascites varies widely, the therapeutic effect is expected to differ depending on the cause, but no paper that was reviewed evaluated treatments according to the cause. Therefore, in the present evaluation, the statements are limited to treatments and their effects regardless of the cause.

## <Summary>

It was difficult to comprehensively discuss treatments, because its cause varied widely, and treatments for various causes were performed. Therefore, treatments were classified into conservative treatments (fasting, high‐calorie infusion, MCT), drug treatments (octreotide), sclerotherapy, abdominal drainage, abdominal puncture, and surgical treatment, and the effects of each treatment were evaluated.

Treatments effective for refractory chylous ascites can be summarized as follows with the understanding that they may depend on the cause and that the level of evidence of the available reports concerning treatments and their effects is low. Conservative treatments, such as fasting, high‐calorie infusion, and MCT, should be performed first because of the rareness of adverse effects. In patients who respond insufficiently to conservative treatments, drug treatments using octreotide can be considered as there have been case series and many case reports. Concerning sclerotherapy, the number of reported cases is small, and further large clinical studies will be needed to confirm its usefulness. Abdominal paracentesis and surgical treatments may be considered for chylous ascites that does not response to conservative or drug treatments for about 1 month.

Thus, the draft recommendation is “Conservative treatments, such as fasting, high‐calorie infusion, and MCT, should be performed first, and, if they are ineffective, drug treatments, sclerotherapy, and surgical treatments may be considered.” However, evaluation of this CQ by a design with a higher level of evidence, such as RCT, is considered necessary for the future.

### CQ28: What kinds of complications are associated with treatments for intra‐abdominal LM?

*Recommendation*: Complications associated with sclerotherapy for intra‐abdominal LM include bowel obstruction, hemorrhage, pain, hematuria and chylous ascites. Surgical treatment of the disease can be associated with serious complications such as occlusion of the inferior vena cava and massive resection of the intestine as well as more common, wound infection, bowel obstruction, hemorrhage and chylous ascites.

*Strength of recommendation*: No recommendation

*Evidence*: D (very weak)

Comments

*[Process of preparation of recommendation]*

Patients with intra‐abdominal LM are treated with various modalities from non‐surgical therapy to surgical procedures. Treatment modality is selected depending on the patient’s state. Therefore, it is necessary for the clinician, patient, and family to share information concerning complications that may be associated with treatments for implementing them smoothly. However, there are no resources that give a clear answer to this problem, and both clinicians and patients may become disconcerted. Therefore, the CQ “What kinds of complications are associated with treatments for intra‐abdominal LM?” was formulated, and information available at present was accumulated and integrated for the answer.

*<Literature search and screening>*

As a result of literature search, 203 papers in Japanese and 602 papers in English (593 from PubMed, 9 from Cochrane) were subjected to primary screening. Of these papers, 23 in Japanese and 27 in English were subjected to secondary screening concerning this CQ. They included no papers with a high level of evidence, such as systematic reviews or RCTs, and all of them were case series or case reports. To answer CQ 28, the results and discussion in each case series were integrated.

*<Review of observational studies (case series)>*

Complications in the CQ were evaluated by defining them as those encountered when patients with intra‐abdominal LM were treated, and reports on sclerotherapy and surgery were reviewed.

*Results of review*

1. Complications associated with sclerotherapy

Sclerotherapy using OK‐432 was reported to be associated with bowel obstruction and hemorrhage for mesenteric LM,^317^ and chylous ascites for retroperitoneal LM.^326^ Sclerotherapy using acetic acid was reported to be associated with pain and hematuria in patients with retroperitoneal LM.^318^

- Complications associated with surgical procedures

Complete resection of both mesenteric and retroperitoneal LM by laparotomy was reported to be associated with wound infection^324^, ^354^ and bowel obstruction^323^, ^354^, ^355^ as common complications. There were reports of serious complications such as occlusion of the inferior vena cava^317^ and massive resection of the intestine was necessary due to diffuse infiltration of the LM tissue to the intestinal wall.^356^

In a report^357^ about complications associated with complete laparoscopic resection of intra‐abdominal LM by Tran *et al*., resection was attempted in 47 patients, and conversion to laparotomy was necessary in 3 (6.4%) due to tight adhesion in 2 and intraoperative hemorrhage in 1.

Partial resection by laparotomy was reported to be associated with persistent ascites over a long period that was refractory to the treatment.^355^

*Limitations*: Patients with intra‐abdominal LM are treated with various modalities including sclerotherapy and surgical procedures. Modalities were combined in many cases, and complications are often reported as those of entire treatment without more detail information about those associated with individual treatment.

## <Summary>

For answering the CQ, “What kinds of complications are associated with treatments for intra‐abdominal LM?,” no literature with a high level of evidence was found, but foreseeable complications were listed from many case reports. Bowel obstruction, hemorrhage, pain, hematuria, and chylous ascites were reported as complications of sclerotherapy. Serious conditions, such as occlusion of the inferior vena cava and massive resection of the intestine, as well as common complications, such as wound infection, bowel obstruction, hemorrhage and chylous ascites were reported as complications after surgical procedures.

Although the incidences and differences in complications in respect of the site and histological type are not shown in the literature, each patient with intra‐abdominal LM should be treated with sufficient evaluation of the site, size and symptoms. In addition, treatment must be implemented with sufficient understanding of the possible complications.

Thus, we propose “Complications associated with sclerotherapy for intra‐abdominal LM include bowel obstruction, hemorrhage, pain, hematuria, and chylous ascites. Surgical treatment of the disease can be associated with serious complications such as occlusion of the inferior vena cava and massive resection of the intestine as well as more common, wound infection, bowel obstruction, hemorrhage and chylous ascites.” as a recommendation draft.

### CQ29: What treatments are effective for LM causing airway obstruction in the mediastinum?

*Recommendation*: Sclerotherapy is effective for macrocystic lesions, and surgical resection is effective for microcystic lesions. However, as the complication rate is relatively high, treatments should be selected according to the condition of each case.

*Strength of recommendation*: 2 (weak)

*Evidence*: D (very weak)

Comments

*[Process of preparation of recommendation]*

Among LM, those that may cause airway obstruction due to their sites are life‐threatening. Lesions in the mediastinum cause respiratory disorders if they physically compress the trachea or bronchi and obstruction the airway or markedly protrude into the thoracic cavity and narrow it.

In such situations, aggressive and effective treatment is necessary, but the therapeutic approach must be selected carefully in consideration of the relationship of the lesion with the important organs around it such as the large cardiac vessels, mediastinal nerve, and thoracic duct. However, the judgment is often difficult in clinical settings.

Therefore, the CQ, “What treatments are effective for LM causing airway obstruction in the mediastinum?” was formulated, and the presently available knowledge concerning matters including the risk of complications and prognosis of treatments, such as surgical resection and sclerotherapy, was summarized.

*<Literature search and screening>*

As a result of literature search, 134 papers in Japanese and 227 in English (226 from PubMed, 1 from Cochrane) were subjected to primary screening. Of these papers, 5 in Japanese and 16 in English were subjected to secondary screening concerning this CQ. Since they included none with a high level of evidence, such as a systematic review or RCT, and all were case series or case reports, the results and discussion in each case series were integrated.

*<Review of observational studies (case series)>*

By screening of the literature, the following approaches were found for the treatment of LM in the mediastinum.

Therapeutic options are surgical resection, puncture and drainage, sclerotherapy (OK‐432, bleomycin, ethiblock, anhydrous ethanol), drug treatments (Chinese herbal medicines such as *eppikajutsuto* and *ogikenchuto*), and no treatment. Of these approaches, surgical resection and sclerotherapy using OK‐432 have been evaluated in a relatively large number of cases, and reports of other therapy had extremely limited number of cases, e.g., reports of only 1 case.

*Results of review*

Boardman *et al*.^358^ reported that, of the 97 patients with LM of the head and neck region, surgical treatments were necessary in 6 of the 12 patients with mediastinal lesions, that complications of surgery occurred in 4 of the 6 patients, and that long‐term nerve damage was observed in 3 of them. In addition, they reported that management by tracheotomy was necessary in 15% of all patients. Complete or nearly complete remission was observed in 92% of the patients, but they suggested that surgical treatments should be indicated only when there is airway obstruction or there is the risk of it, because surgical treatment of mediastinal lesions frequently causes complications.

Park *et al*.^359^ reported that they surgically resected mediastinal LM in 12 patients. Seven of them had dyspnea, and 3 were asymptomatic, but they were all judged to have indications for surgery due to symptoms or the tendency of the lesions to enlarge. A total of 5 recurrences were observed in 4 patients (33%) during a mean period of 3.6 years after the initial surgery, and all underwent by re‐resection. No perioperative death was observed, and, in a total of 25 cases including past cases, the overall survival was not different compared with that in healthy individuals over a follow‐up period of 11.5 years.

Smith *et al*.^302^ performed local injection of OK‐432 in 16 patients with mediastinal LM and reported ≥60% regression of the lesion in 13 (81%). They also mentioned treatment responses according to the histological types and, by reporting responses (complete or nearly complete remission) in 94% of those with macrocystic lesions, 63% of those with mixed lesions, but 0% in those with microcystic lesions, suggested a macrocystic lesion to be a good indication for sclerotherapy using OK‐432. Although not from the viewpoint of airway obstruction, they reported that treatment using OK‐432 was more effective than surgical resection and less frequently caused serious complications.

*Limitations*

There have been no papers that directly analyzed treatments effective for mediastinal lesions expected to cause airway obstruction, and many papers reported cases of mediastinal lesions that responded to treatments. Therefore, we simply extracted matters relevant to this CQ from these reports.

## <Summary>

There was no literature with a high level of evidence concerning effective treatments for LM in the mediastinum causing airway obstruction. A few case reports that referred to the effects of surgery and sclerotherapy were observed, but it was difficult to present objective and specific figures concerning their effectiveness or safety. However, according to the available information, it should be noted that favorable responses have been obtained by OK‐432 local injection in macrocystic lesions and that complications due to surgical resection are likely to occur relatively frequently.

From these observations, we consider the following to be a therapeutic approach that can be proposed: “Sclerotherapy, such as that by local injection of OK‐432, should be considered for macrocystic lesions, and, for lesions that are technically difficult to treat by sclerotherapy or microcystic lesions, surgical resection should be considered with attention to complications. In addition, it is necessary to pay attention to the appearance of respiratory disturbances before and after these treatments and to constantly evaluate indications for securing the airway by intratracheal intubation or tracheostomy.” Therefore, at present, we recommend, “Sclerotherapy is effective for macrocystic lesions, and surgical resection is effective for microcystic lesions. However, as the complication rate is relatively high, treatments should be selected according to the condition of each case.”

### CQ30: Should sclerotherapy be performed in infancy for a patient with head and neck LM affecting the airway?

*Recommendation*: In a patient with LM around the airway, there is risk of respiratory distress in infancy, while airway obstruction is likely to be exacerbated by sclerotherapy. Particularly, when risk of airway obstruction is judged to be high or when the patient has already presented symptoms, it is proposed to perform sclerotherapy with sufficient preparations including airway management.

*Strength of recommendation*: 2 (weak)

*Evidence*: D (very weak)

Comments

*[Process of preparing recommendation]*

LM of the neck, which are located in an exposed part of the body, may cause cosmetic problems that are important, but airway obstruction can particularly be a serious problem in some cases.

Sclerotherapy, which is one of the major treatment modalities, is most effective in patients with cystic LM, but swelling of the treated portion after the therapy may cause or exacerbate airway obstruction symptoms especially in neonates. The upper airway will become less vulnerable to obstruction because it becomes less frail and wider as patients grow and respiratory distress tends to be unlikely. Therefore, it is occasionally difficult to determine how a patient who does not present any obstructive symptom should be treated in infancy.

Thus, we evaluated this problem by formulating the CQ, “Should sclerotherapy be performed in infancy for a patient with the neck LM affecting airway?”

*<Literature search and screening>*

As a result of search, 86 papers in Japanese and 135 papers in English (130 from PubMed, 5 from Cochrane) were subjected to primary screening. Of these papers, 6 in Japanese and 20 in English were subjected to secondary screening concerning this CQ. They included 1 systematic review (SR), 1 RCT, 2 prospective studies (PS) and 1 retrospective cohort study, but all the others were case series or case reports. Therefore, the results and discussion, primarily, in these SR, RCT, PS, and retrospective cohort study, but also in other case series were integrated.

*<Review of observational studies>*

The literature concerning the effectiveness of sclerotherapy for head and neck LM in infancy was reviewed from the viewpoints of responses (prognosis (survival rate or mortality), size, symptoms, and cosmetic improvement) and complications.

Sclerosing agents used as keywords for the present literature search varied widely and included OK‐432, bleomycin, ethanol, doxycycline, STS and fibrin glue. No paper evaluated differences in effectiveness of various agents due to their methods of administrations for lesions around the neck affecting airway. Therefore, differences among agents were excluded from the evaluation of this CQ.

*Results of review*

1. Responses

*A. Prognosis (survival rate or mortality)*

According to the SR by Adams *et al*.,^360^ the mortality was 4.7% in 277 cases with head and neck LM. Since lesions around the airway were not the only target, and since sclerotherapy was not the only treatment modality, the paper has not quite rightly answered to the CQ. However, since patients who died were all before one year of age and their causes of death are considered to have been mostly airway problems, such as airway obstruction and aspiration due to vocal cord paralysis in 8, and as at least 1 patient is judged to have died due to complications of invasive treatment, the paper is considered to indicate the risk of this disorder during infancy.

*B. Size*

Many of the papers that referred to the size regression evaluated it by four categories; (1) excellent or complete (≥90% regression), (2) good or substantial (≥50% and <90% regression), (3) fair or intermediate (≥20% and <50% regression), (4) and poor or none (≤20% regression).

Ravindranathan *et al*.^306^ treated 5 patients (aged 4–19 months) with cervicofacial LM by sclerotherapy using OK‐432 (in addition to Fibrovein® (STD Pharmaceutical Products Ltd, Hereford, UK) in 2) and reported that the responses were good in 1 (20%) (cystic), partial in 1 (20%) (cavernous), and poor in 3 (60%) (2 with cavernous lesions that required tracheotomy and 1 with cystic lesions in whom the condition improved to good after surgical resection). However, they did not mention the evaluation criteria for good, partial, and poor.

According to the report of 8 cases with head and neck LM by Leung *et al*.,^361^ all patients underwent sclerotherapy, ≥50% regression was observed in all patients with complete regression in 2. However, the patient age varied from 2 months to 11 years, and the types of LM were not mentioned.

Ogawa *et al*.^362^ reported 9 patients (including 5 preschoolers and toddlers, 2 school children, and 2 adults) who underwent OK‐432 sclerotherapy for the neck LM and evaluated it to be markedly effective in 8, in whom the lesions mostly disappeared, and effective in 1, who showed a ≥50% regression. Eight patients in whom the treatment was markedly effective consisted of 1 with mixed and 7 with cystic lesions, and the one in whom the treatment was effective had a mixed type.

Cahill *et al*.^315^ reported doxycycline sclerotherapy in 17 patients with head and neck LM (cystic in 10, mixed in 7 (3 required tracheotomy)), and its size regression was reported to be >90% in 7 (41.2%) (cystic in 6, mixed in 1), 75–89% in 4 (23.5%) (cystic in 2, mixed in 2), 51–74% in 4 (23.5%) (cystic in 1, mixed in 3), and 25–50% in 2 (11.8%) (mixed in 2).

Nehra *et al*.^311^ reported doxycycline sclerotherapy in 11 patients with head and neck LM (cystic in 7, mixed in 4; aged 2 days‐21 months) (later combined with surgical resection in 3). The treatment results were excellent in 5 (45.5% of all patients) and satisfactory in 2 (18.2% of all patients) among 7 patients with cystic lesions but poor in all 4 patients with mixed type lesions (36.4% of all patients). Particularly, 3 of the 4 patients with mixed type lesions required tracheal intubation shortly after birth and underwent sclerotherapy while intubated, but the effects were poor in all of them. Surgical resection was added in 1 and was under consideration in another.

*C. Symptoms*

According to Ravindranathan *et al*.^306^ who reported 5 patients with cervicofacial LM (aged 4–19 months) treated with sclerotherapy using OK‐432 (in addition to fibrovein in 2), 4 exhibited symptoms of airway obstruction before treatment. Symptoms included dysphagia in 2 and dyspnea (including croup‐like symptoms) in 4 (some both). Symptoms were alleviated by sclerotherapy in 2 out of 4 (cystic 1, cavernous 1), but tracheotomy was necessary in the remaining 2 (cavernous in both) without improvement.

In the report of 8 patients with head and neck LM and 5 patients with VM (aged 2 months‐11 years) by Leung *et al*.,^361^ their symptoms noted before treatment were mass or swelling (10 patients (77%)), pain after hemorrhage (2 patients (15%)), skin discoloration (blue) (1 patient (8%)), obstructive airway symptoms (6 patients (46%)), and swallowing difficulty (1 patient, (8%)). All symptoms were alleviated by sclerotherapy (doxycycline for LM, STS foam for VM).

Arimoto *et al*.^363^ reported a patient with cystic LM in the neck presented 3 months after birth. The patient presented with respiratory distress at the age of 10 months due to enlargement of the LM following upper respiratory infection. While left vocal cord fixation due to the mass was confirmed by ultrasonography before treatment, aspiration of the cyst and steroid administration resulted in opening of the glottic area and regression of the mass with relief of wheezing and distress. Since they underwent sclerotherapy 2 months after the disappearance of symptoms, aspiration of internal fluid and steroid administration rather than sclerotherapy were directly effective for the alleviation of symptoms.

Kitagawa *et al*.^364^ reported a patient with giant LM of the neck which had been prenatally diagnosed and was treated under ex utero intrapartum treatment (EXIT) by tracheal intubation after aspiration of the cyst. The lesion was reported to be refractive to subsequent sclerotherapy and tracheotomy was eventually needed.

Nehra *et al*.^311^ reported that, among 11 patients with head and neck LM (cystic type in 7 and mixed type of cystic + cavernous in 4; aged 2 days‐21 months), 3 out of 4 with mixed LM presented respiratory distress soon after birth and were managed by intubation, but that all were extubated after sclerotherapy using doxycycline (1–3 times, median: 1.6 times).

*D. Cosmetic improvements*

No paper has reported cosmetic results in detail. Only sporadically they mentioned about surgery for redundant skin after regression of cystic lesions by sclerotherapy.

- Complications

Complications associated with treatment for LM around the airway have been reported in many papers. They include temporary conditions caused by sclerotherapy such as fever,^292^, ^303^, ^312^, ^362^, ^365^, ^366^, ^367^, ^368^, ^369^, ^370^, ^371^ local swelling^303^, ^312^, ^365^, ^367^, ^368^, ^370^, ^371^ pain,^288^, ^312^, ^362^, ^367^, ^370^, ^371^, ^372^ hemorrhage into the cyst,^288^, ^303^, ^312^, ^368^ and infection.^288^, ^292^, ^303^, ^310^, ^312^, ^360^, ^365^, ^372^ There also reported complications as the effects of treatment for head and neck lesions such as respiratory distress due to airway obstruction,^292^, ^303^, ^306^, ^312^, ^362^, ^365^, ^366^ as well as nerve palsy.^288^, ^292^, ^312^, ^360^, ^365^

According to a systematic review about head and neck LM by Adams *et al*.,^360^ both nerve damage due to sclerotherapy and post‐therapeutic infection were reported in 1 (0.8%) out of 123 patients. Since nerve damage and infection after surgery were observed in 12 (10.2%) and 7 (5.9%) out of 118 patients, respectively, the complication rate would be lower by sclerotherapy than by surgery.

Ogawa *et al*.^362^ reported a 1‐year‐and‐5‐month‐old patient who developed airway edema after OK‐432 sclerotherapy for the cystic neck LM and necessitated tracheal intubation for 3 days and cautioned against sclerotherapy for LM around the airway in young children (particularly, those less than 2 years old).

Kudo *et al*.^369^ also reported 2 patients aged 11 months and 1 year and 11 months who were treated with OK‐432 sclerotherapy intubated in advance for fear of airway obstruction due to post‐therapeutic swelling. Tomemori *et al*.^373^ also cautioned against sclerotherapy for LM in children aged less than 2 years as in the report by Ogawa *et al*.^362^

On the other hand, Kudo *et al*.^369^ reported 2 cases whose neck LM having been enlarged rapidly after suffering from measles or upper respiratory tract infection (URTI). Arimoto *et al*.^363^ also reported a patient with cystic LM of the neck 3 months after birth, who developed dyspnea due to enlargement of the lesion after URTI at 10 months and was about to be intubated.

Regarding complications due to sclerosing agents, Cahill *et al*.^315^ reported those by doxycycline, STS and absolute ethanol. They reported delayed complications, such as Horner’s syndrome, transient left lip weakness, right facial nerve palsy, and transient left hemidiaphragm paralysis, in addition to peri‐procedural complications such as hemolytic anemia after doxycycline injection in 2 patients, hypoglycemic and metabolic acidosis in 3 neonates, transient hypotension during absolute alcohol instillation and self‐limiting skin excoriation secondary to peri‐catheter leakage of doxycycline. Other reported complications include permanent vocal cord paralysis after local ethanol injection,^374^ serious complications after OK‐432 injection such as death due to pulmonary embolism,^375^ deaths due to pulmonary complications after treatment using bleomycin^376^, ^377^ and leukocytopenia due to bleomycin.^368^

*Limitations*

There are few papers that solely analyzed LM around the cervical airway. Most papers included lesions involving not only the neck but also the craniofacial and other parts of the body and reported LM with different properties such as cystic and mixed types. In addition, definition of cavernous lesions and methods of sclerotherapy (injection techniques, number of injections) were not similar among papers, and differences in these backgrounds must be taken into consideration to evaluate the effectiveness of sclerotherapy.

## <Summary>

The CQ, “Should sclerotherapy be performed in infancy for a patient with head and neck LM affecting the airway?”, was evaluated from the viewpoints of responses (prognosis (survival rate or mortality), decrease in size, symptoms, cosmetic improvements) and complications. Since there have been some reports on the risk of respiratory distress due to LM around the airway in infants, and therapeutic intervention is necessary even in infants when the risk is high or they have already developed symptoms. Such intervention is made by sclerotherapy or surgery, and as surgical resection is associated with the risk of more serious complications than sclerotherapy, intervention by less invasive sclerotherapy is recommended. Sclerotherapy is considered to be very effective because of high regression rate of the lesion and symptom/function‐improving effect. However, its effect varies depending on the disease type, somewhat less effective in the cavernous and mixed types than in the cystic type. Furthermore, when it was performed for the lesions around the airway, it may be associated with the risk of exacerbation of airway obstruction symptoms due to reactive enlargement of the lesion. Thus, we formulated the recommendation, “In LM around the airway, there is the risk of respiratory disturbances from infancy, but airway obstruction is likely to be exacerbated by sclerotherapy. Particularly, when the risk of airway obstruction is judged to be high or when symptoms have appeared, it is proposed to perform sclerotherapy with sufficient preparations including securing the airway.”

### CQ31: Is surgical resection effective for LM of the tongue?

*Recommendation*: Surgical resection is effective for reducing the size of the lesion and alleviating symptoms and functional impairment. However, total resection is often difficult, and careful decision is required in consideration of the possibility of complications and recurrence.

*Strength of recommendation*: 2 (weak)

*Evidence*: D (very weak)

Comments

*[Process of preparation of recommendation]*

While the tongue is one of the frequent sites of LM, the lesion is often distributed widely over the neck rather than is localized in the tongue. LM of the tongue not only cause cosmetic problems, such as protrusion from the mouth and bleeding, but also readily occupy the oropharyngeal cavity and cause functional problems such as disorder of mouth closing, difficulty in speaking, respiratory disturbances, and impairment of oral food intake. These conditions are treated at departments including plastic surgery, oral surgery, otorhinolaryngology, and pediatric surgery. LM of the tongue are treated by surgical resection or sclerotherapy, but comprehensive evaluation of the condition of individual cases including the distribution of the lesion in the tongue, involvement of other areas and cyst components, and vascular distribution in addition to general information, such as the risk of complications and recurrence in each treatment, is necessary.

Therefore, the CQ, “Is surgical resection effective for LM of the tongue?” was formulated, and the present knowledge about the effectiveness of surgical resection of the lesion, particularly, by partial glossectomy was summarized.

*<Literature search and screening>*

As a result of search, 29 papers in Japanese and 76 papers in English (75 from PubMed, 1 from Cochrane) were subjected to primary screening. Of these papers, 2 in Japanese and 10 in English were subjected to secondary screening concerning this CQ. They included 1 retrospective cohort study, but most other papers were case series or case reports. Consequently, in the evaluation of this CQ, the results and discussion of the cohort study and each case series were integrated.

*<Review of observational studies (case series)>*

The effectiveness of surgical resection of LM of the tongue was evaluated from the viewpoints of resectability of the lesion, symptoms, function, and cosmetic improvements as elements of responses as well as complications and recurrence.

*Results of review*

1. Responses

*A. Resectability of the lesion*

Twenty‐four cases of tongue lesions treated by surgical resection alone were reported in 4 papers. Catalfamo *et al*.^378^ performed surgical resection of localized masses including normal structures with a margin of 1 cm in the horizontal direction and reported that the size of tongue lesions was reduced in 8 (88.9%) of the 9 patients.

Concerning large lesions impossible to resect totally, Boardman *et al*.^358^ reported 13 cases of partial surgical resection, but several operations were often necessary to reduce lesion size. A total of 2 case have been reported,^379^, ^380^ and the lesion size was reduced in both. Although differences were observed in re‐enlargement after surgery, they are discussed in detail in “(2) Complications”.

In 1 case report, sclerotherapy was performed 15 times, but the lesion size could not be reduced, and surgical resection was performed, eventually resulting in a favorable outcome without recurrence.^381^

According to a report of 89 cases of head and neck LM by Lei *et al*.,^291^ the outcome was excellent in 73 (82%) and good in 16 (18%) although it was not a report of cases of tongue lesions alone. They included 43 cases of tongue lesions.

In addition, a few papers^382^, ^383^, ^384^, ^385^ that suggested the effectiveness of combinations of surgical resection with sclerotherapy and laser therapy were observed. Wiegand *et al*.^383^ classified the disease into 4 stages according to the area of involvement and reported that the stage can be a prognostic factor. Surgery was effective, and complications were rare, when the lesion was localized in the superficial layer and part of the muscle layer. Surgical resection can also be effective, but complete resection is difficult, when the lesion extends over the entire muscle layer or to the tongue base and neck. Therefore, partial resection is often repeated and combined with laser therapy and sclerotherapy, but the recurrence is observed very frequently, and the results did not contradict the reports mentioned below in the section of the recurrence rate.^291^, ^358^

*B. Symptoms*

A wide variety of symptoms have been reported depending on the site of the mass, and they include tongue discomfort, bleeding, pain, and difficulty in oral feeding.^386^ Roy *et al*.^387^ reported that bleeding from the tongue surface, pain, and eating difficulty were alleviated by cauterization.

*C. Functions*

In most patients who exhibited functional impairment, the lesions were so extended that they were no longer indications for one‐time surgical resection. Large masses located at sites such as the tongue base cause respiratory disturbances, swallowing disorders, and difficulty in speech. According to the report by Azizkhan *et al*.,^385^ oral intake of normally cooked food became possible in 14, and normal vocalization became possible in 8, of the 21 patients with tongue base lesions. In addition, 5 of the 17 patients who needed tracheotomy were weaned.

*D. Cosmetic improvements*

Objective evaluation of cosmetic effects is also difficult.

Azizkhan *et al*.^385^ reported that, of the 20 patients, excluding 1 with severe deformity who died, deformity of structures around the tongue, such as the mandible and maxilla, was mild in 6, moderate in 5, and severe in 9. There have been a few reports that cosmetic improvements were also observed in patients who showed a reduction of the tongue size by surgical resection, but objective evaluation is insufficient.

- Complications

Although the properties of the lesions are unclear in some papers, facial nerve paralysis, vagus nerve paralysis, infection, hematoma, seroma, salivary leakage, ruptured suture, and skin flap necrosis have been reported as complications of the facial region. There have also been reports of temporary complications such as pain and hemorrhage.

- Recurrence

There have been a few postoperative evaluations reporting that no reactivation that clinically required treatment was observed. Lei *et al*.^291^ reported greater details: Recurrence was observed in 21 (23.6%) of 89 patients and was more frequent in those aged less than 1 year, those with lesions in the oral cavity/face, those with lesions at 3 or more sites, and those with microcystic lesions. According to Boardman *et al*.,^358^ LM of the tongue recurred in 12 (48%) of 28 patients, more often than other head and neck lesions. As factors related to this more frequent recurrence of lingual LM, more frequent involvement of other regions, such as the floor of mouth, and a high percentage of microcystic lesions (70%) have been suggested. Of the 2 patients treated by surgical resection alone, 1 who underwent resection of the middle part of the tongue showed no re‐enlargement for 1 year or longer after surgery,^379^ but surgery was repeated 3 times in the 1 who underwent marginal resection.^380^ This patient who underwent repeated resections also showed no re‐enlargement although the time of the last resection is unclear.

*Limitations*

In some papers, surgical resection was combined with other treatments,^381^, ^382^, ^383^, ^384^, ^385^, ^387^ lesions in other areas such as the neck were included,^291^ and the lesion types were unknown. The lack of standardization of subjects and uniformity of the definition or time of recurrence must be considered in the evaluation of the effectiveness of surgical resection.

## <Summary>

Many papers suggest that surgical resection is effective for reducing the size of lingual LM. However, in patients with large lesions, lesions extending to structures other than the tongue, and microcystic lesions, several resections or combination of resection with other treatments such as sclerotherapy and laser therapy were necessary, and the recurrence rate tended to be higher. While a few papers referred to symptoms, functional outcome, and cosmetic improvements, none showed a high level of evidence, and the evidence was insufficient for general discussion of the effectiveness of surgical resection.

Therefore, concerning the effectiveness of surgical resection for LM of the tongue, “Surgical resection is effective for reducing the size of the lesion and alleviating symptoms and functional impairment. However, total resection is often difficult depending on the distribution of the lesion, and careful decision is required in consideration of the possibility of complications and recurrence.” was proposed as a draft recommendation.

### CQ32: Is aggressive surgical intervention effective for chylous pleural effusion in the neonatal period?

*Recommendation*: For chylous pleural effusion refractory to conservative treatments, surgical procedures, such as pleurodesis, ligation of the thoracic duct, and pleuroperitoneal shunting, may be effective.

*Strength of recommendation*: 2 (weak)

*Evidence*: D (very weak)

Comments

*[Process of determining recommendation]*

Primary chylous pleural effusion during the neonatal period is often refractory and can be fatal. Thoracic drainage is performed for respiratory insufficiency due to accumulation of pleural effusion, followed by conservative treatments, such as nutritional therapy, steroid, and octreotide therapy, conducted primarily by neonatologist until resolution of chylous pleural effusion.

In refractory cases that do not respond to these conservative therapies, surgical intervention, such as ligation of the thoracic duct and pleurodesis, may be performed. However, no sufficient consensus has been obtained concerning their effects. To evaluate problems, such as at what point surgical intervention should be made and whether aggressive surgical intervention is effective for such a condition, the CQ, “Is aggressive surgical intervention effective for chylous pleural effusion in the neonatal period?”, was formulated, and the knowledge available at present was summarized.

*<Literature search and screening>*

As a result of search, 98 papers in Japanese and 264 papers in English (262 from PubMed, 2 from Cochrane) were subjected to primary screening. Of these papers, 8 in Japanese and 9 in English were subjected to secondary screening concerning this CQ. They included none with a high level of evidence, such as a systematic review or RCT that evaluated surgical treatment, and all papers were case series or case reports. Consequently, the results and discussion in each of the case series judged to be useful for the preparation of the draft recommendation were integrated although they were weak as the evidence for the evaluation of this CQ.

*<Review of observational studies (case series)>*

The literature concerning the effectiveness of surgical treatment for chylous pleural effusion in the neonatal period was reviewed from the viewpoints of responses and complications.

*Results of review*

1. Responses

Surgical treatment for neonatal chylothorax is performed in patients who respond insufficiently even to thoracic drainage in addition to nutritional therapy using MCT milk or total parenteral nutrition or drug treatment such as octreotide administration.

The methods for surgical intervention found by the present literature review included ligation of the thoracic duct and pleuroperitoneal shunting as well as pleurodesis with OK‐432 administration, intrathoracic infusion of fibrin, and povidone‐iodine administration, and some patients diagnosed in utero underwent pleuro‐amniotic shunting. Cases in which mildly invasive treatments, such as thoracoscopic ligation of the thoracic duct and intrathoracic fibrin application, have been reported in addition to those who underwent thoracic duct ligation by thoracotomy.

Treatments that were performed before surgery and their periods were not uniform. In addition, since there are cases that developed chylous pleural effusion after surgery and those of congenital chylothorax, the diversity of the patient background must be taken into consideration in the efficacy evaluation.

Among the surgically treated cases, those in whom chylous plural effusion disappeared, respiratory symptoms were alleviated, and weaning from the respirator became possible have been reported.^388^, ^389^ In addition, the absence of recurrence or reactivation is considered to be a point.^388^, ^389^, ^390^, ^391^ There were reports that chylous pleural effusion after thoracic surgery was resolved by drainage alone. Cleveland *et al*.^392^ considered conservative treatments, such as total parenteral nutrition, octreotide, and diuretic administration, to be the best and, observing that, of the poor responders, the mortality was 80% in 5 who continued to be managed by conservative treatments but 0% in 4 who underwent additional surgery, reported that surgical treatment contributed to the reduction of the mortality. According to the guidelines for the treatment of chylous thoracic effusion by Buttiker *et al*.,^393^ conservative treatment should be continuing for about 3 weeks but should be abandoned thereafter because of the risk of nutritional disturbance, increased susceptibility to infection, and liver disorders. However, Kaji *et al*.^394^ reported that it is difficult to set a clear period of conservative therapy, because the effectiveness and success rate of surgical treatment are unclear.

- Complications

As complications due to sclerosing agents, fever and increased inflammatory reaction due to the administration of OK‐432 as well as pulmonary abscess and temporary flaccidity and protrusion of the upper abdominal region considered to have been due to intercostal nerve damage have been reported. While chyle leakage in the abdominal cavity was noted in a patient who underwent pleuroperitoneal shunting, there were no reports of fatal complications.

*Limitations*

Surgical treatment was performed in most reported cases when responses to conservative therapy were not obtained. Therefore, it must be assumed that the results of evaluation of this CQ are based on data concerning the effectiveness of surgery performed with conservative therapy.

## <Summary>

The literature was reviewed concerning the effectiveness of aggressive surgical intervention for neonatal chylous thoracic effusion from the viewpoints of responses and complications, but no objective study with a high level of evidence was found. In most reported cases, surgical treatment was performed when responses to conservative treatments were poor. Therefore, it is difficult to compare surgery with other therapies, and the evaluation of the period of conservative treatment before surgery remains insufficient. However, there was a paper that proposed surgical intervention after attempting conservative treatments for 3 weeks as a standard.

Thus, surgical intervention for neonatal chylous pleural effusion is characterized at present as an approach that may be effective but should be evaluated when the condition is not improved by other treatments, and “Surgical procedures, such as pleurodesis, ligation of the thoracic duct, and pleuroperitoneal shunting, may be effective for chylous pleural effusion refractory to conservative treatments.” is proposed as a draft recommendation.

### CQ33: What treatments are effective for refractory chylous pleural, and pericardial effusion and respiratory disturbances of the patients with generalized lymphatic anomaly (GLA) and Gorham‐Stout disease (GSD)?

*Recommendation*: While treatments including surgery, sclerotherapy, radiotherapy, nutritional therapy, and drug therapy are conducted, there is presently no effective treatment with a high level of evidence. Treatments should be selected in consideration of complications and adverse effects according to individual symptoms.

*Strength of recommendation*: 2 (weak)

*Evidence*: D (very weak)

Comments

*[Process of preparation of recommendation]*

GLA and GSD are refractory diseases that cause a wide variety of symptoms in the entire body and are difficult to diagnose and treat. The investigation by the Health and Labour Sciences Research group (Ozeki group) carried out by 2013 showed that the mortality is particularly high when the patients had thoracic lesions.

Among the thoracic symptoms, chylous pleural effusion/pericardial effusion are often refractory and occasionally fatal. While information about the disease is extremely limited because of its rareness, case reports are being globally accumulated as chronic cases are managed on an outpatient basis, and as severe cases are treated intensively.

Presently, no radical treatment for these refractory diseases is known, but the CQ, “What treatments are effective for refractory chylous pleural, and pericardial effusion and respiratory disturbances of the patients with GLA and GSD?” was formulated to compile the knowledge about what treatments are effective as it is a problem of clinical importance.

*<Literature search and screening>*

As a result of search, 208 papers in Japanese and 617 papers in English (598 from PubMed, 19 from Cochrane) were subjected to primary screening. Of these papers, 2 in Japanese and 25 in English were subjected to secondary screening concerning CQ 37. They included no studies with a high level of evidence, such as a systematic review and RCT, and all were reports of 1–2 cases. Therefore, the evaluation of this CQ was performed by integrating the results and discussion in case series judged to be useful for the preparation of the draft recommendation despite the lack of evidence.

*<Review of observational studies (case series)>*

The effectiveness of various treatments for refractory GLA and GSD was evaluated according to the prognosis and the presence or absence of improvement in imaging findings, improvement in symptoms, improvement in airway obstruction, enlargement of the lesion, regression, treatment‐related complications, recurrence, and reactivation.

*Conditions of patients*

The cause of chylous pleural and pericardial effusion is lymphorrhea from lymphatic vessel tissue lesions that have primarily invaded the mediastinum and pleura, and lymphorrhea from osteolytic lesions of the ribs and vertebrae was also observed. Respiratory disturbances were caused by pleural effusion, chylous pleural effusion, pericardial effusion, and direct invasion of the mediastinum and lungs.

*Results of review*

As surgical treatments for chylous pleural effusion, procedures, such as thoracentesis, thoracic drainage, ligation of the thoracic duct, and pleural decortication, have been performed, and local lesions were surgically resected. In most cases, thoracentesis and thoracic drainage were performed, but chyle leakage was not resolved. As for complications, there were patients who developed hypovolemic shock and required blood transfusion and catecholamine administration or supplementation of albumin, immunoglobulin, and clotting factors.^395^, ^396^, ^397^ While chylous pleural effusion was controlled in some patients who underwent ligation of the thoracic duct,^397^, ^398^, ^399^, ^400^, ^401^, ^402^, ^403^, ^404^, ^405^, ^406^, ^407^, ^408^ the treatment was performed in combination with other surgical procedure or radiotherapy in all cases.^400^, ^402^, ^408^ There was 1 case that showed improvement in respiratory disturbance.^406^ As complications of ligation of the thoracic duct, splenomegaly and lymphorrhea^405^ and left‐sided pleural effusion^397^, ^405^ have been reported. In the patients who showed marked improvements in chylous pleural effusion^395^, ^405^, ^408^ after pleural decortication,^395^, ^396^, ^401^, ^403^, ^404^, ^405^, ^408^, ^409^ the procedure was performed in combination with other surgical treatments or sclerotherapy, and there was no mention about complications. There were cases that showed marked improvements in chylous pleural effusion^396^, ^400^, ^405^, ^408^ among those who underwent surgical resection of local lesions including splenectomy,^396^, ^397^, ^400^, ^405^, ^408^, ^410^, ^411^, ^412^ but the procedure was performed in combination with other surgical treatments in most of them. Hemorrhage was reported as a complication.^410^ Among other treatments, pleuroperitoneal shunting^403^ and lung transplantation^413^ were performed, and alleviation of respiratory disturbance was noted in the patient who underwent lung transplantation.

As a surgical treatment for pericardial effusion, pericardiocentesis was performed,^396^, ^414^, ^415^, ^416^ and pericardial fenestration was performed when pericardial effusion could not be controlled by pericardiocentesis.^396^, ^416^ There was no mention about complications.

As sclerotherapy, pleurodesis was performed using OK‐432, talc, and minocycline.^395^, ^397^, ^398^, ^399^, ^404^, ^408^, ^411^, ^416^, ^417^, ^418^ There were patients who responded markedly to sclerotherapy alone and sclerotherapy combined with surgical procedures such as pleural decortication or local radiotherapy. There was no mention about complications of sclerotherapy.

There have also been reports^399^, ^400^, ^402^, ^403^, ^404^, ^410^, ^411^, ^412^, ^415^, ^416^, ^418^, ^419^, ^420^ on local (e.g., lesion area, thoracic duct region) and thoracic radiotherapy for chylous pleural effusion and local lesions, and marked responses of chylous pleural effusion and responses of respiratory symptoms were noted, but other treatments were performed concomitantly in some patients. Radiation pneumonitis has been reported as a complication.^416^

Concerning nutritional therapy, fasting, high‐calorie infusion, and MCT diet have been performed alone or in combinations, but few cases that showed alleviation of chylous pleural effusion were observed.^395^, ^396^, ^398^, ^399^, ^400^, ^403^, ^405^, ^408^, ^421^

For drug therapy against chylous pleural effusion, drugs including interferon α, propranolol, anticancer agents (e.g., vincristine), bisphosphonate, octreotide, steroid, sirolimus, and low‐molecular‐weight heparin were used. Interferon α was used most frequently,^395^, ^396^, ^397^, ^398^, ^400^, ^401^, ^403^, ^415^, ^421^ and marked improvement in chylothorax was reported in 5 cases. Of these cases, interferon α was used with propranolol in 1^395^ and with low‐molecular‐weight heparin and local radiotherapy (15 Gy) in 1.^400^ As for complications of drug therapy using interferon α, there were reports of fever, nausea, and headache^421^ and thrombocytopenia and hepatic toxicity.^397^ There was no report of improvement in chylous pleural effusion by the use of steroid^395^, ^399^, ^403^, ^415^ or octreotide^395^, ^397^, ^398^, ^400^, ^403^, ^405^ alone. Concerning other drug therapies, only a few cases have been reported with no improvement in chylous pleural effusion. One case that showed regression of mediastinal invasion of GLA and alleviation of respiratory disturbance by sirolimus treatment has been reported,^414^ and hypertension was noted as a complication. In drug therapy for pericardial effusion, diuretics were used for conservative therapy.^400^

*Limitations*

Although cases that responded to various therapies have been reported, treatments are often performed in combinations, and the evaluation of the effectiveness of each treatment alone is difficult at this point.

## <Summary>

Treatments effective for GLA and GSD presenting with refractory chylous pleural effusion, pericardial effusion, and respiratory disturbances were evaluated by a review of the literature, which was primarily case reports. Various treatments, such as surgery, sclerotherapy, radiotherapy, nutritional therapy, and drug therapy, have been performed, but, there was no study with a sufficient number of cases and a high level of evidence because of the rareness of the disease and diversity of symptoms. Although cases that responded to various treatments have been reported, treatments are frequently performed in combinations, and the evaluation of the effectiveness of individual therapies is difficult at present. Sirolimus (a mTOR inhibitor) is considered promising as a drug for this disease, and some clinical trials are currently under way in Japan and abroad.

In clinical situations, these diseases are not recognized as indications of various drug therapies by the Japanese health insurance system, and the therapeutic effects of other treatments are also uncertain. Therefore, the above treatments cannot be recommended, but we propose that treatments “should be selected in consideration of complications and adverse effects according to individual symptoms.” It is necessary to evaluate the invasiveness, complications, and adverse effects, and select the treatments judged to be appropriate for each case.

## Conclusion

The practice guidelines for vascular anomalies have been prepared as the evidence‐based guidelines for the management of vascular anomalies.

## Conflict of interest

Conflict of interest of the guidelines preparation organization was managed by the Guidelines Executive Committee. The following corporations were disclosed by self‐declaration of the Guidelines Committee members in the 3 year‐period before 1 April 2017. Japan Pharmaceuticals and Medical Devices Agency (PMDA), Mitsubishi Foundation, Rohto Pharmaceutical Co., Ltd., Mitsubishi Tanabe Pharma Corporation, and Shionogi & Co., Ltd.

## Funding

The funding for preparation of the Japanese Clinical Practice Guidelines for Vascular Anomalies 2017 was from Health, Labour and Welfare Sciences Research Grants (Research on Policy Planning and Evaluation for Rare and Intractable Diseases) provided to ‘Japanese Research Committees for Intractable Vascular Anomalies’ (main funding source), ‘Japanese Study Group for Intractable Diseases of Pediatric Gastrointestinal Tract’, and ‘Japanese Research Committees for Survey and Establishment of Guidelines for Pediatric Respiratory Dysplastic/hypoplastic Disease’. No financial support was received from any other organization or corporation.

## References

[1] ISSVA Classification of Vascular Anomalies ©2014 International Society for the Study of Vascular Anomalies. [Cited 2014 April.] Available from URL: http://issva.org/classification

[2] Wassef M , Blei F , Adams D *et al* Vascular anomalies classification: recommendations from the International Society for the Study of Vascular Anomalies. Pediatrics 2015; 136: e203–14. 26055853 10.1542/peds.2014-3673

[3] [Clinical practice guidelines for vascular anomalies 2013] . The Research Committee of Intractable Vascular Anomalies, Research on Measures for Intractable Diseases, Health, Labour and Welfare Sciences Research Grants, the Ministry of Health, Labour and Welfare, Japan. [Cited 2013 March 29.] Available from URL: http://www.marianna-u.ac.jp/va/files/vascular%2520anomalies%2520practice%2520guideline%25202013.pdf. Japanese.

[4] Fukui T , Yamaguchi N (editorial supervisors), Morizane T , Yoshida M , Kojimahara N (eds). Minds Handbook for Clinical Practice Guideline Development 2014. Ver 1.0. Tokyo: Minds Guideline Center, Japan Council for Quality Health Care [Cited 2015 February 1.] Available from URL: http://minds4.jcqhc.or.jp/minds/guideline/pdf/MindsHB2014.pdf

[5] Morizane T , Yoshida M (eds). [Minds Manual for Guideline Development Ver. 1.0]. Tokyo: Japan Council for Quality Health Care, [Cited 2014 March 31]. Japanese.

[6] Kojimahara N , Nakayama T , Morizane T , Yamaguchi N , Yoshida M (eds). [Minds Manual for Guideline Development Ver. 2.0]. Tokyo: Japan Council for Quality Health Care, [Cited 2016 March 15]. Available from URL: http://minds4.jcqhc.or.jp/minds/guideline/pdf/manual_all_2.0.pdf. Japanese.

[7] Balshem H , Helfand M , Schunemann HJ *et al.* GRADE guidelines: 3. Rating the quality of evidence. J. Clin. Epidemiol. 2011; 64: 401–6. 21208779 10.1016/j.jclinepi.2010.07.015

[8] Andrews J , Guyatt G , Oxman AD *et al.* GRADE guidelines: 14. Going from evidence to recommendations: the significance and presentation of recommendations. J. Clin. Epidemiol. 2013; 66: 719–25. 23312392 10.1016/j.jclinepi.2012.03.013

[9] Liu AS , Mulliken JB , Zurakowski D , Fishman SJ , Greene AK . Extracranial arteriovenous malformations: natural progression and recurrence after treatment. Plast. Reconstr. Surg. 2010; 125: 1185–94. 20335868 10.1097/PRS.0b013e3181d18070

[10] Kohout MP , Hansen M , Pribaz JJ , Mulliken JB . Arteriovenous malformations of the head and neck: natural history and management. Plast. Reconstr. Surg. 1998; 102: 643–54. 9727427 10.1097/00006534-199809030-00006

[11] Richter GT , Suen J , North PE , James CA , Waner M , Buckmiller LM . Arteriovenous malformations of the tongue: a spectrum of disease. Laryngoscope 2007; 117: 328–35. 17277629 10.1097/01.mlg.0000249954.77551.98

[12] Hyun D , Do YS , Park KB *et al* Ethanol embolotherapy of foot arteriovenous malformations. J. Vasc. Surg. 2013; 58: 1619–26. 24280325 10.1016/j.jvs.2013.06.074

[13] Park KB , Do YS , Kim DI *et al* Predictive factors for response of peripheral arteriovenous malformations to embolization therapy: analysis of clinical data and imaging findings. J. Vasc. Interv. Radiol. 2012; 23: 1478–86. 23101921 10.1016/j.jvir.2012.08.012

[14] Wu JK , Bisdorff A , Gelbert F , Enjolras O , Burrows PE , Mulliken JB . Auricular arteriovenous malformation: evaluation, management, and outcome. Plast. Reconstr. Surg. 2005; 115: 985–95. 15793434 10.1097/01.prs.0000154207.87313.de

[15] DesPrez JD , Kiehn CL , Vlastou C , Bonstelle C . Congenital arteriovenous malformation of the head and neck. Am. J. Surg. 1978; 136: 424–9. 707720 10.1016/0002-9610(78)90255-6

[16] Dompmartin A , Labbé D , Barrellier MT , Théron J . Use of a regulating flap in the treatment of a large arteriovenous malformation of the scalp. Br. J. Plast. Surg. 1998; 51: 561–3. 9924413 10.1054/bjps.1998.0027

[17] Yamamoto Y , Ohura T , Minakawa H *et al* Experience with arteriovenous malformations treated with flap coverage. Plast. Reconstr. Surg. 1994; 94: 476–82. 8047599 10.1097/00006534-199409000-00009

[18] Hartzell LD , Stack BC Jr , Yuen J , Vural E , Suen JY . Free tissue reconstruction following excision of head and neck arteriovenous malformations. Arch. Facial Plast. Surg. 2009; 11: 171–7. 19451451 10.1001/archfacial.2009.6

[19] Visser A , FitzJohn T , Tan ST . Surgical management of arteriovenous malformation. J. Plast. Reconstr. Aesthet. Surg. 2011; 64: 283–91. 20663728 10.1016/j.bjps.2010.05.033

[20] Hong JP , Choi JW , Chang H , Lee TJ . Reconstruction of the face after resection of arteriovenous malformations using anterolateral thigh perforator flap. J. Craniofac. Surg. 2005; 16: 851–5. 16192868 10.1097/01.scs.0000187693.36765.38

[21] Koshima I , Takahashi Y , Namba Y *et al* Treatment for arteriovenous malformation. Keisei Geka 2001; 44: 665–73. Japanese.

[22] Toh S , Tsubo K , Arai H , Harata S . Vascularized free flaps for reconstruction after resection of congenital arteriovenous malformations of the hand. J. Reconstr. Microsurg. 2000; 16: 511–7. 11083389 10.1055/s-2000-8388

[23] Yokoo K , Nishihori K , Kono A , Ishiguchi T , Ohta T . [Surgical treatment of arteriovenous malformations in the head and neck combined with antecedent embolization]. Keisei Geka 2009; 52: 1201–8. Japanese.

[24] Koshima I , Nanba Y , Tsutsui T , Takahashi Y , Watanabe A , Ishii R . Free perforator flap for the treatment of defects after resection of huge arteriovenous malformations in the head and neck regions. Ann. Plast. Surg. 2003; 51: 194–9. 12897525 10.1097/01.SAP.0000044706.58478.73

[25] Kajitani N , Ikuta Y , Ishida O , Mochizuki Y , Kimori K . [Surgical treatment of arteriovenous fistula of the hand]. Nihon Te No Geka Gakkai Zasshi 1999; 15: 758–61. Japanese.

[26] Minami A , Kato H , Hirachi K . Complete removal plus dorsalis pedis flap for arteriovenous malformation in the hypothenar region. J. Reconstr. Microsurg. 1998; 14: 439–43. 9819088 10.1055/s-2007-1000204

[27] Koshima I , Soeda S , Murashita T . Extended wrap‐around flap for reconstruction of the finger with recurrent arteriovenous malformation. Plast. Reconstr. Surg. 1993; 91: 1140–4. 8479981 10.1097/00006534-199305000-00026

[28] Wojcicki P , Wojcicka K . The treatment of extensive arteriovenous malformations in the head. Pol. Przegl. Chir. 2013; 85: 83–9. 23585207 10.2478/pjs-2013-0015

[29] Ermer MA , Gutwald R , Schumacher M , Schmelzeisen R , Taschner C . Use of the radial forearm artery for secondary embolization of an extensive life‐threatening arteriovenous malformation of the mid‐face and anterior skull base – a case report. J. Craniomaxillofac. Surg. 2013; 41: 258–64. 23245682 10.1016/j.jcms.2012.10.005

[30] Ueda K , Oba S , Nakai K , Okada M , Kurokawa N , Nuri T . Functional reconstruction of the upper and lower lips and commissure with a forearm flap combined with a free gracilis muscle transfer. J. Plast. Reconstr. Aesthet. Surg. 2009; 62: e337–40. 18676215 10.1016/j.bjps.2008.01.040

[31] Ninkovic M , Sucur D , Starovic B , Markovic S . Arteriovenous fistulae after free flap surgery in a replanted hand. J. Hand. Surg. Br. 1992; 17: 657–9. 1484248 10.1016/0266-7681(92)90194-7

[32] Bit N , Vidyasagaran T , Amalorpavanathan J , Balakrishnan TM , Sritharan N . Management of a challenging arteriovenous malformation of the scalp and orbit in a patient with polycystic kidney disease. Ann. Vasc. Surg. 2012; 26: 1129.e9–29.e11. 10.1016/j.avsg.2012.04.010 22981016

[33] Righi PD , Bade MA , Coleman JJ 3rd , Allen M . Arteriovenous malformation of the base of tongue: case report and literature review. Microsurgery 1996; 17: 706–9. 9588716 10.1002/(SICI)1098-2752(1996)17:12<706::AID-MICR8>3.0.CO;2-K

[34] Minagawa T , Itaya Y , Furukawa H . Resection of an arteriovenous malformation of the scalp using a modified tumescent technique. Nihon Keisei Geka Gakkai Kaishi 2010; 30: 87–9.

[35] Suyama Y , Nakayama B , Fukuoka K *et al* Auricular reconstruction with a free radial forearm flap for necrotized ear after sclerotherapy for arteriovenous malformation: a case report. Nihon Maikuro Sajari Gakkai Kaishi 2010; 23: 311–5. Japanese.

[36] Urayama H , Harada T , Kawase H , Watanabe Y . Surgical management of soft tissue arteriovenous malformations and hemangiomas. Shoni Geka 1993; 25: 415–9. Japanese.

[37] Yamamoto Y , Sugihara T , Minakawa H , Ohkubo Y , Hayashi T . [Surgical treatment of massive arteriovenous malformation with application of hypothermia and cardiopulmonary bypass]. Nihon Keisei Geka Gakkai Kaishi 1996; 16: 863–71. Japanese.

[38] Hormozi AK , Shafii MR . Supraclavicular flap: reconstructive strategy for massive facial arteriovenous malformations. J. Craniofac. Surg. 2011; 22: 931–6. 21558921 10.1097/SCS.0b013e31820fe191

[39] Hurwitz DJ , Kerber CW . Hemodynamic considerations in the treatment of arteriovenous malformations of the face and scalp. Plast. Reconstr. Surg. 1981; 67: 421–34. 7208684 10.1097/00006534-198104000-00001

[40] Kiyokawa K , Takagi M , Fukushima J , Kizuka Y , Inoue Y , Tai Y . Surgical treatment following huge arteriovenous malformation extending from the lower lip to the chin: combination of embolization, total resection, and a double cross lip flap. J. Craniofac. Surg. 2005; 16: 443–8. 15915112 10.1097/01.scs.0000148044.80745.b3

[41] Thomas WO . Facial arteriovenous malformation managed with ablative surgery and dual rotational flap reconstruction. South Med. J. 1994; 87: 1178–82. 7973912 10.1097/00007611-199411000-00028

[42] Warwick DJ , Milling MA . Growth of a vascular malformation into a cross‐finger flap. Br. J. Clin. Pract. 1993; 47: 48. 7681687

[43] Agir H , Sen C , Onyedi M . Extended lateral supramalleolar flap for very distal foot coverage: a case with arteriovenous malformation. J. Foot Ankle Surg. 2007; 46: 310–3. 17586448 10.1053/j.jfas.2007.02.004

[44] Sakurai H , Nozaki M , Sasaki K *et al* Successful management of a giant arteriovenous fistula with a combination of selective embolization and excision: report of a case. Surg. Today 2002; 32: 189–93. 11998954 10.1007/s005950200019

[45] Watanabe T , Asato H , Umekawa K , Nomura H , Suzuki Y . [Resurfacing the index finger after resection of an arteriovenous malformation using a reverse forearm flap combined with additional venous anastomosis: a case report]. Nihon Keisei Geka Gakkai Kaishi 2012; 32: 335–9. Japanese.

[46] Ishisaka T , Naitoh H , Akiyama K , Shigeyoshi N . [A case of lower lip arteriovenous malformation with verrucous carcinoma]. Nihon Keisei Geka Gakkai Kaishi 2009; 29: 7–11. Japanese.

[47] Kitagawa S , Kizaki K , Yajima H , Mii Y , Tamai S . [Treatment of hemangiomas associated with arteriovenous fistulae in a family]. Chubu Nihon Seikei Geka Saigai Geka Gakkai Zasshi 1997; 40: 331–2. Japanese.

[48] Gunji H , Suda K , Ono I , Ariga T , Kaneko F . [Arteriovenous fistula of auricle treated with a temporoparietal fascial flap: a case report]. Nihon Keisei Geka Gakkai Kaishi 1993; 13: 221–7. Japanese.

[49] Fujita A , Asada M , Saitoh M *et al* A case of congenital arteriovenous malformation of the scalp treated with rotation flap. No Shinkei Geka Janaru 2000; 9: 86–91. Japanese.

[50] Nakamura E , Suzuki S , Imagawa K , Akamatsu T , Miyasaka M . Experience of two patients with auricular arteriovenous malformation. Skin Surg. 2014; 23: 73–8. Japanese.

[51] Yoshimura Y , Mizuno M , Kobayashi T *et al* [Case reports: extracranial arteriovenous malformation]. Hifuka No Rinsho 2014; 56: 1180–3. Japanese.

[52] Matsuzaki K , Nakamura T , Tahara T , Kashiwa H , Oshima H , Souzumi T . [Congenital arteriovenous malformation of the auricle]. Jibi Inkoka Tokeibu Geka 1995; 67: 337–41. Japanese.

[53] Schultz RC , Hermosillo CX . Congenital arteriovenous malformation of the face and scalp. Plast. Reconstr. Surg. 1980; 65: 496–501. 7360819 10.1097/00006534-198004000-00018

[54] Slaba S , Herbreteau D , Jhaveri HS *et al* Therapeutic approach to arteriovenous malformations of the tongue. Eur. Radiol. 1998; 8: 280–5. 9477283 10.1007/s003300050380

[55] Toker ME , Eren E , Akbayrak H *et al* Combined approach to a peripheral congenital arteriovenous malformation: surgery and embolization. Heart Vessel. 2006; 21: 127–30. 10.1007/s00380-005-0842-8 16550315

[56] Doppman JL , Pevsner P . Embolization of arteriovenous malformations by direct percutaneous puncture. AJR Am. J. Roentgenol. 1983; 140: 773–8. 6601389 10.2214/ajr.140.4.773

[57] Aikawa H , Okino Y , Yamada Y *et al* [A review of embolization for arteriovenous malformations (fistulae)]. Oita Kenritsu Byoin Igaku Zasshi 1997; 26: 77–82. Japanese.

[58] Yamamoto T , Kanamura N , Tsukitani K *et al* Direct embolization for a life‐threatening mandibular arteriovenous malformation by means of hypothermic cardio‐pulmonary arrest. Kyoto Furitsu Ika Daigaku Zasshi 1999; 108: 981–94.

[59] Deng W , Huang D , Chen S *et al* Management of high‐flow arteriovenous malformation in the maxillofacial region. J. Craniofac. Surg. 2010; 21: 916–9. 20485081 10.1097/SCS.0b013e3181d880fd

[60] Erdmann MW , Jackson JE , Davies DM , Allison DJ . Multidisciplinary approach to the management of head and neck arteriovenous malformations. Ann. R. Coll. Surg. Engl. 1995; 77: 53–9. 7717646 PMC2502522

[61] Goldberg RA , Garcia GH , Duckwiler GR . Combined embolization and surgical treatment of arteriovenous malformation of the orbit. Am. J. Ophthalmol. 1993; 116: 17–25. 8328538 10.1016/s0002-9394(14)71738-6

[62] Persky MS , Yoo HJ , Berenstein A . Management of vascular malformations of the mandible and maxilla. Laryngoscope 2003; 113: 1885–92. 14603041 10.1097/00005537-200311000-00005

[63] Liu DG , Ma XC , Zhao FY , Zhang JG . A preliminary study of angiographic classification and its correlation to treatment of central arteriovenous malformation in the jaw. Oral Surg. Oral Med. Oral Pathol. Oral Radiol. Endod. 2005; 100: 473–80. 16182169 10.1016/j.tripleo.2005.01.006

[64] Rodesch G , Soupre V , Vazquez MP , Alvarez H , Lasjaunias P . Arteriovenous malformations of the dental arcades. The place of endovascular therapy: results in 12 cases are presented. J. Craniomaxillofac. Surg. 1998; 26: 306–13. 9819681 10.1016/s1010-5182(98)80059-0

[65] Chen W , Wang J , Li J , Xu L . Comprehensive treatment of arteriovenous malformations in the oral and maxillofacial region. J. Oral Maxillofac. Surg. 2005; 63: 1484–88. 16182916 10.1016/j.joms.2005.04.036

[66] Chen WL , Ye JT , Xu LF , Huang ZQ , Zhang DM . A multidisciplinary approach to treating maxillofacial arteriovenous malformations in children. Oral Surg. Oral Med. Oral Pathol. Oral Radiol. Endod. 2009; 108: 41–7. 19464212 10.1016/j.tripleo.2009.03.006

[67] Churojana A , Khumtong R , Songsaeng D , Chongkolwatana C , Suthipongchai S . Life‐threatening arteriovenous malformation of the maxillomandibular region and treatment outcomes. Interv. Neuroradiol. 2012; 18: 49–59. 22440601 10.1177/159101991201800107 PMC3312089

[68] Liu D , Ma XC . Clinical study of embolization of arteriovenous malformation in the oral and maxillofacial region. Chin. J. Dent. Res. 2000; 3: 63–70. 11314538

[69] Chandra RV , Leslie‐Mazwi TM , Orbach DB , Kaban LB , Rabinov JD . Transarterial embolization of mandibular arteriovenous malformations using ONYX. J. Oral. Maxillofac. Surg. 2014; 72: 1504–10. 24704035 10.1016/j.joms.2014.02.025

[70] Fifi J , Niimi Y , Berenstein A . Onyx embolization of an extensive mandibular arteriovenous malformation via a dual lumen balloon catheter: a technical case report. J. Neurointerv. Surg. 2013; 5: e5. 22248630 10.1136/neurintsurg-2011-010179

[71] Fan XD , Su LX , Zheng JW , Zheng LZ , Zhang ZY . Ethanol embolization of arteriovenous malformations of the mandible. AJNR Am. J. Neuroradiol. 2009; 30: 1178–83. 19270102 10.3174/ajnr.A1539 PMC7051317

[72] Park HS , Do YS , Park KB *et al* Ethanol embolotherapy of hand arteriovenous malformations. J. Vasc. Surg. 2011; 53: 725–31. 21145689 10.1016/j.jvs.2010.09.028

[73] Park UJ , Do YS , Park KB *et al* Treatment of arteriovenous malformations involving the hand. Ann. Vasc. Surg. 2012; 26: 643–8. 22266239 10.1016/j.avsg.2011.08.016

[74] Hibino N , Hamada Y , Goda Y *et al* [Arterial graft for reconstruction after complete removal of the arteriovenous malformation]. Nihon Maikuro Sajari Gakkai Kaishi 2005; 18: 78–82. Japanese.

[75] Widlus DM , Murray RR , White RI Jr *et al* Congenital arteriovenous malformations: tailored embolotherapy. Radiology 1988; 169: 511–6. 3175000 10.1148/radiology.169.2.3175000

[76] Hattori Y , Doi K , Kawakami F , Watanabe M . Extended wrap‐around flap for thumb reconstruction following radical excision of a congenital arteriovenous fistula. J. Hand Surg. Br. 1998; 23: 72–5. 9571486 10.1016/s0266-7681(98)80224-6

[77] Sugioka T , Sunagawa T , Suzuki O , Kijima Y , Ochi M . [Surgical treatment for arteriovenous malformation involving the hand and forearm]. Nihon Te No Geka Gakkai Zasshi 2008; 24: 940–3. Japanese.

[78] Furuya T , Nakazawa T . Congenital arteriovenous malformation of the index finger: a case report. Myakkangaku 2009; 49: 430–3. Japanese.

[79] Moore JR , Weiland AJ . Embolotherapy in the treatment of congenital arteriovenous malformations of the hand: a case report. J Hand Surg Am 1985; 10: 135–9. 3968395 10.1016/s0363-5023(85)80265-3

[80] Arneja JS , Gosain AK . Vascular malformations. Plast. Reconstr. Surg. 2008; 121: 195e–206e. 10.1097/01.prs.0000304607.29622.3c 18349599

[81] Hein KD , Mulliken JB , Kozakewich HP , Upton J , Burrows PE . Venous malformations of skeletal muscle. Plast. Reconstr. Surg. 2002; 110: 1625–35. 12447041 10.1097/01.PRS.0000033021.60657.74

[82] Marler JJ , Mulliken JB . Current management of hemangiomas and vascular malformations. Clin. Plast. Surg. 2005; 32: 99–116, ix. 15636768 10.1016/j.cps.2004.10.001

[83] Nguyen JT , Koerper MA , Hess CP *et al* Aspirin therapy in venous malformation: a retrospective cohort study of benefits, side effects, and patient experiences. Pediatr. Dermatol. 2014; 31: 556–60. 25040175 10.1111/pde.12373

[84] Shireman PK , McCarthy WJ , Yao JS , Vogelzang RL . Treatment of venous malformations by direct injection with ethanol. J. Vasc. Surg. 1997; 26: 838–44. 9372823 10.1016/s0741-5214(97)70098-3

[85] Rimon U , Garniek A , Galili Y , Golan G , Bensaid P , Morag B . Ethanol sclerotherapy of peripheral venous malformations. Eur. J. Radiol. 2004; 52: 283–7. 15544907 10.1016/j.ejrad.2003.09.010

[86] Marrocco‐Trischitta MM , Nicodemi EM , Nater C , Stillo F . Management of congenital venous malformations of the vulva. Obstet. Gynecol. 2001; 98: 789–93. 11704170

[87] Suh JS , Shin KH , Na JB , Won JY , Hahn SB . Venous malformations: sclerotherapy with a mixture of ethanol and lipiodol. Cardiovasc. Intervent. Radiol. 1997; 20: 268–73. 9211773 10.1007/s002709900150

[88] Dompmartin A , Blaizot X , Théron J *et al* Radio‐opaque ethylcellulose‐ethanol is a safe and efficient sclerosing agent for venous malformations. Eur. Radiol. 2011; 21: 2647–56. 21822948 10.1007/s00330-011-2213-4

[89] Schumacher M , Dupuy P , Bartoli JM *et al* Treatment of venous malformations: first experience with a new sclerosing agent–a multicenter study. Eur. J. Radiol. 2011; 80: e366–72. 21458186 10.1016/j.ejrad.2010.12.074

[90] Mimura H , Kanazawa S , Yasui K *et al* Percutaneous sclerotherapy for venous malformations using polidocanol under fluoroscopy. Acta Med. Okayama 2003; 57: 227–34. 14679400 10.18926/AMO/51909

[91] Mimura H , Fujiwara H , Hiraki T *et al* Polidocanol sclerotherapy for painful venous malformations: evaluation of safety and efficacy in pain relief. Eur. Radiol. 2009; 19: 2474–80. 19440712 10.1007/s00330-009-1442-2

[92] Cabrera J , Cabrera J Jr , Garcia‐Olmedo MA , Redondo P . Treatment of venous malformations with sclerosant in microfoam form. Arch. Dermatol. 2003; 139: 1409–16. 14623700 10.1001/archderm.139.11.1409

[93] Ozaki M , Kurita M , Kaji N *et al* Efficacy and evaluation of safety of sclerosants for intramuscular venous malformations: clinical and experimental studies. Scand. J. Plast. Reconstr. Surg. Hand Surg. 2010; 44: 75–87. 20465508 10.3109/02844310903569725

[94] Krokidis M , Venetucci P , Hatzidakis A , Iaccarino V . Sodium tetradecyl sulphate direct intralesional sclerotherapy of venous malformations of the vulva and vagina: report of five cases. Cardiovasc. Intervent. Radiol. 2011; 34 (Suppl 2): S228–31. 20559638 10.1007/s00270-010-9916-9

[95] Enjolras O , Ciabrini D , Mazoyer E , Laurian C , Herbreteau D . Extensive pure venous malformations in the upper or lower limb: a review of 27 cases. J. Am. Acad. Dermatol. 1997; 36: 219–25. 9039172 10.1016/s0190-9622(97)70284-6

[96] Steiner F , FitzJohn T , Tan ST . Surgical treatment for venous malformation. J. Plast. Reconstr. Aesthet. Surg. 2013; 66: 1741–49. 24012651 10.1016/j.bjps.2013.07.033

[97] Noel AA , Gloviczki P , Cherry KJ Jr , Rooke TW , Stanson AW , Driscoll DJ . Surgical treatment of venous malformations in Klippel‐Trenaunay syndrome. J. Vasc. Surg. 2000; 32: 840–7. 11054214 10.1067/mva.2000.110343

[98] Sidhu MK , Perkins JA , Shaw DW , Bittles MA , Andrews RT . Ultrasound‐guided endovenous diode laser in the treatment of congenital venous malformations: preliminary experience. J. Vasc. Interv. Radiol. 2005; 16: 879–84. 15947054 10.1097/01.RVI.0000163005.50283.62

[99] Lu X , Ye K , Shi H *et al* Percutaneous endovenous treatment of congenital extratruncular venous malformations with an ultrasound‐guided and 810‐nm diode laser. J. Vasc. Surg. 2011; 54: 139–45. 21277147 10.1016/j.jvs.2010.11.105

[100] Liu G , Liu X , Li W *et al* Ultrasound‐guided intralesional diode laser treatment of congenital extratruncular venous malformations: mid‐term results. Eur. J. Vasc. Endovasc. Surg. 2014; 47: 558–64. 24656873 10.1016/j.ejvs.2014.02.014

[101] Mazoyer E , Enjolras O , Laurian C , Houdart E , Drouet L . Coagulation abnormalities associated with extensive venous malformations of the limbs: differentiation from Kasabach‐Merritt syndrome. Clin. Lab. Haematol. 2002; 24: 243–51. 12181029 10.1046/j.1365-2257.2002.00447.x

[102] Cornelis F , Neuville A , Labreze C *et al* Percutaneous cryotherapy of vascular malformation: initial experience. Cardiovasc. Intervent. Radiol. 2013; 36: 853–6. 22722720 10.1007/s00270-012-0434-9

[103] Cornelis F , Havez M , Labreze C *et al* Percutaneous cryoablation of symptomatic localized venous malformations: preliminary short‐term results. J. Vasc. Interv. Radiol. 2013; 24: 823–7. 23707090 10.1016/j.jvir.2013.03.005

[104] Betz CS , Jager HR , Brookes JA , Richards R , Leunig A , Hopper C . Interstitial photodynamic therapy for a symptom‐targeted treatment of complex vascular malformations in the head and neck region. Lasers Surg. Med. 2007; 39: 571–82. 17868106 10.1002/lsm.20535

[105] Bashkatov AN , Genina EA , Kochubey VI , Tuchin VV . Optical properties of human skin, subcutaneous and mucous tissues in the wavelength range from 400 to 2000 nm. J. Phys. D Appl. Phys. 2005; 38: 2543–55.

[106] Sarig O , Kimel S , Orenstein A . Laser treatment of venous malformations. Ann. Plast. Surg. 2006; 57: 20–24. 16799303 10.1097/01.sap.0000210632.40697.9b

[107] Vesnaver A , Dovsak DA . Treatment of vascular lesions in the head and neck using Nd:YAG laser. J. Craniomaxillofac. Surg. 2006; 34: 17–24. 10.1016/j.jcms.2005.07.009 16352435

[108] Asai T , Suzuki H , Enomoto Y *et al* Clinical evaluation of 74 vascular malformations of the oral region treated by photocoagulation with an Nd:YAG laser. Nihon Reza Shigakkaishi 2013; 24: 3–9. Japanese.

[109] Ng EK , Cheung FK , Chiu PW . Blue rubber bleb nevus syndrome: treatment of multiple gastrointestinal hemangiomas with argon plasma coagulator. Dig. Endosc. 2009; 21: 40–2. 19691801 10.1111/j.1443-1661.2008.00817.x

[110] Cholewa D , Waldschmidt J . Laser treatment of hemangiomas of the larynx and trachea. Lasers Surg. Med. 1998; 23: 221–32. 9829433 10.1002/(sici)1096-9101(1998)23:4<221::aid-lsm5>3.0.co;2-a

[111] Yakes WF , Haas DK , Parker SH *et al* Symptomatic vascular malformations: ethanol embolotherapy. Radiology 1989; 170: 1059–66. 2916057 10.1148/radiology.170.3.2916057

[112] Lee IH , Kim KH , Jeon P *et al* Ethanol sclerotherapy for the management of craniofacial venous malformations: the interim results. Korean J. Radiol. 2009; 10: 269–76. 19412515 10.3348/kjr.2009.10.3.269 PMC2672182

[113] Hoque S , Das BK . Treatment of venous malformations with ethanolamine oleate: a descriptive study of 83 cases. Pediatr. Surg. Int. 2011; 27: 527–31. 21290138 10.1007/s00383-010-2824-x

[114] Stuart S , Barnacle AM , Smith G , Pitt M , Roebuck DJ . Neuropathy after sodium tetradecyl sulfate sclerotherapy of venous malformations in children. Radiology 2015; 274: 897–905. 25271855 10.1148/radiol.14132271

[115] Zhao JH , Zhang WF , Zhao YF . Sclerotherapy of oral and facial venous malformations with use of pingyangmycin and/or sodium morrhuate. Int. J. Oral. Maxillofac. Surg. 2004; 33: 463–6. 15183410 10.1016/j.ijom.2003.10.003

[116] Bai N , Chen YZ , Fu YJ , Wu P , Zhang WN . A clinical study of pingyangmycin sclerotherapy for venous malformation: an evaluation of 281 consecutive patients. J. Clin. Pharm. Ther. 2014; 39: 521–6. 24924412 10.1111/jcpt.12183

[117] Goyal M , Causer PA , Armstrong D . Venous vascular malformations in pediatric patients: comparison of results of alcohol sclerotherapy with proposed MR imaging classification. Radiology 2002; 223: 639–644. 12034929 10.1148/radiol.2233010025

[118] Yun WS , Kim YW , Lee KB *et al* Predictors of response to percutaneous ethanol sclerotherapy (PES) in patients with venous malformations: analysis of patient self‐assessment and imaging. J. Vasc. Surg. 2009; 50: 581–9, 589 e581. 19540703 10.1016/j.jvs.2009.03.058

[119] Rautio R , Saarinen J , Laranne J , Salenius JP , Keski‐Nisula L . Endovascular treatment of venous malformations in extremities: results of sclerotherapy and the quality of life after treatment. Acta Radiol. 2004; 45: 397–403. 15323391 10.1080/02841850410004913

[120] Yamaki T , Nozaki M , Sakurai H , Takeuchi M , Soejima K , Kono T . Prospective randomized efficacy of ultrasound‐guided foam sclerotherapy compared with ultrasound‐guided liquid sclerotherapy in the treatment of symptomatic venous malformations. J. Vasc. Surg. 2008; 47: 578–84. 18295109 10.1016/j.jvs.2007.11.026

[121] Nagao M , Sasaki S , Furukawa H , Saito N , Yamamoto Y . [A clinical study of sclerotherapy for venous malformation of an upper extremity]. Nihon Keisei Geka Gakkai Kaishi 2012; 32: 463–8. Japanese.

[122] Nomura T , Sakurai A , Nagata I , Terashi H , Tahara S . Our strategy for the treatment of venous malformations. Jomyakugaku 2008; 19: 161–8. Japanese.

[123] Qiu Y , Chen H , Lin X , Hu X , Jin Y , Ma G . Outcomes and complications of sclerotherapy for venous malformations. Vasc. Endovascular. Surg. 2013; 47: 454–61. 23759722 10.1177/1538574413492390

[124] Wong GA , Armstrong DC , Robertson JM . Cardiovascular collapse during ethanol sclerotherapy in a pediatric patient. Paediatr. Anaesth. 2006; 16: 343–6. 16490104 10.1111/j.1460-9592.2005.01710.x

[125] Tachibana K , Kobayashi S , Kojima T , Kaseno S , Kemmotsu O . Pulmonary emboli in sclerotherapy for peripheral vascular malformations under general anesthesia; a report of two cases. Masui 2004; 53: 645–9. Japanese. 15242036

[126] Marrocco‐Trischitta MM , Guerrini P , Abeni D , Stillo F . Reversible cardiac arrest after polidocanol sclerotherapy of peripheral venous malformation. Dermatol. Surg. 2002; 28: 153–5. 11860427 10.1046/j.1524-4725.2002.00344.x

[127] Shimo T , Hidaka K , Yanagawa S , Kadota W , Kawakami S , Tsuchida H . Two episodes of cardiac arrest in a boy receiving sclerotherapy with polydocanol–a case report. Masui 2005; 54: 57–9. Japanese. 15717471

[128] Schild SE , Buskirk SJ , Frick LM , Cupps RE . Radiotherapy for large symptomatic hemangiomas. Int. J. Radiat. Oncol. Biol. Phys. 1991; 21: 729–35. 1869466 10.1016/0360-3016(91)90693-x

[129] Mitsuhashi N , Furuta M , Sakurai H *et al* Outcome of radiation therapy for patients with Kasabach‐Merritt syndrome. Int. J. Radiat. Oncol. Biol. Phys. 1997; 39: 467–73. 9308952 10.1016/s0360-3016(97)00140-5

[130] Ogino I , Torikai K , Kobayasi S , Aida N , Hata M , Kigasawa H . Radiation therapy for life‐ or function‐threatening infant hemangioma. Radiology 2001; 218: 834–9. 11230664 10.1148/radiology.218.3.r01mr04834

[131] Miller JG , Orton CI . Long term follow‐up of a case of Kasabach‐Merritt syndrome successfully treated with radiotherapy and corticosteroids. Br. J. Plast. Surg. 1992; 45: 559–61. 1446205 10.1016/0007-1226(92)90157-s

[132] Frevel T , Rabe H , Uckert F , Harms E . Giant cavernous haemangioma with Kasabach‐Merritt syndrome: a case report and review. Eur. J. Pediatr. 2002; 161: 243–6. 12012216 10.1007/s00431-002-0953-5

[133] Stringel G , Mercer S . Giant hemangioma in the newborn and infant. Complications and management. Clin. Pediatr. (Phila) 1984; 23: 498–502. 6467783 10.1177/000992288402300910

[134] Enjolras O , Wassef M , Mazoyer E *et al* Infants with Kasabach‐Merritt syndrome do not have "true" hemangiomas. J. Pediatr. 1997; 130: 631–40. 9108863 10.1016/s0022-3476(97)70249-x

[135] Lundell M , Mattsson A , Hakulinen T , Holm LE . Breast cancer after radiotherapy for skin hemangioma in infancy. Radiat. Res. 1996; 145: 225–30. 8606933

[136] Haddy N , Andriamboavonjy T , Paoletti C *et al* Thyroid adenomas and carcinomas following radiotherapy for a hemangioma during infancy. Radiother. Oncol. 2009; 93: 377–82. 19515442 10.1016/j.radonc.2009.05.011

[137] Caldwell JB , Ryan MT , Benson PM , James WD . Cutaneous angiosarcoma arising in the radiation site of a congenital hemangioma. J. Am. Acad. Dermatol. 1995; 33: 865–70. 7593798 10.1016/0190-9622(95)90424-7

[138] Oguri A , Oda M , Yokoo K . [The relation between age and the effectiveness of laser treatment for capillary malformation]. Nihon Keisei Geka Gakkai Kaishi 2009; 29: 407–11. Japanese.

[139] Reynolds N , Exley J , Hills S , Falder S , Duff C , Kenealy J . The role of the Lumina intense pulsed light system in the treatment of port wine stains–a case controlled study. Br. J. Plast. Surg. 2005; 58: 968–80. 16043156 10.1016/j.bjps.2005.04.006

[140] Katugampola GA , Lanigan SW . Five years' experience of treating port wine stains with the flashlamp‐pumped pulsed dye laser. Br. J. Dermatol. 1997; 137: 750–4. 9415235

[141] Minato S . Comparison between results of treatment with dye laser and argon laser in cases of hemangioma simplex. Iwate Igaku Zasshi 1997; 49: 299–302.

[142] Ryuzaki K , Tamura A , Amano H , Takeuchi Y , Ishikawa O , Miyachi Y . [An overview of dye laser treatment for superficial hemangiomas at the Department of Dermatology, Gunma University]. Hifuka Kiyo 1996; 91: 41–6. Japanese.

[143] Matsumoto T . [Comparative study in laser treatment of portwine stains with dye and argon lasers. Part 2: statistical study of clinical effects]. Nihon Keisei Geka Gakkai Kaishi 1996; 16: 246–59. Japanese.

[144] Fitzpatrick RE , Lowe NJ , Goldman MP , Borden H , Behr KL , Ruiz‐Esparza J . Flashlamp‐pumped pulsed dye laser treatment of port‐wine stains. J. Dermatol. Surg. Oncol. 1994; 20: 743–8. 7962935 10.1111/j.1524-4725.1994.tb03197.x

[145] Morikawa K , Yamauchi K , Saheki M , Tezuka T . Treatment of portwine stain using pulsed dye laser: comparison of effectiveness with location of lesion, age and wavelength. Hifu 1994; 36: 514–21. Japanese.

[146] Matsushita Y , Suzuki S , Koyama H , Akamatsu J , Kawata Y , Kawai K . Treatment of portwine stains with dye lasers. Hifuka Kiyo 1994; 89: 205–10. Japanese.

[147] Namba Y , Mae O , Nagase Y , Ao M , Hamaya K , Nose S . [Dye laser treatment of cutaneous simple hemangioma]. Okayama Saiseikai Sogo Byoin Zasshi 1991; 22: 1–10. Japanese.

[148] Bandoh Y , Yanai A , Tsuzuki K . Dye laser treatment of port‐wine stains. Aesthet. Plast. Surg. 1990; 14: 287–91. 10.1007/BF01578363 2239519

[149] Bandoh Y . [Pulsed dye laser treatment for port‐wine stains]. Rinsho Hifuka 1989; 43: 1337–40. Japanese.

[150] Renfro L , Geronemus RG . Anatomical differences of port‐wine stains in response to treatment with the pulsed dye laser. Arch. Dermatol. 1993; 129: 182–8. 8434975

[151] Lanigan SW . Port wine stains on the lower limb: response to pulsed dye laser therapy. Clin. Exp. Dermatol. 1996; 21: 88–92. 8759191

[152] Sommer S , Seukeran DC , Sheehan‐Dare RA . Efficacy of pulsed dye laser treatment of port wine stain malformations of the lower limb. Br. J. Dermatol. 2003; 149: 770–5. 14616368 10.1046/j.1365-2133.2003.05467.x

[153] Fitzpatrick TB . The validity and practicality of sun‐reactive skin types I through VI. Arch. Dermatol. 1988; 124: 869–71. 3377516 10.1001/archderm.124.6.869

[154] Wareham WJ , Cole RP , Royston SL , Wright PA . Adverse effects reported in pulsed dye laser treatment for port wine stains. Lasers Med. Sci. 2009; 24: 241–6. 18418641 10.1007/s10103-008-0560-4

[155] Orten SS , Waner M , Flock S , Roberson PK , Kincannon J . Port‐wine stains. An assessment of 5 years of treatment. Arch. Otolaryngol. Head Neck Surg. 1996; 122: 1174–9. 8906051 10.1001/archotol.1996.01890230022005

[156] Michel S , Landthaler M , Hohenleutner U . Recurrence of port‐wine stains after treatment with the flashlamp‐pumped pulsed dye laser. Br. J. Dermatol. 2000; 143: 1230–4. 11122026 10.1046/j.1365-2133.2000.03893.x

[157] Soueid A , Waters R . Re‐emergence of port wine stains following treatment with flashlamp‐pumped dye laser 585 nm. Ann. Plast. Surg. 2006; 57: 260–3. 16929190 10.1097/01.sap.0000221620.83178.fc

[158] Huikeshoven M , Koster PH , de Borgie CA , Beek JF , van Gemert MJ , van der Horst CM . Redarkening of port‐wine stains 10 years after pulsed‐dye‐laser treatment. N. Engl. J. Med. 2007; 356: 1235–40. 17377161 10.1056/NEJMoa064329

[159] Phung TL , Oble DA , Jia W , Benjamin LE , Mihm MC Jr , Nelson JS . Can the wound healing response of human skin be modulated after laser treatment and the effects of exposure extended? Implications on the combined use of the pulsed dye laser and a topical angiogenesis inhibitor for treatment of port wine stain birthmarks. Lasers Surg. Med. 2008; 40: 1–5. 18220264 10.1002/lsm.20599 PMC3496566

[160] Babilas P , Shafirstein G , Bäumler W *et al* Selective photothermolysis of blood vessels following flashlamp‐pumped pulsed dye laser irradiation: in vivo results and mathematical modelling are in agreement. J. Invest. Dermatol. 2005; 125: 343–52. 16098046 10.1111/j.0022-202X.2005.23773.x

[161] Laquer VT , Hevezi PA , Albrecht H , Chen TS , Zlotnik A , Kelly KM . Microarray analysis of port wine stains before and after pulsed dye laser treatment. Lasers Surg. Med. 2013; 45: 67–75. 23440713 10.1002/lsm.22087 PMC3930171

[162] Chapas AM , Eickhorst K , Geronemus RG . Efficacy of early treatment of facial port wine stains in newborns: a review of 49 cases. Lasers Surg. Med. 2007; 39: 563–8. 17868100 10.1002/lsm.20529

[163] Jia W , Sun V , Tran N *et al* Long‐term blood vessel removal with combined laser and topical rapamycin antiangiogenic therapy: implications for effective port wine stain treatment. Lasers Surg. Med. 2010; 42: 105–12. 20166161 10.1002/lsm.20890 PMC2871771

[164] Chang CJ , Hsiao YC , Mihm MC Jr , Nelson JS . Pilot study examining the combined use of pulsed dye laser and topical Imiquimod versus laser alone for treatment of port wine stain birthmarks. Lasers Surg. Med. 2008; 40: 605–10. 18951427 10.1002/lsm.20716 PMC2717850

[165] Tremaine AM , Armstrong J , Huang YC *et al* Enhanced port‐wine stain lightening achieved with combined treatment of selective photothermolysis and imiquimod. J. Am. Acad. Dermatol. 2012; 66: 634–41. 22244840 10.1016/j.jaad.2011.11.958 PMC3327725

[166] Nguyen CM , Yohn JJ , Huff C , Weston WL , Morelli JG . Facial port wine stains in childhood: prediction of the rate of improvement as a function of the age of the patient, size and location of the port wine stain and the number of treatments with the pulsed dye (585 nm) laser. Br. J. Dermatol. 1998; 138: 821–5. 9666828 10.1046/j.1365-2133.1998.02219.x

[167] van der Horst CM , Koster PH , de Borgie CA , Bossuyt PM , van Gemert MJ . Effect of the timing of treatment of port‐wine stains with the flash‐lamp‐pumped pulsed‐dye laser. N. Engl. J. Med. 1998; 338: 1028–1033. 9535667 10.1056/NEJM199804093381504

[168] Léauté‐Labrèze C , de la Roque ED , Hubiche T , Boralevi F , Thambo J‐B , Taïeb A . Propranolol for severe hemangiomas of infancy. N. Engl. J. Med. 2008; 358: 2649–51. 18550886 10.1056/NEJMc0708819

[169] Broeks IJ , Hermans DJ , Dassel AC , van der Vleuten CJ , van Beynum IM . Propranolol treatment in life‐threatening airway hemangiomas: a case series and review of literature. Int. J. Pediatr. Otorhinolaryngol. 2013; 77: 1791–1800. 24074695 10.1016/j.ijporl.2013.08.011

[170] Sharma VK , Fraulin FO , Dumestre DO , Walker L , Harrop AR . Beta‐blockers for the treatment of problematic hemangiomas. Can. J. Plast. Surg. 2013; 21: 23–8. 24431932 10.1177/229255031302100103 PMC3891114

[171] Zvulunov A , McCuaig C , Frieden IJ *et al* Oral propranolol therapy for infantile hemangiomas beyond the proliferation phase: a multicenter retrospective study. Pediatr. Dermatol. 2011; 28: 94–8. 21362031 10.1111/j.1525-1470.2010.01379.x

[172] Hermans DJ , van Beynum IM , Schultze Kool LJ , van de Kerkhof PC , Wijnen MH , van der Vleuten CJ . Propranolol, a very promising treatment for ulceration in infantile hemangiomas: a study of 20 cases with matched historical controls. J. Am. Acad. Dermatol. 2011; 64: 833–838. 21353329 10.1016/j.jaad.2011.01.025

[173] Saint‐Jean M , Léauté‐Labrèze C , Mazereeuw‐Hautier J *et al* Propranolol for treatment of ulcerated infantile hemangiomas. J. Am. Acad. Dermatol. 2011; 64: 827–32. 21353332 10.1016/j.jaad.2010.12.040

[174] Caussé S , Aubert H , Saint‐Jean M *et al* Propranolol‐resistant infantile haemangiomas. Br. J. Dermatol. 2013; 169: 125–9. 23659587 10.1111/bjd.12417

[175] Vassallo P , Forte R , Di Mezza A , Magli A . Treatment of infantile capillary hemangioma of the eyelid with systemic propranolol. Am. J. Ophthalmol. 2013; 155: 165–70 e162. 22967870 10.1016/j.ajo.2012.06.021

[176] Lynch M , Lenane P , O'Donnell BF . Propranolol for the treatment of infantile haemangiomas: our experience with 44 patients. Clin. Exp. Dermatol. 2014; 39: 142–5. 24289272 10.1111/ced.12210

[177] Price CJ , Lattouf C , Baum B *et al* Propranolol vs corticosteroids for infantile hemangiomas: a multicenter retrospective analysis. Arch. Dermatol. 2011; 147: 1371–6. 21844428 10.1001/archdermatol.2011.203

[178] Hermans DJ , Bauland CG , Zweegers J , van Beynum IM , van der Vleuten CJ . Propranolol in a case series of 174 patients with complicated infantile haemangioma: indications, safety and future directions. Br. J. Dermatol. 2013; 168: 837–43. 23278381 10.1111/bjd.12189

[179] de Graaf M , Breur J , Raphael MF , Vos M , Breugem CC , Pasmans S . Adverse effects of propranolol when used in the treatment of hemangiomas: a case series of 28 infants. J. Am. Acad. Dermatol. 2011; 65: 320–7. 21601311 10.1016/j.jaad.2010.06.048

[180] Hogeling M , Adams S , Wargon O . A randomized controlled trial of propranolol for infantile hemangiomas. Pediatrics 2011; 128: e259–66. 21788220 10.1542/peds.2010-0029

[181] Zaher H , Rasheed H , Esmat S *et al* Propranolol and infantile hemangiomas: different routes of administration, a randomized clinical trial. Eur. J. Dermatol. 2013; 23: 646–52. 24135427 10.1684/ejd.2013.2146

[182] Malik MA , Menon P , Rao KL , Samujh R . Effect of propranolol vs prednisolone vs propranolol with prednisolone in the management of infantile hemangioma: a randomized controlled study. J. Pediatr. Surg. 2013; 48: 2453–9. 24314186 10.1016/j.jpedsurg.2013.08.020

[183] Bauman NM , McCarter RJ , Guzzetta PC *et al* Propranolol vs prednisolone for symptomatic proliferating infantile hemangiomas: a randomized clinical trial. JAMA Otolaryngol. Head Neck Surg. 2014; 140: 323–30. 24526257 10.1001/jamaoto.2013.6723

[184] Léauté‐Labrèze C , Dumas de la Roque E , Nacka F *et al* Double‐blind randomized pilot trial evaluating the efficacy of oral propranolol on infantile haemangiomas in infants < 4 months of age. Br. J. Dermatol. 2013; 169: 181–3. 23301692 10.1111/bjd.12217

[185] Abarzúa‐Araya A , Navarrete‐Dechent CP , Heusser F , Retamal J , Zegpi‐Trueba MS . Atenolol versus propranolol for the treatment of infantile hemangiomas: a randomized controlled study. J. Am. Acad. Dermatol. 2014; 70: 1045–9. 24656727 10.1016/j.jaad.2014.01.905

[186] Ehsani AH , Noormohammadpoor P , Abdolreza M , Balighi K , Arianian Z , Daklan S . Combination therapy of infantile hemangioma with pulsed dye laser with topical propranolol: a randomized clinical trial. Arch. Iran Med. 2014; 17: 657–60. 25305763

[187] Léauté‐Labrèze C , Hoeger P , Mazereeuw‐Hautier J *et al* A randomized, controlled trial of oral propranolol in infantile hemangioma. N. Engl. J. Med. 2015; 372: 735–46. 25693013 10.1056/NEJMoa1404710

[188] Menezes MD , McCarter R , Greene EA , Bauman NM . Status of propranolol for treatment of infantile hemangioma and description of a randomized clinical trial. Ann. Otol. Rhinol. Laryngol. 2011; 120: 686–95. 22097156 10.1177/000348941112001010

[189] Marqueling AL , Oza V , Frieden IJ , Puttgen KB . Propranolol and infantile hemangiomas four years later: a systematic review. Pediatr. Dermatol. 2013; 30: 182–91. 23405852 10.1111/pde.12089

[190] Xu SQ , Jia RB , Zhang W , Zhu H , Ge SF , Fan XQ . Beta‐blockers versus corticosteroids in the treatment of infantile hemangioma: an evidence‐based systematic review. World J. Pediatr. 2013; 9: 221–9. 23929254 10.1007/s12519-013-0427-z

[191] Izadpanah A , Izadpanah A , Kanevsky J , Belzile E , Schwarz K . Propranolol versus corticosteroids in the treatment of infantile hemangioma: a systematic review and meta‐analysis. Plast. Reconstr. Surg. 2013; 131: 601–13. 23142941 10.1097/PRS.0b013e31827c6fab

[192] Xu S , Jia R , Ge S , Lin M , Fan X . Treatment of periorbital infantile haemangiomas: a systematic literature review on propranolol or steroids. J. Paediatr. Child Health 2014; 50: 271–9. 24754793 10.1111/jpc.12464

[193] Peridis S , Pilgrim G , Athanasopoulos I , Parpounas K . A meta‐analysis on the effectiveness of propranolol for the treatment of infantile airway haemangiomas. Int. J. Pediatr. Otorhinolaryngol. 2011; 75: 455–60. 21333364 10.1016/j.ijporl.2011.01.028

[194] Vlastarakos PV , Papacharalampous GX , Chrysostomou M *et al* Propranolol is an effective treatment for airway haemangiomas: a critical analysis and meta‐analysis of published interventional studies. Acta Otorhinolaryngol. Ital. 2012; 32: 213–21. 23093810 PMC3468939

[195] Storch CH , Hoeger PH . Propranolol for infantile haemangiomas: insights into the molecular mechanisms of action. Br. J. Dermatol. 2010; 163: 269–74. 20456345 10.1111/j.1365-2133.2010.09848.x

[196] Drolet BA , Frommelt PC , Chamlin SL *et al* Initiation and use of propranolol for infantile hemangioma: report of a consensus conference. Pediatrics 2013; 131: 128–40. 23266923 10.1542/peds.2012-1691 PMC3529954

[197] Kum JJ , Khan ZA . Mechanisms of propranolol action in infantile hemangioma. Dermatoendocrinol 2014; 6: e979699. 26413184 10.4161/19381980.2014.979699 PMC4580045

[198] Horev A , Haim A , Zvulunov A . Propranolol induced hypoglycemia. Pediatr. Endocrinol. Rev. 2015; 12: 308–10. 25962208

[199] Blei F , McElhinney DB , Guarini A , Presti S . Cardiac screening in infants with infantile hemangiomas before propranolol treatment. Pediatr. Dermatol. 2014; 31: 465–70. 24889812 10.1111/pde.12344

[200] Raphael MF , Breugem CC , Vlasveld FA *et al* Is cardiovascular evaluation necessary prior to and during beta‐blocker therapy for infantile hemangiomas?: A cohort study. J. Am. Acad. Dermatol. 2015; 72: 465–72. 25592625 10.1016/j.jaad.2014.12.019

[201] Chamlin SL , Haggstrom AN , Drolet BA *et al* Multicenter prospective study of ulcerated hemangiomas. J. Pediatr. 2007; 151: 684–9, 689 e681. 18035154 10.1016/j.jpeds.2007.04.055

[202] Vercellino N , Romanini MV , Pelegrini M , Rimini A , Occella C , Dalmonte P . The use of propranolol for complicated infantile hemangiomas. Int. J. Dermatol. 2013; 52: 1140–6. 23829783 10.1111/j.1365-4632.2012.05795.x

[203] Sadykov RR , Podmelle F , Sadykov RA , Kasimova KR , Metellmann HR . Use of propranolol for the treatment infantile hemangiomas in the maxillofacial region. Int. J. Oral Maxillofac. Surg. 2013; 42: 863–7. 23618833 10.1016/j.ijom.2013.03.015

[204] Kim HJ , Colombo M , Frieden IJ . Ulcerated hemangiomas: clinical characteristics and response to therapy. J. Am. Acad. Dermatol. 2001; 44: 962–72. 11369908 10.1067/mjd.2001.112382

[205] Wananukul S , Chatproedprai S . Ulcerated hemangiomas: clinical features and management. J. Med. Assoc. Thai. 2002; 85: 1220–5. 12546320

[206] Pandey A , Gangopadhyay AN , Sharma SP , Kumar V , Gopal SC , Gupta DK . Conservative management of ulcerated haemangioma–twenty years experience. Int. Wound J. 2009; 6: 59–62. 19291117 10.1111/j.1742-481X.2008.00562.x PMC7951731

[207] Oranje AP , de Waard‐van der Spek FB , Devillers ACA , de Laat P , Madern GC . Treatment and pain relief of ulcerative hemangiomas with a polyurethane film. Dermatology 2000; 200: 31–4. 10681610 10.1159/000018311

[208] Bauland CG , Smit JM , Ketelaars R , Rieu PN , Spauwen PH . Management of haemangiomas of infancy: a retrospective analysis and a treatment protocol. Scand. J. Plast. Reconstr. Surg. Hand Surg. 2008; 42: 86–91. 18335352 10.1080/02844310801924167

[209] Morelli JG , Tan OT , Yohn JJ , Weston WL . Treatment of ulcerated hemangiomas infancy. Arch. Pediatr. Adolesc. Med. 1994; 148: 1104–5. 7921107 10.1001/archpedi.1994.02170100102023

[210] Lacour M , Syed S , Linward J , Harper JI . Role of the pulsed dye laser in the management of ulcerated capillary haemangiomas. Arch. Dis. Child. 1996; 74: 161–3. 8660082 10.1136/adc.74.2.161 PMC1511492

[211] David LR , Malek MM , Argenta LC . Efficacy of pulse dye laser therapy for the treatment of ulcerated haemangiomas: a review of 78 patients. Br. J. Plast. Surg. 2003; 56: 317–27. 12873458 10.1016/s0007-1226(03)00152-8

[212] Michel JL . Treatment of hemangiomas with 595 nm pulsed dye laser dermobeam. Eur. J. Dermatol. 2003; 13: 136–41. 12695128

[213] Di Maio L , Baldi A , Dimaio V , Barzi A . Use of flashlamp‐pumped pulsed dye laser in the treatment of superficial vascular malformations and ulcerated hemangiomas. In Vivo 2011; 25: 117–23. 21282744

[214] Sugarman JL , Mauro TM , Frieden IJ . Treatment of an ulcerated hemangioma with recombinant platelet‐derived growth factor. Arch. Dermatol. 2002; 138: 314–6. 11902979 10.1001/archderm.138.3.314

[215] Metz BJ , Rubenstein MC , Levy ML , Metry DW . Response of ulcerated perineal hemangiomas of infancy to becaplermin gel, a recombinant human platelet‐derived growth factor. Arch. Dermatol. 2004; 140: 867–70. 15262700 10.1001/archderm.140.7.867

[216] Jalil S , Akhtar J , Ahmed S . Corticosteroids therapy in the management of infantile cutaneous hemangiomas. J. Coll. Physicians Surg. Pak. 2006; 16: 662–5. 17007757 10.2006/JCPSP.662665

[217] Gangopadhyay AN , Sinha CK , Gopal SC , Gupta DK , Sahoo SP , Ahmad M . Role of steroid in childhood haemangioma: a 10 years review. Int. Surg. 1997; 82: 49–51. 9189802

[218] Pandey A , Gangopadhyay AN , Gopal SC *et al* Twenty years' experience of steroids in infantile hemangioma–a developing country's perspective. J. Pediatr. Surg. 2009; 44: 688–94. 19361627 10.1016/j.jpedsurg.2008.10.038

[219] Tan BH , Leadbitter PH , Aburn NH , Tan ST . Steroid therapy for problematic proliferating haemangioma. N. Z. Med. J. 2011; 124: 57–65. 21475361

[220] Ruttum MS , Abrams GW , Harris GJ , Ellis MK . Bilateral retinal embolization associated with intralesional corticosteroid injection for capillary hemangioma of infancy. J. Pediatr. Ophthalmol. Strabismus 1993; 30: 4–7. 8455125 10.3928/0191-3913-19930101-03

[221] Egbert JE , Schwartz GS , Walsh AW . Diagnosis and treatment of an ophthalmic artery occlusion during an intralesional injection of corticosteroid into an eyelid capillary hemangioma. Am. J. Ophthalmol. 1996; 121: 638–42. 8644806 10.1016/s0002-9394(14)70629-4

[222] Craiglow BG , Antaya RJ . Management of infantile hemangiomas: current and potential pharmacotherapeutic approaches. Paediatr. Drugs 2013; 15: 133–8. 23456550 10.1007/s40272-013-0008-6

[223] Ni N , Langer P , Wagner R , Guo S . Topical timolol for periocular hemangioma: report of further study. Arch. Ophthalmol. 2011; 129: 377–9. 21403002 10.1001/archophthalmol.2011.24

[224] Elsas FJ , Lewis AR . Topical treatment of periocular capillary hemangioma. J. Pediatr. Ophthalmol. Strabismus 1994; 31: 153–6. 7931948 10.3928/0191-3913-19940501-06

[225] Lapidoth M , Ben‐Amitai D , Bhandarkar S , Fried L , Arbiser JL . Efficacy of topical application of eosin for ulcerated hemangiomas. J. Am. Acad. Dermatol. 2009; 60: 350–1. 19150285 10.1016/j.jaad.2008.10.034

[226] Barry RB , Hughes BR , Cook LJ . Involution of infantile haemangiomas after imiquimod 5% cream. Clin. Exp. Dermatol. 2008; 33: 446–9. 18485022 10.1111/j.1365-2230.2007.02676.x

[227] Ho NT , Lansang P , Pope E . Topical imiquimod in the treatment of infantile hemangiomas: a retrospective study. J. Am. Acad. Dermatol. 2007; 56: 63–8. 17190622 10.1016/j.jaad.2006.06.011

[228] Jiang C , Hu X , Ma G *et al* A prospective self‐controlled phase II study of imiquimod 5% cream in the treatment of infantile hemangioma. Pediatr. Dermatol. 2011; 28: 259–66. 21615472 10.1111/j.1525-1470.2011.01520.x

[229] Mao XH , Wang JY , Yan JL . Topical imiquimod treatment of cutaneous vascular disorders in pediatric patients: clinical evaluation on the efficacy and safety. J. Zhejiang Univ. Sci. B 2012; 13: 745–50. 22949365 10.1631/jzus.B1200120 PMC3437372

[230] McCuaig CC , Dubois J , Powell J *et al* A phase II, open‐label study of the efficacy and safety of imiquimod in the treatment of superficial and mixed infantile hemangioma. Pediatr. Dermatol. 2009; 26: 203–12. 19419474 10.1111/j.1525-1470.2008.00857.x

[231] Qiu Y , Ma G , Lin X , Jin Y , Chen H , Hu X . Treating protruding infantile hemangiomas with topical imiquimod 5% cream caused severe local reactions and disfiguring scars. Pediatr. Dermatol. 2013; 30: 342–7. 23046440 10.1111/pde.12002

[232] Qiu Y , Ma G , Yang J *et al* Imiquimod 5% cream versus timolol 0.5% ophthalmic solution for treating superficial proliferating infantile haemangiomas: a retrospective study. Clin. Exp. Dermatol. 2013; 38: 845–50. 23627540 10.1111/ced.12150

[233] Welsh O , Olazaran Z , Gomez M , Salas J , Berman B . Treatment of infantile hemangiomas with short‐term application of imiquimod 5% cream. J. Am. Acad. Dermatol. 2004; 51: 639–42. 15389206 10.1016/j.jaad.2004.04.022

[234] Blatt J , Morrell DS , Buck S *et al* β‐blockers for infantile hemangiomas: a single‐institution experience. Clin. Pediatr. (Phila) 2011; 50: 757–63. 21525081 10.1177/0009922811405517

[235] Cante V , Pham‐Ledard A , Imbert E , Ezzedine K , Léauté‐Labrèze C . First report of topical timolol treatment in primarily ulcerated perineal haemangioma. Arch. Dis. Child Fetal Neonatal Ed. 2012; 97: F155–6. 22190187 10.1136/fetalneonatal-2011-301317

[236] Chakkittakandiyil A , Phillips R , Frieden IJ *et al* Timolol maleate 0.5% or 0.1% gel‐forming solution for infantile hemangiomas: a retrospective, multicenter, cohort study. Pediatr. Dermatol. 2012; 29: 28–31. 22150436 10.1111/j.1525-1470.2011.01664.x

[237] Chambers CB , Katowitz WR , Katowitz JA , Binenbaum G . A controlled study of topical 0.25% timolol maleate gel for the treatment of cutaneous infantile capillary hemangiomas. *Ophthalmic* . Plast. Reconstr. Surg. 2012; 28: 103–6. 10.1097/IOP.0b013e31823bfffb 22410658

[238] Khunger N , Pahwa M . Dramatic response to topical timolol lotion of a large hemifacial infantile haemangioma associated with PHACE syndrome. Br. J. Dermatol. 2011; 164: 886–8. 21158749 10.1111/j.1365-2133.2010.10177.x

[239] Ma G , Wu P , Lin X *et al* Fractional carbon dioxide laser‐assisted drug delivery of topical timolol solution for the treatment of deep infantile hemangioma: a pilot study. Pediatr. Dermatol. 2014; 31: 286–91. 24602019 10.1111/pde.12299

[240] Moehrle M , Léauté‐Labrèze C , Schmidt V , Röcken M , Poets CF , Goelz R . Topical timolol for small hemangiomas of infancy. Pediatr. Dermatol. 2013; 30: 245–9. 22471694 10.1111/j.1525-1470.2012.01723.x

[241] Ni N , Guo S , Langer P . Current concepts in the management of periocular infantile (capillary) hemangioma. Curr. Opin. Ophthalmol. 2011; 22: 419–25. 21730838 10.1097/ICU.0b013e32834994b4

[242] Oranje AP , Janmohamed SR . Madern GC, de Laat PC. Treatment of small superficial haemangioma with timolol 0.5% ophthalmic solution: a series of 20 cases. Dermatology 2011; 223: 330–4. 22179543 10.1159/000334778

[243] Pope E , Chakkittakandiyil A . Topical timolol gel for infantile hemangiomas: a pilot study. Arch. Dermatol. 2010; 146: 564–5. 20479314 10.1001/archdermatol.2010.67

[244] Kunzi‐Rapp K . Topical propranolol therapy for infantile hemangiomas. Pediatr. Dermatol. 2012; 29: 154–9. 22141326 10.1111/j.1525-1470.2011.01615.x

[245] Xu G , Lv R , Zhao Z , Huo R . Topical propranolol for treatment of superficial infantile hemangiomas. J. Am. Acad. Dermatol. 2012; 67: 1210–13. 22516113 10.1016/j.jaad.2012.03.009

[246] Garzon MC , Lucky AW , Hawrot A , Frieden IJ . Ultrapotent topical corticosteroid treatment of hemangiomas of infancy. J. Am. Acad. Dermatol. 2005; 52: 281–286. 15692474 10.1016/j.jaad.2004.09.004

[247] Pandey A , Gangopadhyay AN , Sharma SP , Kumar V , Gupta DK , Gopal SC . Evaluation of topical steroids in the treatment of superficial hemangioma. Skinmed 2010; 8: 9–11. 20839418

[248] Kaplan M , Paller AS . Clinical pearl: use of self‐adhesive, compressive wraps in the treatment of limb hemangiomas. J. Am. Acad. Dermatol. 1995; 32: 117–8. 7822500 10.1016/0190-9622(95)90196-5

[249] Ochi G , Ohkawa H , Kaneko M *et al* [Compression therapy and cryosurgery for hemangiomas in childhood]. Shoni Geka 1992; 24: 539–47. Japanese.

[250] Totsuka Y , Fukuda H , Tomita K . Compression therapy for parotid haemangioma in infants. A report of three cases. J. Craniomaxillofac. Surg. 1988; 16: 366–70. 3204160 10.1016/s1010-5182(88)80081-7

[251] Osaki TH , Jakobiec FA , Mendoza PR , Lee Y , Fay AM . Immunohistochemical investigations of orbital infantile hemangiomas and adult encapsulated cavernous venous lesions (malformation versus hemangioma). Ophthalmic. Plast. Reconstr. Surg. 2013; 29: 183–95. 23584448 10.1097/IOP.0b013e31828b0f1f

[252] Laing EL , Brasch HD , Steel R *et al* Verrucous hemangioma expresses primitive markers. J. Cutan. Pathol. 2013; 40: 391–6. 23379586 10.1111/cup.12078

[253] North PE , Waner M , James CA , Mizeracki A , Frieden IJ , Mihm MC Jr . Congenital nonprogressive hemangioma: a distinct clinicopathologic entity unlike infantile hemangioma. Arch. Dermatol. 2001; 137: 1607–20. 11735711 10.1001/archderm.137.12.1607

[254] North PE , Waner M , Mizeracki A , Mihm MC Jr . GLUT1: a newly discovered immunohistochemical marker for juvenile hemangiomas. Hum. Pathol. 2000; 31: 11–22. 10665907 10.1016/s0046-8177(00)80192-6

[255] North PE , Waner M , Mizeracki A *et al* A unique microvascular phenotype shared by juvenile hemangiomas and human placenta. Arch. Dermatol. 2001; 137: 559–70. 11346333

[256] Leon‐Villapalos J , Wolfe K , Kangesu L . GLUT‐1: an extra diagnostic tool to differentiate between haemangiomas and vascular malformations. Br. J. Plast. Surg. 2005; 58: 348–52. 15780229 10.1016/j.bjps.2004.05.029

[257] Ahrens WA , Ridenour RV 3rd , Caron BL , Miller DV , Folpe AL . GLUT‐1 expression in mesenchymal tumors: an immunohistochemical study of 247 soft tissue and bone neoplasms. Hum. Pathol. 2008; 39: 1519–26. 18620729 10.1016/j.humpath.2008.03.002

[258] Trindade F , Kutzner H , Requena L , Tellechea O , Colmenero I . Microvenular hemangioma‐an immunohistochemical study of 9 cases. Am. J. Dermatopathol. 2012; 34: 810–12. 23169416 10.1097/DAD.0b013e31824d2c27

[259] Sadeghpour M , Antaya RJ , Lazova R , Ko CJ . Dilated lymphatic vessels in tufted angioma: a potential source of diagnostic confusion. Am. J. Dermatopathol. 2012; 34: 400–3. 22307233 10.1097/DAD.0b013e318234e720

[260] Drut RM , Drut R . Extracutaneous infantile haemangioma is also Glut1 positive. J. Clin. Pathol. 2004; 57: 1197–200. 15509684 10.1136/jcp.2003.012682 PMC1770476

[261] Lyons LL , North PE , Mac‐Moune Lai F , Stoler MH , Folpe AL , Weiss SW . Kaposiform hemangioendothelioma: a study of 33 cases emphasizing its pathologic, immunophenotypic, and biologic uniqueness from juvenile hemangioma. Am. J. Surg. Pathol. 2004; 28: 559–68. 15105642 10.1097/00000478-200405000-00001

[262] Al‐Adnani M , Williams S , Rampling D , Ashworth M , Malone M , Sebire NJ . Histopathological reporting of paediatric cutaneous vascular anomalies in relation to proposed multidisciplinary classification system. J. Clin. Pathol. 2006; 59: 1278–82. 16751300 10.1136/jcp.2006.038240 PMC1860544

[263] Badi AN , Kerschner JE , North PE , Drolet BA , Messner A , Perkins JA . Histopathologic and immunophenotypic profile of subglottic hemangioma: multicenter study. Int. J. Pediatr. Otorhinolaryngol. 2009; 73: 1187–91. 19524305 10.1016/j.ijporl.2009.03.024

[264] Hernandez F , Navarro M , Encinas JL *et al* The role of GLUT1 immunostaining in the diagnosis and classification of liver vascular tumors in children. J. Pediatr. Surg. 2005; 40: 801–4. 15937818 10.1016/j.jpedsurg.2005.01.046

[265] Mo JQ , Dimashkieh HH , Bove KE . GLUT1 endothelial reactivity distinguishes hepatic infantile hemangioma from congenital hepatic vascular malformation with associated capillary proliferation. Hum. Pathol. 2004; 35: 200–9. 14991538 10.1016/j.humpath.2003.09.017

[266] Das KJ , Sharma P , Naswa N *et al* Hybrid SPECT‐CT with 99mTc‐labeled red blood cell in a case of blue rubber bleb nevus syndrome: added value over planar scintigraphy. Diagn. Interv. Radiol. 2013; 19: 41–3. 23255049 10.4261/1305-3825.DIR.5811-12.2

[267] Senturk S , Bilici A , Miroglu TC , Bilek SU . Blue rubber bleb nevus syndrome: imaging of small bowel lesions with peroral CT enterography. Abdom. Imaging 2011; 36: 520–3. 21085955 10.1007/s00261-010-9663-z

[268] Thomson M , Venkatesh K , Elmalik K , van der Veer W , Jaacobs M . Double balloon enteroscopy in children: diagnosis, treatment, and safety. World J. Gastroenterol. 2010; 16: 56–62. 20039449 10.3748/wjg.v16.i1.56 PMC2799917

[269] Agnese M , Cipolletta L , Bianco MA , Quitadamo P , Miele E , Staiano A . Blue rubber bleb nevus syndrome. Acta Paediatr. 2010; 99: 632–5. 19958301 10.1111/j.1651-2227.2009.01608.x

[270] Hansen LF , Wewer V , Pedersen SA , Matzen P , Paerregaard A . Severe blue rubber bleb nevus syndrome in a neonate. Eur. J. Pediatr. Surg. 2009; 19: 47–9. 18629772 10.1055/s-2008-1038367

[271] Yarlagadda R , Menda Y , Graham MM . Tc‐99m red blood cell imaging in a patient with blue rubber bleb nevus syndrome. Clin. Nucl. Med. 2008; 33: 374–376. 18431163 10.1097/RLU.0b013e31816a78e3

[272] Mechri M , Soyer P , Boudiaf M , Duchat F , Hamzi L , Rymer R . Small bowel involvement in blue rubber bleb nevus syndrome: MR imaging features. Abdom. Imaging 2009; 34: 448–51. 18414932 10.1007/s00261-008-9395-5

[273] Certo M , Lopes L , Ramada J . Blue rubber bleb nevus syndrome: manifestations at computed tomography. Acta. Radiol. 2007; 48: 962–6. 17957509 10.1080/02841850701477702

[274] Kopacova M , Tacheci I , Koudelka J , Kralova M , Rejchrt S , Bures J . A new approach to blue rubber bleb nevus syndrome: the role of capsule endoscopy and intra‐operative enteroscopy. Pediatr. Surg. Int. 2007; 23: 693–7. 17205297 10.1007/s00383-006-1843-0

[275] De Bona M , Bellumat A , De Boni M . Capsule endoscopy for the diagnosis and follow‐up of blue rubber bleb nevus syndrome. Dig. Liver Dis. 2005; 37: 451–3. 15893285 10.1016/j.dld.2004.12.014

[276] Place RJ . Blue rubber bleb nevus syndrome: a case report with long‐term follow‐up. Mil. Med. 2001; 166: 728–30. 11515327

[277] Jacob AG , Driscoll DJ , Shaughnessy WJ , Stanson AW , Clay RP , Gloviczki P . Klippel‐Trenaunay syndrome: spectrum and management. Mayo Clin. Proc. 1998; 73: 28–36. 9443675 10.1016/S0025-6196(11)63615-X

[278] Capraro PA , Fisher J , Hammond DC , Grossman JA . Klippel‐Trenaunay syndrome. Plast. Reconstr. Surg. 2002; 109: 2052–60; quiz 2061–2052. 11994613 10.1097/00006534-200205000-00041

[279] Meine JG , Schwartz RA , Janniger CK . Klippel‐Trenaunay‐Weber syndrome. Cutis 1997; 60: 127–32. 9314616

[280] Redondo P , Aguado L , Martinez‐Cuesta A . Diagnosis and management of extensive vascular malformations of the lower limb: part II. Systemic repercussions [corrected], diagnosis, and treatment. J. Am. Acad. Dermatol. 2011; 65: 909–23; quiz 924. 22000871 10.1016/j.jaad.2011.03.009

[281] Gloviczki P , Hollier LH , Telander RL , Kaufman B , Bianco AJ , Stickler GB . Surgical implications of Klippel‐Trenaunay syndrome. Ann. Surg. 1983; 197: 353–62. 6299216 10.1097/00000658-198303000-00017 PMC1352741

[282] McGrory BJ , Amadio PC . Klippel‐Trenaunay syndrome: orthopaedic considerations. Orthop. Rev. 1993; 22: 41–50. 8380635

[283] Takata M , Watanabe K , Matsubara H , Takato K , Nomura I , Tsuchiya H . Lengthening of the normal tibia in a patient with hemihypertrophy caused by Klippel‐Trenaunay‐Weber syndrome: a case report. J. Orthop. Surg. (Hong Kong) 2011; 19: 359–63. 22184171 10.1177/230949901101900320

[284] Servelle M . Klippel and Trenaunay's syndrome. 768 operated cases. Ann. Surg. 1985; 201: 365–73. 2983626 10.1097/00000658-198503000-00020 PMC1250682

[285] Gates PE , Drvaric DM , Kruger L . Wound healing in orthopaedic procedures for Klippel‐Trenaunay syndrome. J. Pediatr. Orthop. 1996; 16: 723–6. 8906641 10.1097/00004694-199611000-00004

[286] Bajaj Y , Hewitt R , Ifeacho S , Hartley BE . Surgical excision as primary treatment modality for extensive cervicofacial lymphatic malformations in children. Int. J. Pediatr. Otorhinolaryngol. 2011; 75: 673–7. 21419500 10.1016/j.ijporl.2011.02.009

[287] Orvidas LJ , Kasperbauer JL . Pediatric lymphangiomas of the head and neck. Ann. Otol. Rhinol. Laryngol. 2000; 109: 411–21. 10778897 10.1177/000348940010900412

[288] Alqahtani A , Nguyen LT , Flageole H , Shaw K , Laberge JM . 25 years' experience with lymphangiomas in children. J. Pediatr. Surg. 1999; 34: 1164–68. 10442614 10.1016/s0022-3468(99)90590-0

[289] Wiegand S , Zimmermann AP , Eivazi B , Sesterhenn AM , Werner JA . Lymphatic malformations involving the parotid gland. Eur. J. Pediatr. Surg. 2011; 21: 242–5. 21455884 10.1055/s-0031-1271810

[290] Chen WL , Zhang B , Wang JG , Ye HS , Zhang DM , Huang ZQ . Surgical excision of cervicofacial giant macrocystic lymphatic malformations in infants and children. Int. J. Pediatr. Otorhinolaryngol. 2009; 73: 833–7. 19324424 10.1016/j.ijporl.2009.02.019

[291] Lei ZM , Huang XX , Sun ZJ , Zhang WF , Zhao YF . Surgery of lymphatic malformations in oral and cervicofacial regions in children. Oral Surg. Oral Med. Oral Pathol. Oral Radiol. Endod. 2007; 104: 338–44. 17428699 10.1016/j.tripleo.2006.12.025

[292] Okazaki T , Iwatani S , Yanai T *et al* Treatment of lymphangioma in children: our experience of 128 cases. J. Pediatr. Surg. 2007; 42: 386–9. 17270554 10.1016/j.jpedsurg.2006.10.012

[293] Hamoir M , Plouin‐Gaudon I , Rombaux P *et al* Lymphatic malformations of the head and neck: a retrospective review and a support for staging. Head Neck 2001; 23: 326–37. 11400236 10.1002/hed.1039

[294] Fageeh N , Manoukian J , Tewfik T , Schloss M , Williams HB , Gaskin D . Management of head and neck lymphatic malformations in children. J. Otolaryngol. 1997; 26: 253–8. 9263895

[295] Padwa BL , Hayward PG , Ferraro NF , Mulliken JB . Cervicofacial lymphatic malformation: clinical course, surgical intervention, and pathogenesis of skeletal hypertrophy. Plast. Reconstr. Surg. 1995; 95: 951–60. 7732142

[296] de Serres LM , Sie KC , Richardson MA . Lymphatic malformations of the head and neck. A proposal for staging. Arch. Otolaryngol. Head Neck Surg. 1995; 121: 577–82. 7727093 10.1001/archotol.1995.01890050065012

[297] Riechelmann H , Muehlfay G , Keck T , Mattfeldt T , Rettinger G . Total, subtotal, and partial surgical removal of cervicofacial lymphangiomas. Arch. Otolaryngol. Head Neck Surg. 1999; 125: 643–8. 10367920 10.1001/archotol.125.6.643

[298] Greinwald J Jr , Cohen AP , Hemanackah S , Azizkhan RG . Massive lymphatic malformations of the head, neck, and chest. J. Otolaryngol. Head Neck Surg. 2008; 37: 169–73. 19128607

[299] Yang Y , Sun M , Ma Q *et al* Bleomycin A5 sclerotherapy for cervicofacial lymphatic malformations. J. Vasc. Surg. 2011; 53: 150–5. 20843632 10.1016/j.jvs.2010.07.019

[300] Alomari AI , Karian VE , Lord DJ , Padua HM , Burrows PE . Percutaneous sclerotherapy for lymphatic malformations: a retrospective analysis of patient‐evaluated improvement. J. Vasc. Interv. Radiol. 2006; 17: 1639–48. 17057006 10.1097/01.RVI.0000239104.78390.E5

[301] Chaudry G , Guevara CJ , Rialon KL *et al* Safety and efficacy of bleomycin sclerotherapy for microcystic lymphatic malformation. Cardiovasc. Intervent. Radiol. 2014; 37: 1476–81. 24938907 10.1007/s00270-014-0932-z

[302] Smith MC , Zimmerman MB , Burke DK *et al* Efficacy and safety of OK‐432 immunotherapy of lymphatic malformations. Laryngoscope 2009; 119: 107–15. 19117316 10.1002/lary.20041

[303] Giguère CM , Bauman NM , Sato Y *et al* Treatment of lymphangiomas with OK‐432 (Picibanil) sclerotherapy: a prospective multi‐institutional trial. Arch. Otolaryngol. Head Neck Surg. 2002; 128: 1137–44. 12365884 10.1001/archotol.128.10.1137

[304] Udagawa A , Yoshimoto S , Matumoto F *et al* A case of facial cavernous lymphangioma: observation from infancy to adulthood. Nihon Togai Gaku Ganmen Geka Gakkaishi 2005; 21: 302–9.

[305] Nagao M , Sasaki S , Furukawa H , Uchiyama E , Yamamoto Y . [Treatments for huge lymphatic malformations of cheek, oral cavity and neck]. Nihon Keisei Geka Gakkai Kaishi 2007; 27: 779–82. Japanese.

[306] Ravindranathan H , Gillis J , Lord DJ . Intensive care experience with sclerotherapy for cervicofacial lymphatic malformations. Pediatr. Crit. Care Med. 2008; 9: 304–9. 18446102 10.1097/PCC.0b013e31817287de

[307] Poonyathalang A , Preechawat P , Jiarakongmun P , Pongpech S . Sclerosing therapy for orbital lymphangioma using sodium tetradecyl sulfate. Jpn. J. Ophthalmol. 2008; 52: 298–304. 18773268 10.1007/s10384-008-0547-5

[308] Emran MA , Dubois J , Laberge L , Al‐Jazaeri A , Butter A , Yazbeck S . Alcoholic solution of zein (Ethibloc) sclerotherapy for treatment of lymphangiomas in children. J. Pediatr. Surg. 2006; 41: 975–9. 16677896 10.1016/j.jpedsurg.2006.01.019

[309] Bai Y , Jia J , Huang XX , Alsharif MJ , Zhao JH , Zhao YF . Sclerotherapy of microcystic lymphatic malformations in oral and facial regions. J. Oral. Maxillofac. Surg. 2009; 67: 251–6. 19138596 10.1016/j.joms.2008.06.046

[310] Shiels WE 2nd , Kang DR , Murakami JW , Hogan MJ , Wiet GJ . Percutaneous treatment of lymphatic malformations. Otolaryngol. Head Neck Surg. 2009; 141: 219–24. 19643255 10.1016/j.otohns.2009.04.001

[311] Nehra D , Jacobson L , Barnes P , Mallory B , Albanese CT , Sylvester KG . Doxycycline sclerotherapy as primary treatment of head and neck lymphatic malformations in children. J. Pediatr. Surg. 2008; 43: 451–60. 18358281 10.1016/j.jpedsurg.2007.10.009

[312] Asonuma K , Inomata Y . [Current strategy and outcome of treatment of lymphangioma in children: the analysis of a survey in the Kyushu and Okinawa area]. Nihon Shoni Geka Gakkai Zasshi 2006; 42: 215–21. Japanese.

[313] Schwarcz RM , Ben Simon GJ , Cook T , Goldberg RA . Sclerosing therapy as first line treatment for low flow vascular lesions of the orbit. Am. J. Ophthalmol. 2006; 141: 333–9. 16458690 10.1016/j.ajo.2005.09.026

[314] Oyama T , Eguchi K , Cho H , Abe H . A variety of orbital lymphangioma treatments: one case treated with orbital decompression therapy and the other case with intralesional injection of OK‐432 therapy. Nihon Ganka Gakkai Zasshi 2009; 113: 732–40. Japanese. 19637816

[315] Cahill AM , Nijs E , Ballah D *et al* Percutaneous sclerotherapy in neonatal and infant head and neck lymphatic malformations: a single center experience. J. Pediatr. Surg. 2011; 46: 2083–95. 22075337 10.1016/j.jpedsurg.2011.07.004

[316] Chaudry G , Burrows PE , Padua HM , Dillon BJ , Fishman SJ , Alomari AI . Sclerotherapy of abdominal lymphatic malformations with doxycycline. J. Vasc. Interv. Radiol. 2011; 22: 1431–5. 21821431 10.1016/j.jvir.2011.06.021

[317] Oliveira C , Sacher P , Meuli M . Management of prenatally diagnosed abdominal lymphatic malformations. Eur. J. Pediatr. Surg. 2010; 20: 302–6. 20577952 10.1055/s-0030-1254149

[318] Won JH , Kim BM , Kim CH , Park SW , Kim MD . Percutaneous sclerotherapy of lymphangiomas with acetic acid. J. Vasc. Interv. Radiol. 2004; 15: 595–600. 15178720 10.1097/01.rvi.0000127899.31047.0e

[319] Shiels WE 2nd , Kenney BD , Caniano DA , Besner GE . Definitive percutaneous treatment of lymphatic malformations of the trunk and extremities. J. Pediatr. Surg. 2008; 43: 136–9: discussion 140. 18206471 10.1016/j.jpedsurg.2007.09.049

[320] Chiappinelli A , Forgues D , Galifer RB . Congenital abdominal cystic lymphangiomas: what is the correct management? J. Matern. Fetal Neonatal Med. 2012; 25: 915–9. 22035252 10.3109/14767058.2011.600364

[321] Muraoka A , Suzuki N , Niwa Y , Komatsu Y , Tagami K . [A case report of an asymptomatic giant retroperitoneal lymphangioma pointed out at a physical examination]. Nihon Rinsho Geka Gakkai Zasshi 2009; 70: 899–905. Japanese.

[322] Oyachi N , Iwashita K , Kubo M . [Diagnosis and management of mesenteric lymphangioma: comparison of prenatal and school‐age diagnosed cases]. Nihon Shoni Geka Gakkai Zasshi 2008; 44: 33–7. Japanese.

[323] Mendez‐Gallart R , Bautista A , Estevez E , Rodriguez‐Barca P . Abdominal cystic lymphangiomas in pediatrics: surgical approach and outcomes. Acta Chir. Belg. 2011; 111: 374–7. 22299324 10.1080/00015458.2011.11680776

[324] Ikeda T , Asai Y , Nango Y *et al* [Childhood abdominal cystic lymphangioma]. Nihon Shoni Geka Gakkai Zasshi 2008; 44: 959–64. Japanese.

[325] Losanoff JE , Kjossev KT . Mesenteric cystic lymphangioma: unusual cause of intra‐abdominal catastrophe in an adult. Int. J. Clin. Pract. 2005; 59: 986–7. 16033626 10.1111/j.1368-5031.2005.00554.x

[326] Uchiyama M , Murata H , Ohtaki M . [Acute abdomen by inflammatory infiltration of retroperitoneal lymphangioma to the duodenum: report of a case and review of cases in childhood]. Nihon Shoni Geka Gakkai Zasshi 2007; 43: 938–44. Japanese.

[327] Bellini C , Ergaz Z , Radicioni M *et al* Congenital fetal and neonatal visceral chylous effusions: neonatal chylothorax and chylous ascites revisited. A multicenter retrospective study. Lymphology 2012; 45: 91–102. 23342929

[328] Matsuo Y , Okada A . The role and use of Sudan Black in the surgical treatment of chylothorax and chylous ascites. Shoni Geka 2001; 33: 186–90. Japanese.

[329] Spagnol L , Conforti A , Valfre L , Morini F , Bagolan P . Preoperative administration of Sudan III and successful treatment of persistent chylous ascites in a neonate. J. Pediatr. Surg. 2011; 46: 994–7. 21616268 10.1016/j.jpedsurg.2011.01.008

[330] Joe K , Kemmotsu H , Mori T , Goto C , Ohkawa H . [Treatment of idiopathic chylous ascites in infants]. Shoni Geka 2001; 33: 134–40. Japanese.

[331] Moreira Dde A , Santos MM , Tannuri AC , Tannuri U . Congenital chylous ascites: a report of a case treated with hemostatic cellulose and fibrin glue. J. Pediatr. Surg. 2013; 48: e17–9. 10.1016/j.jpedsurg.2012.11.031 23414895

[332] Olivieri C , Nanni L , Masini L , Pintus C . Successful management of congenital chylous ascites with early octreotide and total parenteral nutrition in a newborn. BMJ Case Rep. 2012; 2012 10.1136/bcr-2012-006196 PMC4543147 23010459

[333] Huang Y , Zhuang S , Li Y , Liu M , Chen H , Du M . Successful management of congenital chylous ascites in a premature infant using somatostatin analogue. Indian J. Pediatr. 2011; 78: 345–7. 20953848 10.1007/s12098-010-0256-1

[334] Melo‐Filho AA , Souza IJ , Leite CA , Leite RD , Colares JH , Correia JM . Refractory congenital chylous ascites. Indian J. Pediatr. 2010; 77: 1335–7. 20821276 10.1007/s12098-010-0193-z

[335] Karagol BS , Zenciroglu A , Gokce S , Kundak AA , Ipek MS . Therapeutic management of neonatal chylous ascites: report of a case and review of the literature. Acta Paediatr. 2010; 99: 1307–10. 20377539 10.1111/j.1651-2227.2010.01818.x

[336] Kuroiwa M , Toki F , Suzuki M , Suzuki N . Successful laparoscopic ligation of the lymphatic trunk for refractory chylous ascites. J. Pediatr. Surg. 2007; 42: E15–8. 10.1016/j.jpedsurg.2007.02.036 17502170

[337] Antao B , Croaker D , Squire R . Successful management of congenital chyloperitoneum with fibrin glue. J. Pediatr. Surg. 2003; 38: E7–8. 10.1016/j.jpedsurg.2003.08.011 14614734

[338] Nakagawa J , Nakabayashi M , Kikuchi M *et al* [A case of congenital chyloperitoneum improved by prenatal treatment]. Nihon Sanka Fujinka Gakkai Tokyo Chiho Bukai Kaishi 2002; 51: 399–403. Japanese.

[339] Wakisaka M , Kitagawa H , Satoh Y , Nakada K . Congenital chylo‐ascites treated by laparotomy and OK‐432 injection. Shoni Geka 2001; 33: 196–200. Japanese.

[340] Sato H , Okamatsu T , Yatsuzuka M *et al* Chylous ascites resolved by exploratory laparotomy. Shoni Geka 2001; 33: 191–5. Japanese.

[341] Takahashi A , Suzuki N , Kuwano H . Neonatal chylous ascites. Shoni Geka 2001; 33: 144–7. Japanese.

[342] Komuro H . [Endoscopic surgery for chylothorax and chylous ascites]. Shoni Geka 2010; 42: 805–8. Japanese.

[343] Zeidan S , Delarue A , Rome A , Roquelaure B . Fibrin glue application in the management of refractory chylous ascites in children. J. Pediatr. Gastroenterol. Nutr. 2008; 46: 478–81. 18367970 10.1097/MPG.0b013e31815ce5be

[344] Huang Q , Jiang ZW , Jiang J , Li N , Li JS . Chylous ascites: treated with total parenteral nutrition and somatostatin. World J. Gastroenterol. 2004; 10: 2588–91. 15300913 10.3748/wjg.v10.i17.2588 PMC4572170

[345] Nemoto T , Tsuchiya H , Nagashima K . Clinical and experimental analysis for chylothorax and chylous ascites. Shoni Geka 2001; 33: 119–22. Japanese.

[346] Ohtsu K , Ueda Y , Kurihara S , Kawashima M . Intractable chylous ascites. Shoni Geka 2011; 43: 747–50. Japanese.

[347] Ono S , Iwai N , Chiba F , Furukawa T , Fumino S . OK‐432 therapy for chylous pleural effusion or ascites associated with lymphatic malformations. J. Pediatr. Surg. 2010; 45: e7–10. 10.1016/j.jpedsurg.2010.06.010 20850615

[348] Tanaka M , Yokomori K , Kamii Y . Chylous ascites caused by a retroperitoneal lymphangioma: a case report. Shoni Geka 2001; 33: 163–7. Japanese.

[349] Siebert S , Helbling C , Wolff M *et al* Peritoneovenous shunting as palliative treatment in an infant with chylous ascites due to generalised congenital lymphangiectasia. Klin. Padiatr. 2010; 222: 317–8. 20437330 10.1055/s-0030-1247588

[350] Densupsoontorn N , Jirapinyo P , Aanpreung P , Laohapensang M , Parichatikanond P . Congenital chylous ascites: the roles of fibrin glue and CD31. Acta Paediatr. 2009; 98: 1847–9. 19627262 10.1111/j.1651-2227.2009.01432.x

[351] Guvenc BH , Ekingen G , Tuzlaci A , Senel U . Diffuse neonatal abdominal lymphangiomatosis: management by limited surgical excision and sclerotherapy. Pediatr. Surg. Int. 2005; 21: 595–8. 15931532 10.1007/s00383-005-1421-x

[352] Kotera A , Kamagata S , Hirobe S *et al* A case of diffuse lymphangiomatosis with chylothorax and chylous ascites. Shoni Geka 2001; 33: 128–33. Japanese.

[353] Horisawa M , Nishimoto K , Ogura Y , Tainaka T , Matsunaga K , Niinomi N . Generalized lymphatic dysplasia with intermittent chylous discharge from the scrotum: a case report. Shoni Geka 2001; 33: 180–5. Japanese.

[354] Katz MS , Finck CM , Schwartz MZ *et al* Vacuum‐assisted closure in the treatment of extensive lymphangiomas in children. J. Pediatr. Surg. 2012; 47: 367–70. 22325392 10.1016/j.jpedsurg.2011.11.030

[355] Sugito K , Ikeda T , Hagiwara N *et al* [A case of large mesenteric cyst with inflammatory reaction]. Shoni Geka 2001; 33: 1017–20. Japanese.

[356] Chang TS , Ricketts R , Abramowsky CR *et al* Mesenteric cystic masses: a series of 21 pediatric cases and review of the literature. Fetal Pediatr. Pathol. 2011; 30: 40–4. 21204665 10.3109/15513815.2010.505623

[357] Tran NS , Nguyen TL . Laparoscopic management of abdominal lymphatic cyst in children. J. Laparoendosc. Adv. Surg. Tech. A 2012; 22: 505–7. 22568540 10.1089/lap.2012.0003

[358] Boardman SJ , Cochrane LA , Roebuck D , Elliott MJ , Hartley BE . Multimodality treatment of pediatric lymphatic malformations of the head and neck using surgery and sclerotherapy. Arch. Otolaryngol. Head Neck Surg. 2010; 136: 270–6. 20231646 10.1001/archoto.2010.6

[359] Park JG , Aubry MC , Godfrey JA , Midthun DE . Mediastinal lymphangioma: Mayo Clinic experience of 25 cases. Mayo Clin. Proc. 2006; 81: 1197–203. 16970216 10.4065/81.9.1197

[360] Adams MT , Saltzman B , Perkins JA . Head and neck lymphatic malformation treatment: a systematic review. Otolaryngol. Head Neck Surg. 2012; 147: 627–39. 22785242 10.1177/0194599812453552

[361] Leung M , Leung L , Fung D *et al* Management of the low‐flow head and neck vascular malformations in children: the sclerotherapy protocol. Eur. J. Pediatr. Surg. 2014; 24: 97–101. 24008546 10.1055/s-0033-1354585

[362] Ogawa T , Shibayama M , Shimizu T . Clinical analysis of lymphangioma in the neck: the effects of local OK‐432 injection therapy. Jibi Inkoka Rinsho 2010; 103: 249–55. Japanese.

[363] Arimoto Y , Kudo F , Suzuki H . Usefulness of ultrasonography in the differential diagnosis of respiratory distress disorders including infantile paresis of the vocal cords. Shoni Jibi Inkoka 2005; 26: 37–42. Japanese.

[364] Kitagawa H , Kawase H , Wakisaka M *et al* Six cases of children with a benign cervical tumor who required tracheostomy. Pediatr. Surg. Int. 2004; 20: 51–4. 14689216 10.1007/s00383-003-1081-7

[365] Hiki S , Yamataka A , Kobayashi H , Okada Y , Miyano T . [Treatment of lymphangioma in children. A report of 105 cases]. Juntendo Igaku 2003; 48: 476–83. Japanese.

[366] Desir A , Ghaye B , Duysinx B , Dondelinger RF . Percutaneous sclerotherapy of a giant mediastinal lymphangioma. Eur. Respir. J. 2008; 32: 804–6. 18757704 10.1183/09031936.00014407

[367] Kim DW . OK‐432 sclerotherapy of lymphatic malformation in the head and neck: factors related to outcome. Pediatr. Radiol. 2014; 44: 857–62. 24569928 10.1007/s00247-014-2889-0

[368] Niramis R , Watanatittan S , Rattanasuwan T . Treatment of cystic hygroma by intralesional bleomycin injection: experience in 70 patients. Eur. J. Pediatr. Surg. 2010; 20: 178–82. 20178075 10.1055/s-0030-1247548

[369] Kudo F , Arimoto Y , Nakano A . [Sclerosing therapy for cystic hygroma in infants: intracystic injection of OK‐432]. Tokeibu Geka 2008; 18: 71–5. Japanese.

[370] Kim MG , Kim SG , Lee JH , Eun YG , Yeo SG . The therapeutic effect of OK‐432 (picibanil) sclerotherapy for benign neck cysts. Laryngoscope 2008; 118: 2177–81. 19029851 10.1097/MLG.0b013e3181864acf

[371] Baskota DK , Singh BB , Sinha BK . OK‐432: an effective sclerosing agent for the treatment of lymphangiomas of head and neck. Kathmandu Univ. Med. J. (KUMJ) 2007; 5: 312–7. 18604046

[372] Jamal N , Ahmed S , Miller T *et al* Doxycycline sclerotherapy for pediatric head and neck macrocystic lymphatic malformations: a case series and review of the literature. Int. J. Pediatr. Otorhinolaryngol. 2012; 76: 1127–31. 22572407 10.1016/j.ijporl.2012.04.015

[373] Tomemori T , Kudo F , Sasamura Y , Numata T . [Cystic hygroma in infants]. Tokeibu Shuyo 2003; 29: 58–63. Japanese.

[374] Dasgupta R , Adams D , Elluru R , Wentzel MS , Azizkhan RG . Noninterventional treatment of selected head and neck lymphatic malformations. J. Pediatr. Surg. 2008; 43: 869–73. 18485956 10.1016/j.jpedsurg.2007.12.029

[375] Hogeling M , Adams S , Law J , Wargon O . Lymphatic malformations: clinical course and management in 64 cases. Australas. J. Dermatol. 2011; 52: 186–90. 21834813 10.1111/j.1440-0960.2011.00777.x

[376] Acevedo JL , Shah RK , Brietzke SE . Nonsurgical therapies for lymphangiomas: a systematic review. Otolaryngol. Head Neck Surg. 2008; 138: 418–24. 18359347 10.1016/j.otohns.2007.11.018

[377] Kim KH , Sung MW , Roh JL , Han MH . Sclerotherapy for congenital lesions in the head and neck. Otolaryngol. Head Neck Surg. 2004; 131: 307–16. 15365552 10.1016/j.otohns.2004.02.018

[378] Catalfamo L , Nava C , Lombardo G , Iudicello V , Siniscalchi EN , Saverio PF . Tongue lymphangioma in adult. J. Craniofac. Surg. 2012; 23: 1920–2. 23172444 10.1097/SCS.0b013e31826cf6e3

[379] Magoshi S , Okada M , Shigematsu H , Suzuki S , Kusama K , Sakashita H . [A case of hemangio‐lymphangioma of the tongue]. Nihon Koku Shindan Gakkai Zasshi 2003; 16: 250–2. Japanese.

[380] Ogiuchi H , Yamazaki T , Yamamura T , Kuwazawa T , Ogiuchi H . A long‐term follow‐up, case of lymphangioma of tongue and floor of the mouth. Shoni Koku Geka 2003; 13: 17–20. Japanese.

[381] Rowley H , Perez‐Atayde AR , Burrows PE , Rahbar R . Management of a giant lymphatic malformation of the tongue. Arch. Otolaryngol. Head Neck Surg. 2002; 128: 190–94. 11843730 10.1001/archotol.128.2.190

[382] Chakravarti A , Bhargava R . Lymphangioma circumscriptum of the tongue in children: successful treatment using intralesional bleomycin. Int. J. Pediatr. Otorhinolaryngol. 2013; 77: 1367–9. 23732020 10.1016/j.ijporl.2013.05.009

[383] Wiegand S , Eivazi B , Zimmermann AP *et al* Microcystic lymphatic malformations of the tongue: diagnosis, classification, and treatment. Arch. Otolaryngol. Head Neck Surg. 2009; 135: 976–83. 19841334 10.1001/archoto.2009.131

[384] Hong JP , Lee MY , Kim EK , Seo DH . Giant lymphangioma of the tongue. J. Craniofac. Surg. 2009; 20: 252–4. 19165040 10.1097/SCS.0b013e3181843257

[385] Azizkhan RG , Rutter MJ , Cotton RT , Lim LH , Cohen AP , Mason JL . Lymphatic malformations of the tongue base. J. Pediatr. Surg. 2006; 41: 1279–84. 16818063 10.1016/j.jpedsurg.2006.03.044

[386] Ogawa‐Ochiai K , Sekiya N , Kasahara Y *et al* A case of mediastinal lymphangioma successfully treated with Kampo medicine. J. Altern. Complement. Med. 2011; 17: 563–5. 21568721 10.1089/acm.2010.0562

[387] Roy S , Reyes S , Smith LP . Bipolar radiofrequency plasma ablation (Coblation) of lymphatic malformations of the tongue. Int. J. Pediatr. Otorhinolaryngol. 2009; 73: 289–93. 19081145 10.1016/j.ijporl.2008.10.022

[388] Kemmotsu T , Takeda Y , Nakamura K , Tateishi I . [A case of congenital chylothorax requiring early OK‐432 pleurodesis]. Nihon Shusanki Shinseiji Igakkai Zasshi 2012; 48: 945–50. Japanese.

[389] Tani G , Okuyama H , Kubota A , Kawahara H . [Thoracoscopic thoracic duct ligation in a premature infant with congenital chylothorax]. Nihon Shoni Geka Gakkai Zasshi 2011; 47: 844–7. Japanese.

[390] Miura K , Yoshizawa K , Tamaki M , Okumura K , Okada M . [Congenital chylothorax treated with video‐assisted thoracic surgery]. Kyobu Geka 2008; 61: 1149–51. Japanese. 19068706

[391] Amagai T , Nakamura H , Kaneko M , Sugiura M , Hamada H . Use and problems of pleuro‐peritoneal shunting tube for neonatal chylothorax. Shoni Geka 2001; 33: 201–7. Japanese.

[392] Cleveland K , Zook D , Harvey K , Woods RK . Massive chylothorax in small babies. J. Pediatr. Surg. 2009; 44: 546–50. 19302856 10.1016/j.jpedsurg.2008.08.008

[393] Buttiker V , Fanconi S , Burger R . Chylothorax in children: guidelines for diagnosis and management. Chest 1999; 116: 682–7. 10492271 10.1378/chest.116.3.682

[394] Kaji M , Sakauchi M , Yoshii K *et al* [A case of chylothorax treated effectively with surgery]. Tokyo Joshi Ika Daigaku Zasshi 2013; 83: E366–70. Japanese.

[395] Haga T , Toida C , Muguruma T , Fujino A . [Two cases of mediastinal lymphangiomatosis that required intensive care management]. Nihon Shonika Gakkai Zasshi 2013; 117: 1483–8. Japanese.

[396] Chen YL , Lee CC , Yeh ML , Lee JS , Sung TC . Generalized lymphangiomatosis presenting as cardiomegaly. J. Formos. Med. Assoc. 2007; 106: S10–4. 17493902 10.1016/s0929-6646(09)60359-4

[397] Pfleger A , Schwinger W , Maier A , Tauss J , Popper HH , Zach MS . Gorham‐Stout syndrome in a male adolescent‐case report and review of the literature. J. Pediatr. Hematol. Oncol. 2006; 28: 231–3. 16679920 10.1097/01.mph.0000203721.83566.e6

[398] Noda M , Endo C , Hoshikawa Y *et al* Successful management of intractable chylothorax in Gorham‐Stout disease by awake thoracoscopic surgery. Gen. Thorac. Cardiovasc. Surg. 2013; 61: 356–8. 22865280 10.1007/s11748-012-0130-3

[399] Fukahori S , Tsuru T , Asagiri K *et al* Thoracic lymphangiomatosis with massive chylothorax after a tumor biopsy and with disseminated intravenous coagulation–lymphoscintigraphy, an alternative minimally invasive imaging technique: report of a case. Surg. Today 2011; 41: 978–82. 21748615 10.1007/s00595-010-4383-0

[400] Brodszki N , Lansberg JK , Dictor M *et al* A novel treatment approach for paediatric Gorham‐Stout syndrome with chylothorax. Acta Paediatr. 2011; 100: 1448–53. 21605166 10.1111/j.1651-2227.2011.02361.x

[401] Deveci M , Inan N , Corapcioglu F , Ekingen G . Gorham‐Stout syndrome with chylothorax in a six‐year‐old boy. Indian J. Pediatr. 2011; 78: 737–9. 21188554 10.1007/s12098-010-0328-2

[402] Seok YK , Cho S , Lee E . Early surgical management of chylothorax complicated by Gorham's disease. Thorac. Cardiovasc. Surg. 2010; 58: 492–3. 21110275 10.1055/s-0030-1250158

[403] Kose M , Pekcan S , Dogru D *et al* Gorham‐Stout Syndrome with chylothorax: successful remission by interferon alpha‐2b. Pediatr. Pulmonol. 2009; 44: 613–5. 19434689 10.1002/ppul.20849

[404] Boyle MJ , Alison P , Taylor G , Lightbourne BA . A case of Gorham's disease complicated by bilateral chylothorax. Heart Lung Circ. 2008; 17: 64–6. 17822955 10.1016/j.hlc.2007.01.009

[405] Burgess S , Harris M , Dakin C , Borzi P , Ryan C , Cooper D . Successful management of lymphangiomatosis and chylothorax in a 7‐month‐old infant. J. Paediatr. Child Health 2006; 42: 560–2. 16925547 10.1111/j.1440-1754.2006.00924.x

[406] Underwood J , Buckley J , Manning B . Gorham disease: an intraoperative case study. AANA J. 2006; 74: 45–8. 16483068

[407] Fujiu K , Kanno R , Suzuki H , Nakamura N , Gotoh M . Chylothorax associated with massive osteolysis (Gorham's syndrome). Ann. Thorac. Surg. 2002; 73: 1956–7. 12078801 10.1016/s0003-4975(02)03413-6

[408] Chavanis N , Chaffanjon P , Frey G , Vottero G , Brichon PY . Chylothorax complicating Gorham's disease. Ann. Thorac. Surg. 2001; 72: 937–9. 11565695 10.1016/s0003-4975(00)02417-6

[409] Konez O , Vyas PK , Goyal M . Disseminated lymphangiomatosis presenting with massive chylothorax. Pediatr. Radiol. 2000; 30: 35–7. 10663507 10.1007/s002470050010

[410] Morita K , Fukumoto K , Mitsunaga M *et al* [A case of thoracic lymphangiomatosis causing difficulty in treatment due to dyspnea and hemorrhage]. Nihon Shoni Ketsueki Gan Gakkai Zasshi 2013; 50: 644–9. Japanese.

[411] Kitami A , Suzuki T , Suzuki S , Usuda R , Kamio Y , Kadokura M . Gorham's disease complicated by chyloma of the chest wall. Jpn. J. Thorac. Cardiovasc. Surg. 2006; 54: 311–3. 16898648 10.1007/pl00022261

[412] Lee S , Finn L , Sze RW , Perkins JA , Sie KC . Gorham Stout syndrome (disappearing bone disease): two additional case reports and a review of the literature. Arch. Otolaryngol. Head Neck Surg. 2003; 129: 1340–3. 14676163 10.1001/archotol.129.12.1340

[413] Reinglas J , Ramphal R , Bromwich M . The successful management of diffuse lymphangiomatosis using sirolimus: a case report. Laryngoscope 2011; 121: 1851–4. 22024836 10.1002/lary.21927

[414] Tamay Z , Saribeyoglu E , Ones U *et al* Diffuse thoracic lymphangiomatosis with disseminated intravascular coagulation in a child. J. Pediatr. Hematol. Oncol. 2005; 27: 685–7. 16344679 10.1097/01.mph.0000193476.14493.06

[415] Duffy BM , Manon R , Patel RR , Welsh JS . A case of Gorham's disease with chylothorax treated curatively with radiation therapy. Clin. Med. Res. 2005; 3: 83–6. 16012125 10.3121/cmr.3.2.83 PMC1183437

[416] Kinnier CV , Eu JP , Davis RD , Howell DN , Sheets J , Palmer SM . Successful bilateral lung transplantation for lymphangiomatosis. Am. J. Transplant. 2008; 8: 1946–50. 18671675 10.1111/j.1600-6143.2008.02340.x PMC3732029

[417] Huang SY , Lee YM , Tzeng ST *et al* Gorham syndrome with postoperative respiratory failure and requiring prolonged mechanical ventilation. Respir. Care 2013; 58: e144–8. 23550170 10.4187/respcare.02355

[418] Lee WS , Kim SH , Kim I *et al* Chylothorax in Gorham's disease. J. Korean Med. Sci. 2002; 17: 826–9. 12483010 10.3346/jkms.2002.17.6.826 PMC3054959

[419] Fontanesi J . Radiation therapy in the treatment of Gorham disease. J. Pediatr. Hematol. Oncol. 2003; 25: 816–7. 14528108 10.1097/00043426-200310000-00016

[420] Yoo SY , Goo JM , Im JG . Mediastinal lymphangioma and chylothorax: thoracic involvement of Gorham's disease. Korean J. Radiol. 2002; 3: 130–2. 12087203 10.3348/kjr.2002.3.2.130 PMC2713836

[421] Timke C , Krause MF , Oppermann HC , Leuschner I , Claviez A . Interferon alpha 2b treatment in an eleven‐year‐old boy with disseminated lymphangiomatosis. Pediatr. Blood Cancer 2007; 48: 108–11. 16007599 10.1002/pbc.20461

